# Fifteen Years of NOVA Food-Processing Classification: “Friend or Foe” Among Sustainable Diet Indicators? A Scoping Review

**DOI:** 10.1093/nutrit/nuae207

**Published:** 2025-01-23

**Authors:** Orsolya Tompa, Anna Kiss, Sándor Soós, Zoltán Lakner, Ana Raner, Gyula Kasza, Dávid Szakos

**Affiliations:** Department of Science Policy and Scientometrics, Library and Information Centre of the Hungarian Academy of Sciences, Budapest, 1051, Hungary; Department of Science Policy and Scientometrics, Library and Information Centre of the Hungarian Academy of Sciences, Budapest, 1051, Hungary; Pro-Sharp Research and Innovation Centre, Budapest, 1145, Hungary; Department of Science Policy and Scientometrics, Library and Information Centre of the Hungarian Academy of Sciences, Budapest, 1051, Hungary; Faculty of Education and Psychology, ELTE Eötvös Loránd University, Budapest, 1075, Hungary; Department of Agricultural Business and Economics, Institute of Agricultural and Food Economics, Hungarian University of Agriculture and Life Sciences, Budapest, 1118, Hungary; National Institute of Public Health Slovenia, Ljubljana, 1000, Slovenia; Institute of Food Chain Science, Department of Applied Food Sciences, University of Veterinary Medicine, Budapest, 1078, Hungary; Institute of Food Chain Science, Department of Applied Food Sciences, University of Veterinary Medicine, Budapest, 1078, Hungary

**Keywords:** NOVA classification, sustainable diets, ultra-processed foods, diet quality, dietary environmental impact

## Abstract

It has been 15 years since the introduction of the NOVA food-processing classification. While it was designed to identify ultra-processed foods linked to noncommunicable diseases, the NOVA system has a holistic concept that fits with sustainable nutrition. However, NOVA’s connection to other sustainable diet indicators has not been thoroughly explored. The aim was to summarize the research and methodological context of using the NOVA system with other sustainable diet indicators and to investigate NOVA’s synergies and discordance with them. A scoping review was conducted based on the Preferred Reporting Items for Systematic Reviews and Meta-Analyses—Extension for Scoping Reviews (PRISMA-ScR). Studies published between 2009 and 2023 were collected from the Web of Science, Scopus, and PubMed databases. 1612 studies were initially screened; in the selected studies (n = 77), the NOVA system was applied in addition to other sustainable diet indicator(s). The studies were analyzed within a qualitative data analysis framework. 77 studies were analyzed in which healthiness (n = 66), environmental pressure (n = 9), affordability (n = 11), other processing classifications (n = 6), and other sustainable diet indicators (n = 10) were applied with NOVA. Among them, the identified relationships between the NOVA system and other healthfulness indicators were synergistic in the majority of studies (n = 70/93). For environmental pressure indicators, a mixed picture was observed; the NOVA classification was predominantly synergistic with greenhouse gas emissions (n = 8/13), while it was mostly discordant with fresh water use (n = 8/12). Economic affordability was also found to be discordant with the NOVA classification in the majority of studies (n = 10/16). To complete the NOVA classification with nutrient profiling would be beneficial to identify healthy diets. In the case of the integration of NOVA into sustainable nutrition research, environmental pressure and economic affordability indicators should be controlled to reach optimal trade-offs for more sustainable diets. The application of NOVA is gaining relevance; thus, its methodological adaptation to sustainable nutrition research is necessary.

## INTRODUCTION

In 2015, the United Nations described its Sustainable Development Goals, which are connected to food production and consumption in several ways while promoting the emerging field of sustainable nutrition: “Sustainable Healthy Diets are dietary patterns that promote all dimensions of individuals’ health and wellbeing; have low environmental pressure and impact; are accessible, affordable, safe and equitable; and are culturally acceptable.”^1^ This holistic definition radically extended the interpretation of human nutrition beyond healthfulness to economic, social, and environmental contexts. Accordingly, the assessment of sustainable nutrition has become complex and the development of integrated indicators has become a necessity. The approach to solving this “diet problem” has been to combine or integrate reductionist indicators, such as environmental pressure and nutrient-profiling systems; however, discordance in addition to synergies has continuously appeared between dimensions.^2–5^ Among them, the NOVA classification was proposed in 2009 to classify foods based on their degree of processing: “NOVA classifies all foods and food products into four groups according to the extent and purpose of the industrial processing they undergo. It considers all physical, biological, and chemical methods used during the food manufacturing process, including the use of additives.”^6^ The NOVA system targets the identification of ultra-processed foods (UPFs), in addition to 3 other food-group categories, on the extent and purposes of industrial processing.^7^ However, in general, the NOVA system has not become a part of complex sustainable nutrition assessments as an indicator,^4^^,^^8^ even though Fardet and Rock^9^ have proposed a global health–based approach, based on NOVA, incorporating the 3V (végétal [plant], vrai [real], and varié [varied]) holistic and sustainable dietary assessment tool.

Ever since its establishment, the NOVA classification has led to intense supporting arguments and criticism. With regard to the latter, it was pointed out that using the NOVA classification can easily lead to misclassification, thus failing to identify “healthy” and “unhealthy” foods, not being precise enough considering the quantity and quality of ingredients while it adds little to existing food-based dietary guidelines (FBDGs) and nutrient-profiling systems’ ability to identify healthy foods and diets,^10^ and lacks biological plausibility.^11^ Moreover, FoodDrinkEurope has published a position paper stating that “level of processing is not a scientifically sound approach to food policy and would lead to negative outcomes for our food systems.”^12^ In contrast, NOVA classification applied to identify UPFs was also regarded with support: an emphasis on a reduction in UPF intake has appeared in dietary guidelines of (inter)national organizations.^13^^,^^14^ However, James-Martin et al^14^ emphasized that food processing is only addressed in 10 of FBDGs incorporating environmental aspects, although this food group is a major contributor to the environmental pressure of diets. In addition, higher intake of UPFs is linked to lower diet quality, increasing risks of morbidity and mortality of several noncommunicable diseases (NCDs) based on solid scientific evidence.^6^^,^^15–18^ In fact, the World Health Organization (WHO)^19^ placed UPFs among 4 of the most impactful commercial determinants of health in the WHO European region, related to NCD onset and NCD-related deaths, suggesting the need for more intensive policy actions addressing UPFs for re-shaping the food environment. NOVA is a holistic indicator capable of capturing effects (ie, the matrix effect) that cannot be derived from nutrient-based approaches^20^^,^^21^ and stands out among other sustainable diet indicators (SDIs) with its empirico-inductive approach.^22^ In addition, the NOVA classification assesses the natural aspect of foods that is important in terms of facilitating the sense of connectedness between humans and the natural environment into 1 system,^23^^,^^24^ an aspect emphasized by the definition of sustainable nutrition.

It has been described that the combination of NOVA (as a holistic approach) with nutrition-profiling systems (as a reductionist approach) is an “indispensable marriage”^20^ but the former should serve as a starting point. Moreover, these were described as complementary indicators,^25^^,^^26^ and the Nutri-Score V2.0, including the UPF component, has already been proposed,^26^ while, similarly, the Siga approach complemented NOVA with the consideration of nutrient content.^27^ However, according to the position paper of the British Dietetic Association, the nutritional profile of foods is more important than the level of processing; thus, UPFs can be beneficial in certain cases and they are not necessarily high in salt, sugar, or fat.^28^ Studies on the association of NOVA classification with environmental pressure showed a mixed picture.^29^ Furthermore, it seems well proven that a diet higher in UPFs is purposefully cheaper.^30^^,^^31^ While the information on food labels has become crowded,^32^ the level of processing and its link to healthfulness have become intuitive to consumers.^33^ According to the report of European Institute of Innovation and Technology Food (EIT Food), consumers are concerned about UPFs’ long-term health effect and sustainability; however, they lack the willingness to reduce UPF intake, as UPFs offer convenient, tasty, and low-cost food options.^30^

Consequently, new food policies targeting UPFs seem unavoidable,^34^ especially due to their link to the development of NCDs.^6^^,^^17–19^ However, the implementation of novel policies on UPF intake will likely trigger arguments among stakeholders^35^; thus, the use of an adequate tool—such as NOVA—to identify UPFs and estimate their relation to other SDIs is necessary.

In sum, the NOVA system's holistic concept fits well in the sustainable nutrition approach, its main target; UPFs have an evidence-based link to negative health outcomes and NCDs and they have an ongoing consideration in food policy formation. Still, the NOVA system is not generally applied in sustainable nutrition research, the effect of food processing on sustainability has not been explored well enough,^36^ its relation with other SDIs has not been explored from a complex point of view, and its use in sustainable nutrition studies needs more methodological clarification.

The study aims to summarize the research and methodological context in which the NOVA system is applied with other SDIs, pointing out their advantages and shortcomings in nutritional science studies. In addition, this study aims to synthesize and analyze evidence on the NOVA system's synergies and discordances with other SDIs, thus estimating the NOVA system's relation to them. To our knowledge, this is the first review focusing on the NOVA classification as a method and its role in sustainable nutrition research. As a scoping review, it aims to explore this topic from a global perspective to provide insights for applying NOVA in sustainable nutrition research and implications for food and public health policy issues. Thus, the research questions are as follows:

1. What are the characteristics of publication trends and the research context in which the NOVA system is applied with other SDIs?
2. What is the existing methodological framework in which the NOVA classification is applied and analyzed in sustainable nutrition research?
3. What is the relationship (synergies and discordances) of the NOVA classification compared with other SDIs in nutritional science studies?
4. What are the key findings and limitations that originated in the application of NOVA compared with other SDIs?

## METHODS

A scoping review was conducted to assess the role of NOVA classification among complex SDIs. A scoping review was chosen as the most suitable method due to the nature of the research questions, which involves exploring the methodological framework, and characteristics of available data, clarifying concepts within the field of study, and the objective of identifying systematic knowledge gaps.^37^ The development of the protocol and reporting followed the Preferred Reporting Items for Systematic Reviews and Meta-Analyses—Extension for Scoping Reviews (PRISMA-ScR) checklist and guidelines^38^ (Appendix S1).^38^

### Data Sources and Search Strategy

The search strategy was developed based on 3 main components (and their synonyms) according to the research questions: “NOVA classification,” “assessment,” and “diet.” Details of the search strategy are provided in Appendix S2.^39^ For the literature search, the most comprehensive databases were used—PubMed, Web of Science, and Scopus—as they are considered primary international electronic repositories of scientific publications. Data collection covered the period from the development of the NOVA classification in 2009^21^ to the end of November 2023, including studies with full texts and DOI numbers included. The search encompassed titles, abstracts, and author key words, aiming to identify relevant publications regarding the application of the NOVA classification, particularly within the context of sustainable dietary quality assessment.

### Eligibility Criteria

Eligible studies incorporated either complex dietary quality (“healthfulness”) or other SDIs in addition to the NOVA classification—in other words, applied 2 or more indicators (including NOVA) for assessment. This criterion was defined to fulfill the sustainable nutrition concept, which includes multiple indicators in an assessment. Complex dietary quality indicators were defined as metrics that integrate multiple nutrients (with or without incorporating food components), such as nutrient-profiling models, and thus excluded studies only focusing on energy or single-nutrient intake. Additionally, studies that utilized other indicators to measure the degree of food processing were also included. Furthermore, studies were excluded if the applied indicators were not related to food or diet, such as anthropometric or sociodemographic indicators. In addition, studies focusing on UPF intake were excluded if they lacked results or conclusions of interest regarding the NOVA classification. Last, studies analyzing solely diet therapy–specific food items were also excluded from this scoping review.

No restrictions were applied regarding the target populations, age groups, countries, or genders in the selection of published studies. Only peer-reviewed, DOI (Digital Object Identifier)–numbered, full-text, English-language articles were included. The sample reflected a large variety of study designs: cohort, cross-sectional, prospective, longitudinal, surveys, interventions, and time-series studies. Reviews, book chapters, conference proceedings, duplicates, and non–peer-reviewed publications were excluded from the analysis.

### Screening and Data Extraction

Two members of the research team (O.T. and A.K.) assessed titles, abstracts, and full texts of articles; determined the eligibility of articles; and extracted data from eligible papers. Both researchers independently extracted data from a distinct set of studies, compared their findings, then deliberated on the findings until they reached consensus on data interpretation. In cases where consensus was not reached, a third researcher was consulted. No quality assessment was performed for this scoping review, as it aimed to summarize and evaluate methodological issues related to the topic to guide future research and practice, without a quality criterion.

Data extraction was performed utilizing a standardized Microsoft Excel template (Microsoft Corporation, Redmond, WA, USA), using the following details: author, year, location, population, sample size, study design, age, gender, relevant statistics, specific characteristics of the dietary assessment, and results and conclusions relevant to the study aims. Additionally, bibliographic data from publications were extracted to perform a bibliometric analysis aimed at understanding the structure and dynamics of research related to NOVA with other SDIs. Data extraction was carried out with the use of Microsoft Excel and HubScience Research Intelligence Software (Budapest, Hungary).^40^

### Synthesis of Results

The analysis of the studies was conducted based on qualitative data synthesis. The reviewed articles were evaluated with a focus on methodology and research perspectives. First, the general aspects (eg, study design, aim of the study) of the reviewed studies were summarized to present the research context in which NOVA classification was applied. Second, results were synthesized according to the following research aims and questions:

1. Characteristics of the methodological approach: source and type of dietary data, level of analysis, data aggregation, variables calculated based on NOVA classification, NOVA groups as the basis of comparisons, and other applied SDIs.
2. Summary on the proportion of foods classified as UPF (ie, NOVA G4) in the analyzed diet or set of foods. The studies included in this analysis had relevant results for the proportion of NOVA groups, precisely the G4, in the analyzed set of foods or diets. Proportions were visualized with 100% aggregated column diagrams with the division of UPFs and non-UPFs in the sample, marking food- and diet-level analysis as groups separately.
3. To show the results on the NOVA system classification’s relation to other SDIs, besides textual summary, visual tables were also created to summarize the patterns of the identified association (synergies, discordances, and inconclusive links) between the NOVA system and other SDIs by categories: dietary quality (ie, “healthfulness”), environmental pressure, economic affordability, other degree of processing classification system, and other SDIs. NOVA groups were categorized as G1, G2, G3, and G4, defined as follows: group 1 (unprocessed and minimally processed foods [MPFs]), group 2 (processed culinary ingredients), group 3 (processed foods), and group 4 (UPFs) based on Monteiro et al^6^. Synergy and discordance were interpreted as effects towards more sustainable diets. For this analysis, synergy and discordance were defined as follows: A synergistic relationship was assumed to have better qualities—for example, “healthier” or lower in price or lower environmental pressure in association with lower NOVA G4 or higher NOVA G1 or NOVA G3 proportion in the diet/set of foods. In other words, in the case of a synergistic relation, indicators simultaneously are directed towards more sustainable diets. A discordant relationship was assumed as having worse qualities (eg, a higher price or environmental pressure) in association with lower NOVA G4 or higher NOVA G1 or NOVAG3 proportion in the diet/set of food. In other words, in the case of discordant relations, the indicators are inversely linked to more sustainable diets. Since NOVA G4 is considered “unhealthy” by the evidence and linked to the development of NCDs,^17^^,^^18^ the distinction was made based on the NOVA G4 and non–NOVA G4 (NOVA G1 and G3) classification. A synergistic or discordant relationship was not assumed based on NOVA G2 since these foods are culinary ingredients with cooking technological purposes and their nutrient profile is not comparable to NOVA G1, G3, or G4. These relationships do not correspond to the mathematical direction of the association. Energy density (ED) was considered as worse quality if higher and as better quality if lower, since ED is usually among the negative components of diet quality scores.^4^ A visual presentation of analyzed relationships between NOVA and other SDIs is shown in Appendix S3.

### Bibliometric Analysis

Bibliometric analysis was carried out using publication metadata to describe research trends that apply NOVA classification with other SDIs. This analysis included trends in total and average citations, key publication channels (journal), distribution of research output by countries and years, as well as relevant key words and their co-occurrences. The integration of the NOVA classification into the sustainable nutrition research context through the co-occurrence network of key words was visualized. The analysis was conducted by 1 researcher (O.T.) using the Bibliometrix R package (R Foundation for Statistical Computing, Vienna, Austria).^41^ (The complete description and visualization of results are shown in Appendix S4.)^9^^,^^25^^,^^29^^,^^31^^,^^33^^,^^42–113^

### Qualitative Analyses of Limitations and Key Findings

A qualitative content analysis was applied to identify limitations related to NOVA in the reviewed articles, as well as to summarize the key findings on the NOVA system's related topics, using open and inductive coding; then, the subcategories and categories were created. Two researchers coded the same dataset independently using Microsoft Excel (Microsoft Corporation, Redmond, WA, USA). One of the authors (O.T.) systematically coded all data to identify subcategories by using meaningful segments and key words, from which categories emerged by grouping common and recurring ideas. Codes were then inductively renamed and reorganized to further refine subcategories and categories. Another member of the research team (A.K.) independently went through the same coding process. The 2 authors compared and discussed their results until they reached a consensus on the interpretation of the data. Any discrepancies in code assignments were resolved by discussing the rationale behind each decision and agreeing on a coding assignment.

## RESULTS

### PRISMA Screening Results

Overall, 1612 records were initially identified through the database search. After removing duplicates and studies without DOI numbers, 1031 records remained. Following a screening of titles and abstracts among these 1031 records, 713 were excluded due to irrelevant content. Among the remaining 318 records evaluated for eligibility, 241 were excluded because the full-text version was inaccessible (*n* = 8), written in a language other than English (*n* = 25), or did not meet the other inclusion criteria (*n* = 208). Ultimately, 77 studies met the eligibility criteria according to the results of the literature search and selection process (Appendix S2).

### General Characteristics of the Reviewed Studies

Most studies were conducted in Europe (*n* = 33) and North America (*n* = 16) (Table 1^9^^,25,29,31,33,^^42–113^; Table S1^9^^,^^25^^,^^29^^,^^31^^,^^33^^,^^42–113^). Within Europe, the studies were predominantly conducted in France^9^^,^^54^^,^^58^^,^^69^^,^^77^^,^^80^^,^^98^^,^^102^^,^^109^ and Spain.^60^^,^^61^^,^^62^^,^^71^^,^^86^^,^^87^^,^^88^^,^^92^^,^^97^ Twelve studies were from Latin America^31^^,^^48^^,^^55^^,^^59^^,^^70^^,^^72^^,^^83^^,^^85^^,^^89^^,^^90^^,^^99^^,^^107^ (among them 9 from Brazil^31^^,^^48^^,^^59^^,^^72^^,^^83^^,^^85^^,^^89^^,^^90^^,^^99^), 9 studies were from Australia,^46^^,^^57^^,^^66^^,^^68^^,^^73^^,^^84^^,^^96^^,^^105^^,^^113^ 4 were from Central/East Asia,^56^^,^^94^^,^^95^^,^^103^ and 2 were from Ethiopia.^49^^,^^106^ The key target groups were culturally, ethnically or professionally specific (*n* = 21)^33^^,^^49^^,^^50^^,^^54^^,^^61^^,^^65^^,^^67^^,^^71^^,^^72^^,^^74^^,^^81^^,^^82^^,^^85^^,^^86^^,^^87^^,^^91^^,^^95^^,^^100^^,^^108^^,^^113^ (eg, indigenous people^70^), the general population (*n* = 9),^9^^,^^53^^,^^55^^,^^68^^,^^73^^,^^77^^,^^98^^,^^101^^,^^111^ and adults and elderly people (*n* = 11),^29^^,^^47^^,^^51^^,^^63^^,^^69^^,^^76^^,^^78^^,^^80^^,^^84^^,^^102^^,^^103^ while a few studies focused on children/adolescents (*n* = 5)^52^^,^^56^^,^^60^^,^^99^^,^^104^^,^^106^ (Table 1; Table S1).

**Table 1.** Summary of General (A) and Specific (B) Dietary Assessment Aspects

A	No.	B	No.
Region		Data source	
Europe	33	List of food products	26
Oceania	9	Health/nutrition/food consumption surveys	40
Latin-America	12		
North America	16	Online survey	4
Asia	4	Secondary data analysis from cohort studies	7
Africa	2	Dietary data	
Study design		24-hour dietary recall	25
Cross-sectional	70	Food frequency questionnaire	16
Prospective	2	Food composition database	8
Longitudinal	2	Food supply, company portfolio	3
Intervention	1	Food label, picture	17
Time-series	1	Household consumption	6
Randomized controlled trial	1	Consumer basket	2
Target group		Dietary level of analysis	
Children/adolescents	5	Diet	39
Adults/elderly	12	Food	39
General population	9	Sustainable dietary indicators	
Household	3	Dietary quality (“healthfulness”) indicators	66
Special target group	21	Environmental pressure indicators	9
No target group-food database	27	Economic affordability indicators	11
		Other degree of processing indicators	6
		Other sustainable diet indicators	10

Abbreviation: No., number of studies.

### Specific Dietary Assessment Aspects

One-third of all datasets originated from food lists, such as online databases, supermarket websites, and food-composition databases (*n* = 26)^25^^,^^31^^,^^42^^,^^44^^,^^45^^,^^46^^,^^48^^,^^57^^,^^62^^,^^64^^,^^66^^,^^88^^,^^89^^,^^92^^,^^93–97^^,^^99^^,^^105–107^^,^^109^^,^^110^^,^^112^). In addition, in 40 studies, the datasets derived from nationally representative health, nutrition, or food-consumption surveys conducted via cross-sectional studies,^43^^,^^47^^,^^49^^,^^50–52^^,^^55^^,^^56^^,^^59^^,^^61^^,^^63^^,^^65^^,^^68^^,^^70–76^^,^^79–87^^,^^90^^,^^91^^,^^98^^,^^102^^,^^103–105^^,^^108^^,^^111^ while, in 4 studies, data were obtained through web-based cross-sectional surveys.^54^^,^^58^^,^^67^^,^^33^ Additionally, in a total of 7 studies^29^^,^^53^^,^^60^^,^^77^^,^^78^^,^^100^^,^^101^ dietary data were collected from cohorts or prospective studies (eg, French NutriNet-Santé cohort^69^) (Table 1; Table S1).

With regard to dietary data, the 24-hour dietary recall (*n* = 25)^47^^,^^49^^,^^51^^,^^55^^,^^56^^,^^63^^,^^68–70^^,^^72^^,^^77^^,^^80–82^^,^^84^^,^^85^^,^^98^^,^^101–104^^,^^106^^,^^108^^,^^111^^,^^113^ was the most common method for collecting dietary data. A comprehensive or national food-composition database (eg, US Department of Agriculture [USDA]), as a primary dietary database, was used in 8 studies for dietary assessment.^9^^,^^25^^,^^42^^,^^43^^,^^46^^,^^94^^,^^95^^,^^105^ Other methods used for dietary data assessment included food-frequency questionnaires (FFQs) (*n* = 16),^29^^,^^50^^,^^53^^,^^60^^,^^65^^,^^67^^,^^71^^,^^73^^,^^74^^,^^75^^,^^76^^,^^78^^,^^86^^,^^87^^,^^91^^,^^100^ food labels and food picture analysis (*n* = 17),^33^^,^^42^^,^^44^^,^^45^^,^^48^^,^^57^^,^^58^^,^^61^^,^^62^^,^^64^^,^^88^^,^^89^^,^^92^^,^^96^^,^^97^^,^^99^^,^^112^ household and other food-consumption data (*n* = 6),^31^^,^^52^^,^^59^^,^^79^^,^^83^^,^^90^ market baskets,^93^^,^^107^ as well as food supply data and company portfolio (*n* = 3).^66^^,^^109^^,^^110^ The most commonly utilized dietary quality indicators included nutrient-profiling models (*n* = 46)^9^^,^^25^^,^^31^^,^^33^^,^^42–48^^,^^52–55^^,^^57^^,^^62–64^^,^^66^^,^^69^^,^^74–77^^,^^80^^,^^83^^,^^88–90^^,^^92–97^^,^^99^^,^^102^^,^^105–107^^,^^109–113^ of which Nutri-Score was the most common (*n* = 11)^25^^,^^33^^,^^42^^,^^44^^,^^54^^,^^62^^,^^66^^,^^92^^,^^107^^,^^109^^,^^110^, followed by ED (*n* = 9).^31^^,^^52^^,^^54^^,^^63^^,^^74^^,^^75^^,^^90^^,^^103^^,^^111^ In addition, food-based dietary quality scores or adherence to dietary guidelines (*n* = 28)^9^^,^^49^^,^^51^^,^^53^^,^^56^^,^^60^^,^^67^^,^^68^^,^^71^^,^^74^^,^^77–82^^,^^84^^,^^85–87^^,^^98^^,^^100^^,^^101^^,^^103–106^^,^^113^ (of which adherence to the Mediterranean diet [*n* = 9]^53^^,^^60^^,^^67^^,^^71^^,^^85–87^^,^^100^^,^^101^ was the most common) were used to measure dietary quality (Table 1). Among environmental pressure indicators, greenhouse gas emissions (GHGEs) were used in 8 studies,^29^^,^^43^^,^^50^^,^^59^^,^^71^^,^^72^^,^^80^^,^^111^ while water use was utilized in 7 studies.^29^^,^^50^^,^^59^^,^^71^^,^^72^^,^^80^^,^^111^ With regard to economic affordability, most commonly dietary cost/food price was incorporated into the analysis of 9 studies^31^^,^^43^^,^^74–76^^,^^83^^,^^90^^,^^93^^,^^108^ (Table 1; Table S1).

#### Level of Food Aggregation Shown in the Results

In most studies (*n* = 66), the results were shown in aggregated food groups, of which the smallest set was 5 and the greatest was 78, while the majority ranged between 10 and 20 (*n* = 22). ^9^^,^^25^^,^^29^^,^^31^^,^^33^^,^^42–113^ The food groups were based on dietary record components (eg, 24-hour or FFQ), FBDG food groups or dietary quality score components, and NOVA food groups that were either separated or integrated into other food groups (Table S1).

#### Variables Calculated From the NOVA Classification Applied in the Studies

With regard to the variables calculated on the basis of NOVA classification, the major applied methods were to classify the 4 NOVA groups on the basis of comparative statistical tests (*n *= 25),^31^^,^^42^^,^^45^^,^^46^^,^^50^^,^^54^^,^^55^^,^^58^^,^^62^^,^^70^^,^^75^^,^^76^^,^^78^^,^^83^^,^^89–91^^,^^93^^,^^94^^,^^97^^,^^99^^,^^105^^,^^107^^,^^111^^,^^112^ estimate the contribution of UPFs (in %) to total dietary energy intake (*n* = 16)^9^^,^^47^^,^^49^^,^^51^^,^^59^^,^^60^^,^^65^^,^^74^^,^^79^^,^^81^^,^^82^^,^^84^^,^^98^^,^^101^^,^^104^^,^^113^ or dietary intake in weight (g) (*n* = 3),^53^^,^^63^^,^^67^ and calculate population segments (*n* = 13)^29^^,^^56^^,^^71–73^^,^^77^^,^^80^^,^^86^^,^^87^^,^^100^^,^^102^^,^^103^^,^^108^ based on UPF (and/or MPF) intake levels. Further solutions to quantify NOVA variables were to identify UPFs, and accordingly, non-UPF share of the analyzed sample (*n* = 10),^48^^,^^57^^,^^66^^,^^68^^,^^88^^,^^95^^,^^96^^,^^106^^,^^109^^,^^110^ classifying NOVA groups with the exclusion of G2 (ie, culinary ingredients) (*n* = 8)^25^^,^^33^^,^^43^^,^^44^^,^^52^^,^^61^^,^^69^^,^^92^ and to calculate the UPF-to-MPF intake ratio (*n* = 1)^85^ (further details are shown in Table S1).

#### Results on the Proportion of Foods Classified as UPFs (ie, NOVA G4) in the Analyzed Diet or Set of Foods

**Figure 1.**
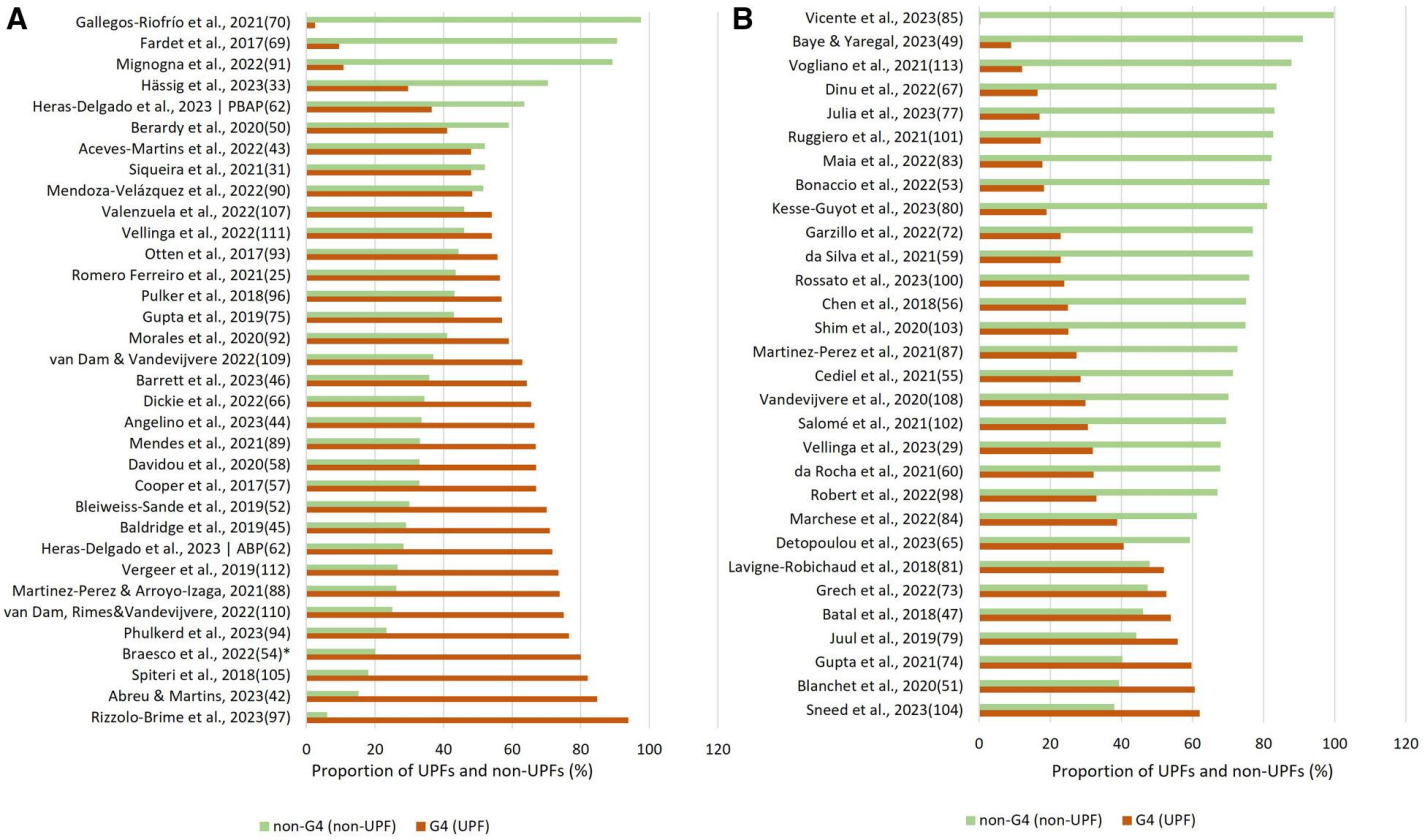
Proportion (%) of UPFs (NOVA G4) and Non-UPFs in the Analyzed Samples on Food-Level (A; *n* = 34) and Diet-Level (B; *n* = 30) Analyses. In the case that there were several datasets by temporal trends, the most recent was considered. Abbreviations: ABP, animal-based products; Non-UPF, non–ultra-processed food; PBAP, plant-based alternative products; UPF, ultra-processed food. *set of marketed foods were considered.

As shown on Figure 1,^25^^,^^29^^,^^31^^,^^33^^,^^42–47^^,^^49^^,^^50–59^^,^^60^^,^^62^^,^^65–67^^,^^69^^,^^70^^,^^72–75^^,^^77^^,^^79^^,^^80–85^^,^^87^^,^^88^^,^^100–105^^,^^107–113^ the contribution of UPFs (%) to the total analyzed sample shows an apparent difference between the food-level (Figure 1A) and diet-level (Figure 1B) analyses. The samples of food-level analysis have larger proportions of UPFs in the samples. The maximum food-level UPF proportion of UPFs is 93.9% (median: 63.6%), while the maximum UPF proportion from dietary assessments (actual intake) is 62% (median: only 28%) (further details are shown in Table S1).

#### Variables of NOVA Shown in Comparative Results

According to the reviewed studies’ designs, the variables shown in the results of comparative analyses were either the different combination of the 4 NOVA groups (*n* = 43)^25^^,^^29^^,^^31^^,^^33^^,^^42–44^^,^^50–52^^,^^54^^,^^56^^,^^59^^,^^60–64^^,^^69^^,^^70^^,^^72^^,^^75–79^^,^^83^^,^^89^^,^^90–93^^,^^96^^,^^97^^,^^99^^,^^100^^,^^102^^,^^104^^,^^105^^,^^107^^,^^108^^,^^111–113^ or the UPF and (non-UPF marked or not) binarity that commonly focused the analyses on the UPF group (*n* = 32).^45–49^^,^^51^^,^^53^^,^^55^^,^^57^^,^^60^^,^^65^^,^^68^^,^^71^^,^^73^^,^^74^^,^^77^^,^^79^^,^^80–82^^,^^84–88^^,^^94^^,^^95^^,^^98^^,^^101^^,^^103^^,^^106^^,^^109^^,^^110^ Of 32 studies, 21 of these studies were diet-level studies,^49^^,^^53^^,^^55^^,^^65^^,^^67^^,^^68^^,^^71^^,^^73^^,^^74^^,^^77^^,^^80–82^^,^^84–86^^,^^98^^,^^101^^,^^103^^,^^106^^,^^113^ and consequently the contribution of UPFs to the total diet was estimated, and 11 studies were food-level analyses^45^^,^^46^^,^^48^^,^^57^^,^^66^^,^^87^^,^^88^^,^^94^^,^^95^^,^^109^^,^^110^ (further details are shown in Table S1).

### Summary of Identified Relations Between NOVA and Other Sustainable Diet Indicators From the Reviewed Studies by Indicator Categories

#### NOVA System’s Relation to Healthfulness Indicators

As presented in Table S2^9^^,^^25^^,^^29^^,^^31^^,^^33^^,^^42–113^ and visualized in Table 2^47,49,51,53,55,56,60,63,67,68,71,74,^^76–87^^,^^98^^,^^100–104^^,^^111^ and Table 3,^25^^,^^31^^,^^33^^,^^42–46^^,^^52^^,^^54^^,^^57^^,^^62^^,^^66^^,^^69^^,^^75^^,^^88–90^^,^^92^^,^^94–96^^,^^99^^,^^107^^,^^111^^,^^112^ among the reviewed studies identifying associations with NOVA and other healthfulness indicators, the overwhelming majority of them showed a synergistic relationship with NOVA. On the diet level, 48 synergistic^49^^,^^51^^,^^53^^,^^55^^,^^56^^,^^60^^,^^63^^,^^67^^,^^71^^,^^74^^,^^76–84^^,^^86^^,^^87^^,^^98^^,^^100–104^^,^^111^ and 1 discordant relationships^68^ were found, while 4 were not significant, inconclusive, or neutral^47^^,^^55^^,^^76^^,^^85^ (Table 2). Similarly, among studies analyzed on the food level, 23 identified as synergistic^25^^,^^31^^,^^33^^,^^42^^,^^43^^,^^44^^,^^52^^,^^54^^,^^69^^,^^75^^,^^89^^,^^90^^,^^95^^,^^96^^,^^111^ and 1 had a discordant relationship^42^; in addition, a notable number of studies were inconclusive, neutral, or not significant (*n* = 11)^44^^,^^45^^,^^52^^,^^54^^,^^57^^,^^62^^,^^92^^,^^95^^,^^107^^,^^111^^,^^112^ (Table 3). With regard to agreement analysis results on food-level analysis, there were 3 “poor,”^94^ 5 “slight,”^66^^,^^99^ 3 “fair,”^46^^,^^66^^,^^99^ and 4 “moderate”^66^^,^^88^^,^^99^ levels of agreement identified between NOVA and other healthfulness indicators targeting to identify “healthy” and “unhealthy” foods. With regard to for the exceptions, Estell et al^68^ applied an “upside-down” approach by excluding UPFs from the diet and concluded discordance of nutritional quality and the NOVA classification system; similarly, Hallinan et al^76^ found inconclusive results when excluding all UPFs from the diet optimization. Patterns also show that, in cases when the components of dietary quality scores were analyzed separately, more inconclusive results were found besides synergy of the total components and NOVA.^52^^,^^55^^,^^95^

**Table 2.** Pattern of Identified Associations Between NOVA and Dietary Quality (“Healthfulness”) Indicators: Diet Level

**Di** **etary quality (“healthfulness”) indicators**	Sneed et al, 2013^104^	Gupta et al, 2021^74^	Martinez-Perez et al, 2021^86^	Shim et al, 2020^103^	Vellinga et al, 2022^111^	Liu et al, 2022^82^	Moraes et al, 2021^63^	Dinu et al, 2022^67^	García et al, 2023^71^	Salomé et al, 2021^102^	Rossato et al, 2023^100^	Batal et al, 2018^47^	Martinez-Perez et al, 2022^87^	Kesse-Guyot et al, 2023^80^	Cediel et al, 2021^55^	da Rocha et al. 2021^60^	Marchese et al, 2022^84^	Julia et al, 2023^77^	Estell et al, 2021^68^	Baye and Yaregal, 2023^49^	Vicente et al, 2023^85^	Juul et al, 2021^78^	Robert et al, 2022^98^	Bonaccio et al, 2022^53^	Ruggiero et al, 2021^101^	Hallinan et al, 2021^76^	Maia et al, 2022^83^	Blanchet et al, 2020^51^	Lavigne-Robichaud et al, 2018^81^	Chen et al, 2018^56^	Juul et al, 2019^79^
HEI	**S**	**S**				**S**					**S**																	**S**	**S**		**S**
NRF		**S**																													
ED		**S**		**S**	**S**(UPFD)		**S**																								
MedDiet			**S**					**S**	**S**		**S**		**S**								**I**			**S**	**S**						
KHEI				**S**																											
Nutritional quality					**S**(UPFD)		**S**					**I**															**S**				
AHA score						**S**																									
FSAm-NPS score																		**S**						**S**							
Qnut																										**I**					
DQnut																										**S**					
ADG																			**D**												
Total protein intake										**S**																					
Animal protein intake										**S**																					
Plant protein intake										**S**																					
Plant protein diversity										**S**																					
PANDiet score										**S**				**S**																	
PDI										**S**																					
uPDI										**S**																					
hPDI										**S**																					
DASH											**S**																				
PNNS-GS														**S**				**S**					**S**								
NCD-promoting															**S**																
															**I**(Na)																
NCD-protecting															**S**																
KIDMED																**S**															
DGI																	**S**														
GDQS																				**S**											
DGAI																						**S**									
FQS																													**S**		
YHEI-TwR-90																														**S**	

S: synergic assocation; D: discordant association; I: inconclusive association.

Abbreviations: ADG, Australian Dietary Guidelines; AHA, American Heart Association; DASH, Dietary Approaches to Stop Hypertension; DGAI, Dietary Guidelines for Americans Adherence Index; DGI, Dietary Guideline Index; DQnut, disqualifying/discouraged/negative nutrients; ED, energy density; FQS, Food Quality Score; FSAm-NPS score, Food Standards Agency Nutrient Profiling System; GDQS, Global Diet Quality Score; HEI, Healthy Eating Index; hPDI, healthful PDI; KHEI, Korean Healthy Eating Index; KIDMED, Mediterranean Diet Quality Index in children and adolescents; MedDiet, Mediterranean Diet adherence; NCD-promoting, nutrients promoting the risk for noncommunicable diseases; NCD-protecting, nutrients protecting from the risk for noncommunicable diseases; NRF, Nutrient Rich Foods; Qnut, Qualifying/encouraged/positive nutrients; PANDiet, Probability of Adequate Nutrient Intake; PDI, plant-based diet index; PNNS-GS, Programme National Nutrition Santé—Guidelines Score; uPDI, unhealthful PDI; UPFD ultra-processed foods and drink; YHEI-TwR-90, Youth Healthy Eating Index–Taiwan Revised; Na, sodium.

**Table 3.** Pattern of Identified Associations Between NOVA and Dietary Quality (“Healthfulness”) Indicators: Food Level

**Dietary quality (“healthfulness”) indicators**	Braesco et al, 2022^a^^54^	Romero Ferreiro et al, 2021^25^	Abreu and Martins, 2023^42^	Hässig et al, 2023^33^	Phulkerd et al, 2023^94^	Cooper et al, 2017^57^	Valenzuela et al, 2022^107^	Baldridge et al, 2019^45^	Phulkerd et al, 2023^95^	Heras-Delgado et al, 2023^62^	Bleiweiss-Sande et al, 2019^52^	Dickie et al, 2022^66^	Rodrigues et al, 2016^99^	Fardet et al, 2017^69^	Siqueira et al, 2021^31^	Aceves-Martins et al, 2022^43^	Angelino et al, 2023^44^	Mendoza-Velázquez et al, 2022^90^	Barrett et al, 2023^46^	Martinez-Perez and Arroyo-Izaga, 2021^88^	Mendes et al, 2021^89^	Morales et al, 2020^92^	Vergeer et al, 2019^112^	Pulker et al, 2018^96^	Gupta et al, 2019^75^	Vellinga et al, 2022^111^	
Nutri-Score	**S**	**S**	**S**	**S**			**I**			**I**		**Slight**					**S**					**I**					
NRF	**I**														**S**	**S**		**S**							**S**		
SAIN-LIM	**S**																										
LIM														**S**													
NDS														**S**													
ED	**I**										**I**				**S**			**S**							**S**	**S(**UPF)	**N**(UPD)
MTL; total fat			**D**																								
MTL; total sugar/salt			**S**																								
TDOH					**Poor**																						
WHO SEA					**Poor**																						
HCL					**Poor**																						
HSR						**I**		**I**				**Slight**							**Fair**					**S**			
CFoP							**I**																				
Nutritional quality																							**I**			**S**(UPF)	**S**(UPD)
WHO SEA									**I**(Fat, SFA, sugars)																		
									**S**(Sodium)																		
FSAm-NPS score										**I**																	
Qnut											**I**																
DQnut											**S**																
Chilean NPM												**Slight**															
WHO-Euro NPM												**Fair**															
PAHO												**Moderate**									**S**						
ADG												**Slight**															
UK/Ofcom													All:** fair**														
													With claims:** slight**														
													Without claims:** moderate**														
Nutrinform battery																	**I**										
AECOSAN																				**Moderate**							
UK NPM																				**Moderate**							

S: synergic association; D: discordant association; I: inconclusive association; N: neutral association.

^a^set of marketed foods were considered from the work of Braesco et al, 2022^54^.

Abbreviations: ADG, Australian Dietary Guidelines; AECOSAN, Spanish Agency for Consumption, Food Safety, and Nutrition; CFoP, Chilean front-of-package (FoP) food warning label; DQnut, disqualifying/discouraged/negative nutrients; FSAm-NPS score, Food Standard Agency Nutrient Profiling System; ED, energy density; HCL, Healthier Choice Logo; HSR, Health Star Rating System; LIM, score of nutrients to be limited; MTL, Multiple Traffic Lights; NDS, Nutrient Density Score; NPM, Nutrient Profile Model; NRF, Nutrient Rich Foods; Qnut, qualifying/encouraged/positive nutrients; PAHO, Pan American Health Organization; SAIN, score of nutritional adequacy of individual foods; SFA, saturated fatty acid; TDOH, Department of Health in Thailand nutrient profiling system; WHO SEA, World Health Organization South-East Asia Region nutrient profiling system; UK/Ofcom, UK/Ofcom nutrient profile; UPF, ultra-processed foods; UPD, ultra-processed drinks.

With regard to the dietary or nutritional quality indicators, the mainly food-component–based Healthy Eating Index (HEI) score was found to be synergistic with NOVA in all studies applying it (7/7).^51^^,^^74^^,^^79^^,^^81^^,^^82^^,^^100^^,^^104^ In the case of the different versions of the Nutrient Rich Foods (NRF) index, 5 studies found it synergistic^31^^,^^43^^,^^74^^,^^75^^,^^90^ with NOVA and 1 study revealed no association.^54^ The Nutri-Score was compared with NOVA in 9 studies, out of which 5 found synergy,^25^^,^^33^^,^^42^^,^^44^^,^^54^ 3 were inconclusive,^62^^,^^92^^,^^107^ and 1 estimated a “slight” level of agreement.^66^ Energy density was identified in inverse associations (interpreted as synergy in this study) with the percentage of UPF intake in 7 studies^31^^,^^63^^,^^74^^,^^75^^,^^90^^,^^103^^,^^111^ out of 9 studies. In 3 studies, no associations were found,^52^^,^^54^ of which 1 study analyzed drinks separately.^111^ Studies analyzing the association between NOVA and the nutrient profile-based Health Star Rating System (HSR) showed a mixed picture: 1 identified synergy,^96^ 1 found “fair” agreement,^46^ while 2 were inconclusive.^45^^,^^57^ Of 9 studies comparing the Mediterranean diet quality index with NOVA, 8 revealed a synergistic relationship^53^^,^^60^^,^^67^^,^^71^^,^^86^^,^^87^^,^^100^^,^^101^ while 1 was not significant.^85^ Last, among indicators analyzed at least 3 times, the Programme National Nutrition Santé–Guidelines Score (PNNS-GS) was found to be synergistic with NOVA in 3 of 3 studies.^77^^,^^80^^,^^98^

#### NOVA System’s Relation to Environmental Pressure Indicators

In contrast to healthfulness indicators, among the associations between the NOVA system and environmental pressure indicators identified by the reviewed studies (overall: *n* = 67), a mixed picture was shown (Table 4).^29^^,^^43^^,^^50^^,^^59^^,^^70–72^^,^^80^^,^^111^ The majority of them were based on diet-level assessment (*n* = 59),^29^^,^^59^^,^^70–72^^,^^80^ of which 24 showed a synergistic relationship^29^^,^^50^^,^^70–72^^,^^80^; 19 discordant^29^^,^^71^^,^^80^; and 16 showed a not significant, neutral, or inconclusive relationship.^29^^,^^70–72^^,^^80^ On the food level, 2 synergistic,^50^^,^^111^ 5 discordant,^43^^,^^50^^,^^111^ and 1 nonsignificant associations^111^ were described.

**Table 4.** Pattern of Identified Associations Between NOVA and Environmental Pressure Indicators

	Diet level										Food level			
Environmental pressure indicators	Gallegos-Riofrío et al, 2021^70^	García et al, 2023^71^	da Silva et al, 2021^59^	Kesse-Guyot et al, 2023^80^		Vellinga et al, 2023^29^			**Garzillo et al, 2022^72^**		Vellinga et al, 2022^111^		Berardy et al, 2020^50^	Aceves-Martins et al., 2022^43^
Parcel size	**N**													
Agro-diversity	**S**													
GHGE/GWP		**S**	**S**	**S**(Crude)	**I**(EA)	**D**(UPF)	**S**(UDP)	**S**(UPFD)	**S**(Crude)	**I**(Adj)	**S**(UPF)	**N**(UPD)	**S**	**D**
Blue water use/water consumption		**D**	**S**	**D**(Crude)	**D**(EA)	**D**(UPF)	**S**(UDP)	**D**(UPFD)	**S**(Crude)	**S**(Adj)	**D**(UPF)	**D**(UPD)	**D**	
Land use		**I**		**S**(Crude)	**I**(EA)	**D**(UPF)	**N**(UPD)	**D**(UPFD)					**D**	
Energy use		**S**		**S**(Crude)	**D**(EA)									
Ecological footprint			**S**											
Acidification				**S**(Crude)	**I**(EA)	**D**(UPF)	**S**(UDP)	**D**(UPFD)						
Resource use, minerals, and metals				**I**(Crude)	**D**(EA)									
Eutrophication, freshwater				**I**(Crude)	**D**(EA)	**D**(UPF)	**S**(UDP)	**S**(UPFD)						
Eutrophication, marine				**I**(Crude)	**D**(EA)	**D**(UPF)	**S**(UDP)	**D**(UPFD)						
Eutrophication, terrestrial				**S**(Crude)	**I**(EA)									
Photochemical ozone formation				**I**(Crude)	**D**(EA)									
Ozone depletion				**I**(Crude)	**I**(EA)									
Particulate matter				**S**(Crude)	**I**(EA)									
Ionizing radiation				**S**(Crude)	**I**(EA)									
EF score				**S**(Crude)	**D**(EA)									

S: synergic association; D: discordant association; I: inconclusive association; N: neutral association.

Abbreviations: Adj, adjusted model; Crude, unadjusted model; EA, energy intake–adjusted model; GHGE, greenhouse gas emissions; GWP, global warming potentional; UPD, ultra-processed drinks; UPF, ultra-processed food; UPFD, ultra-processed foods and drinks.

In the case of environmental pressure indicator subcategories, the patterns are still mixed. In the case of GHGEs (*n* = 13),^29^^,^^43^^,^^50^^,^^59^^,^^71^^,^^72^^,^^80^^,^^111^ the majority of identified relationships were synergistic (*n* = 8),^29^^,^^50^^,^^59^^,^^71^^,^^72^^,^^80^^,^^111^ while 2 discordant^29^^,^^43^ and 3 nonsignificant^72^^,^^80^^,^^111^ relationships were found. In contrast, the blue water footprint (or water consumption) was linked to NOVA classification in a discordant relationship in the majority of cases (12/8),^29^^,^^50^^,^^71^^,^^80^^,^^111^ with 4 synergist associations^29^^,^^59^^,^^72^ found. Land use also showed a rather discordant pattern: there were 3 discordant relationships found,^29^^,^^50^ in addition to 1 synergistic,^80^ 2 nonsignificant,^71^^,^^80^ and 1 neutral relationship.^29^ Further environmental indicators tested more than 3 times also showed diverse results: energy use (2 synergistic^71^^,^^80^ and 1 discordant^80^), acidification (2 synergistic,^29^^,^^80^ 2 discordant,^29^ and 1 not significant^80^), fresh water eutrophication (2 synergistic,^29^ 2 discordant,^29^^,^^80^ and 1 not significant^80^), and marine eutrophication (1 synergistic,^29^ 3 discordant,^29^^,^^80^ and 1 not significant^80^).

#### NOVA System’s Relation to Economic Affordability Indicators

Overall, including both diet- and food-level assessments, the majority of identified associations from the reviewed studies showed a discordant relationship between NOVA classification and economic affordability metrics (4 synergistic,^31^^,^^83^^,^^111^ 10 discordant,^31^^,^^43^^,^^74–76^^,^^89^^,^^90^^,^^108^^,^^111^ and 2 not significant^31^^,^^90^) (Table 5).^31^^,^^43^^,^^74–76^^,^^83^^,^^89^^,^^90^^,^^108^^,^^111^ The most often analyzed indicator, the energy-adjusted food price/diet cost, was proven discordant (in 8/7 cases)^31^^,^^43^^,^^74–76^^,^^90^^,^^108^ in the majority; diet food price/cost were found to be lower for G4 and higher for G1 in proportion, except for 1 study utilizing diet optimization.^83^ In their diet-optimization study, Maia et al^83^ found that diets with high nutrition values and G4 foods are cheaper in optimized diets where food price as a variable was not controlled for. In addition, Vellinga et al^111^ separately analyzed ultra-processed drinks (UPDs) and they were found to be more expensive (compared with MPDs). Furthermore, Siqueira et al^31^ also found UPFs to be cheaper; however, when assessing the nutrient-to-cost ratio, the relationship was synergistic with the NOVA system.

**Table 5.** Pattern of Identified Associations Between NOVA and Economic Affordability Indicators

	Diet level							Food level			
Economic affordability indicators	Gupta et al, 2021^74^	Vellinga et al, 2022^111^		Aceves-Martins et al, 2022^43^	Hallinan et al, 2021^76^	Maia et al, 2022^83^	Vandevijvere et al, 2020^108^	Siqueira et al, 2021^31^	Mendoza-Velázquez et al, 2022^90^	Mendes et al, 2021^89^	Gupta et al, 2019^75^
Diet cost/food price WA		**D**(UPF)	**S**(UPD)					**I**			
Diet cost/food price EA	**D**			**D**	**D**	**S**	**D**	**D**	**D**		**D**
Diet cost/food price WA									**I**	**D**	
Food expenditure	**D**										
NRF/price WA								**S**			
NRF/price EA								**S**			

S: synergic association; D: discordant association; I: inconclusive association; N: neutral association.

Abbreviations: EA, energy content–adjusted variables; NRF, Nutrient Rich Foods; UPD, ultra-processed drinks; UPF, ultra-processed food; WA, weight-adjusted variables.

#### NOVA System’s Relation to Other Processing Classification Systems

Overall, as presented in Table 6,^33^^,^^52^^,^^73^^,^^86^^,^^87^ the association identified from the reviewed studies regarding other processing classification systems, “fair” was the agreement level with the Australian Dietary Guidelines’ (ADG’s) discretionary food classification^73^; “moderate” was the highest agreement with the International Food Information Council (IFIC)^52^^,^^86^;“fair” and “poor” were the agreement levels with University of North Carolina (UNC) system^52^^,^^86^; and “poor” was the agreement level with the International Agency for Research on Cancer’s (IARC’s) system.^86^ Martinez-Perez et al^87^ estimated “fair” agreement with the NOVA system in their Semi-Quantitative Highly Processed Foods (sQ-HPF) questionnaire validation study. In addition, Hässig et al^33^ found a synergistic relationship between the consumers’ perception of the degree of processing and the NOVA system’s classification.

**Table 6.** Pattern of Identified Associations Between NOVA and Other Food Processing-Level Classification Systems

	Diet level		Food level		
Other food processing-level classification systems	Grech et al, 2022^73^	Martinez-Perez et al, 2022^87^	Martínez-Perez et al, 2021^86^	Hässig et al, 2023^33^	Bleiweiss-Sande et al, 2019^52^
IARC			**Poor**		
IFIC			**Fair**		**Moderate**
UNC			**Poor**		**Moderate**
perceived DP				**S**	
ADG-DF	**Fair**				
sQ-HPF		**Fair**			

Abbreviations: ADG-DF, Australian Dietary Guidelines, Discretionary Food; DP, degree of processing; IARC, International Agency for Research on Cancer; IFIC, International Food Information Council; sQ-HPF, semi-quantitative highly processed foods; UNC, University of North Carolina.

S: synergic association.

#### NOVA System’s Relation to Other Indicators Fits With the Sustainability Concept

Although indicators in this category are different from each other in their target (ie, other than previously mentioned indicators and rarely applied indicators), they were found to be almost homogenously synergistic, except for the Dietary Inflammatory Index (DII), which was found to be synergistic in 1 study^91^ while not significantly connected to NOVA in another^85^ (Table 7).^33^^,^^47^^,^^51^^,^^61^^,^^65^^,^^69^^,^^85^^,^^91^^,^^92^ Traditional foods (eating) was assessed in 2 studies; both found a synergistic relationship to NOVA.^47^^,^^51^ The other indicators were all synergistic as assessed on a single occasion in the reviewed studies (satiety, glycemic impact,^69^ perceived healthfulness,^33^ food addiction,^61^ Potential Renal Acid Load [PRAL], Net Acid Production [NEAP],^65^ hydroxymethylfurfural [HML], acrylamide^92^).

**Table 7.** Pattern of Identified Associations Between NOVA and Rarely Applied Sustainable Diet Indicators

	Diet level					Food level			
Other sustainable diet indicators	Batal et al, 2018^47^	Hässig et al, 2023^33^	Detopoulou et al, 2023^65^	Blanchet et al, 2020^51^	Vicente et al, 2023^85^	Fardet et al, 2017^69^	Mignogna et al, 2022^91^	Delgado-Rodríguez et al, 2023^61^	Morales et al, 2020^92^
Fullness factor (satiety)						**S**			
Glycemic impact						**S**			
Traditional foods	**S**			**S**					
Perceived healthfulness		**S**							
DII					**I**		**S**		
Food addiction								**S**	
PRAL			**S**						
NEAP			**S**						
HMF									**S**
Acrylamide									**S**

S: synergic association; I: inconclusive association.

Abbreviations: DII, Dietary Inﬂammatory Index; HMF, hydroxymethylfurfural; NEAP, net acid production; PRAL, Potential Renal Acid Load.

### Limitations of Using NOVA Mentioned by the Authors in the Reviewed Studies—Categories Based on Qualitative Coding

Based on qualitative analysis (Table S3)^29^^,^^31^^,^^42^^,^^43^^,^^46^^,^^47^^,^^53^^,^^55^^,^^56^^,^^59^^,^^60^^,^^61^^,^^65^^,^^66^^,^^71^^,^^73^^,^^74^^,^^78^^,^^79^^,^^81^^,^^84^^,^^86^^,^^98^^,^^100–105^^,^^108^^,^^111^) the main themes of limitations mentioned (in 31 studies out of 77) were “inherent problems of classification” (*n* = 17),^29^^,^^42^^,^^43^^,^^46^^,^^47^^,^^61^^,^^65^^,^^66^^,^^73^^,^^74^^,^^100–105^^,^^111^ “lack of information for NOVA classification” (*n* = 13),^42^^,^^43^^,^^47^^,^^53^^,^^55^^,^^59^^,^^73^^,^^79^^,^^84^^,^^86^^,^^102^^,^^104^^,^^108^ “problems with dietary data recording” (*n* = 9),^29^^,^^47^^,^^53^^,^^60^^,^^71^^,^^78^^,^^81^^,^^84^^,^^100^ “food group–specific classification problems” (*n* = 7),^65^^,^^66^^,^^71^^,^^73^^,^^98^^,^^102^ “‘blindness’ for foods high in fat, salt and/or sugar” (*n* = 2),^43^^,^^66^ “fewer studies assessing G1 and G3 compared to G4” (*n* = 2),^47^^,^^102^ “dietary level of analysis” (*n* = 1),^31^ and “cross-cultural extrapolation” (*n* = 1).^56^ With regard to “inherent problems of classification,” the inadequacy of definition and the need for classification refinement^29^^,^^43^^,^^46^^,^^61^^,^^66^^,^^74^^,^^101^^,^^102^^,^^111^ were mentioned besides the possible ambiguity of investigators and, accordingly, the need for double-coding,^104^ as well as that NOVA fails to add considerable value to nutritional profile assessments. In the case of “lack of information for NOVA classification,” the lack of information for proper classification in general,^53^^,^^55^^,^^79^ from food labels^42^^,^^102^ and ingredients,^59^ especially for mixed dishes, homemade meals, and ready-to-eat foods,^43^^,^^104^^,^^108^ and food-composition tables for matching were limitations.^47^^,^^73^^,^^86^ With regard to “problems with dietary data recording,” the main issue was the lack of validated questionnaires,^60^^,^^71^^,^^78^^,^^84^ posterior coding of foods,^47^ and the lack of UPFs incorporated in the questionnaires.^53^^,^^100^ The “food group–specific classification problems” occurred in the limitations emphasizing breads, yogurts, dairies, and processed meats^66^^,^^71^^,^^73^ besides other food groups,^71^ as well as the increased consumption of plant-based foods^102^ as problematic for NOVA classification. Although not the purpose of NOVA, the “‘blindness’ for foods high in fat, salt, and/or sugar” was mentioned twice^43^^,^^66^ as well as the “fewer studies assessing G1 and G3 compared to G4.”^47^^,^^102^ The dietary level of analysis (mentioning food-level analysis as disadvantage compared to dietary level^31^) and the issues of “cross-cultural extrapolation”^56^ were mentioned once (see further details and references in Table S3).

### Key Findings Related to NOVA Classification—Categories Based on Qualitative Coding

As further detailed and referenced in Table S4,^9^^,^^25^^,^^29^^,^^31^^,^^33^^,^^42–113^ related to the NOVA system, the key conclusions and key findings of the reviewed studies were categorized as follows: “NOVA’s alignment with other SDIs” (*n* = 53),^29^^,^^33^^,^^42^^,^^44^^,^^45^^,^^49^^,^^50–53^^,^^55–57^^,^^60–64^^,^^66–73^^,^^75–77^^,^^79–83^^,^^85^^,^^86^^,^^88–92^^,^^96^^,^^98–107^^,^^108^^,^^111^^,^^112^ “Policy implications”(*n* = 28),^9^^,^^25^^,^^42^^,^^46^^,^^48^^,^^49^^,^^51^^,^^52^^,^^56^^,^^64^^,^^68^^,^^74^^,^^75^^,^^80^^,^^84^^,^^89^^,^^90^^,^^92^^,^^93^^,^^95^^,^^97^^,^^99^^,^^105^^,^^106^^,^^108^^,^^109^^,^^110^^,^^113^ “Sustainable dietary implications related to NOVA classification” (*n* = 20),^29^^,^^31^^,^^43^^,^^47^^,^^53^^,^^55^^,^^59^^,^^60^^,^^65^^,^^68^^,^^71^^,^^76^^,^^78^^,^^79^^,^^80^^,^^83^^,^^84^^,^^97^^,^^101^^,^^102^ “Recommendation to use NOVA with other SDI(s)” (*n* = 5),^58^^,^^77^^,^^94^^,^^104^^,^^112^ “Recommendation for the application of NOVA” (*n* = 4),^61^^,^^66^^,^^87^^,^^104^ “Critics of NOVA” (*n* = 3),^54^^,^^111^^,^^112^ and “Supporting argument on NOVA” (*n* = 2).^58^^,^^94^ With regard to the most common main categories, the subcategories of “NOVA’s alignment with other SDIs” are, by the identified associations of NOVA with other SDIs, as follows: positive alignments in the majority (*n* = 28),^29^^,^^33^^,^^44^^,^^49^^,^^51–53^^,^^55^^,^^56^^,^^60^^,^^61^^,^^63^^,^^67^^,^^69^^,^^70^^,^^73^^,^^77^^,^^81^^,^^82^^,^^83^^,^^86^^,^^91^^,^^98^^,^^100^^,^^101^^,^^103^^,^^104^^,^^105^ inconclusive overall conclusion with NOVA’s alignment with other SDIs based on all results (*n* = 22),^37^^,^^42^^,^^45^^,^^50^^,^^52^^,^^57^^,^^62^^,^^64^^,^^66^^,^^71^^,^^72^^,^^75^^,^^76^^,^^79^^,^^80^^,^^88^^,^^90^^,^^92^^,^^96^^,^^102^^,^^111^^,^^112^ and negative alignments concluded (*n* = 4).^85^^,^^89^^,^^107^^,^^108^ With regard to “Policy implications,” the following issues were identified: NOVA was recommended as a label element (with Nutri-Score),^25^^,^^42^ completion of NOVA with nutritional quality analysis was encouraged,^64^^,^^95^ and, in the case of plant-based alternative products (PBAPs), hybrid assessment (NOVA and Nutri-Score) would be optimal.^62^ However, that can lead to policy confusion due to the discordance of the 2 systems in certain product categories.^46^ In addition, different food policy and public health interventions were mentioned, as follows: monitoring and evaluating the quality of the food market/portfolio/environment with NOVA; regulation on promotions of UPFs, especially targeting children (in contrast to fresh and MPFs) ^95^^,^^99^^,^^110^; manufacturers should consider the reformulation of recipes to avoid the UPF category^42^^,^^92^; intervention to limit UPF intake in order to achieve better public health and a more sustainable food system^74^^,^^80^^,^^108^; to apply nutrient profiling alongside NOVA to avoid micronutrient deficiency^62^; minimum wage policy as intervention may not change the habit of consuming UPFs^93^; to consider replacement foods in case of limiting UPF intake^68^; traditional-food eaters more likely make food at home and avoid purchasing UPFs^51^; urban populations lack access to gardens, which may cause more intake of UPFs^113^; affordability of MPFs should be promoted^108^; FBDGs should address the economic problem of UPFs being cheaper^75^; and manufacturers have low motivation to launch fresh foods and MPFs.^105^ In addition, it was noted that the substitution of the fortified foods falling into the UPF category could be an obstacle to achieving nutritional adequacy (eg, in the case of grains, the risk of iodine, folate, thiamin, and iron deficiency)^68^; however, it was also pointed out that fortification only resolves single nutrient problems, yet it can disturb the sustainability of the indigenous food system.^113^

“Sustainable dietary implications related to NOVA classification” was the third most common main category and consisted of the following topics: The lowered intake of UPFs would improve the quality of diet^55^^,^^78^^,^^79^^,^^84^ and, in parallel, could contribute to better planetary health^29^^,^^59^; however, these results vary in terms of environmental pressure categories (eg, lower UPF intake would cause lower GHGEs and energy use but increased water consumption).^71^ Moreover, while the reduction in UPD intake would serve both human and planetary health, in the case of UPFs, besides better health outcomes, a varying effect on the environment is estimated.^29^ Higher UPF intake leads to higher energy intake, which negatively affects the environmental pressure of diets.^59^^,^^80^ Increasing the plant-based protein intake in the future is key to sustainability; however, plant-based meat alternatives are often identified as UPFs that should not replace fresh and minimally processed vegetables.^97^^,^^102^ Better Mediterranean diet adherence can cause lower UPF intake,^60^^,^^71^^,^^101^ and traditional-food eaters have a higher intake of MPFs, thus contributing to better diet quality.^47^ The lower intake of UPFs may lead to more expensive diets^43^^,^^74^^,^^82^; however, due to the high variety of foods, there is a feasible solution for affordable green and healthy diets.^43^^,^^83^ Notably, the exclusion of UPFs from the diet may lead to nutritional inadequacy—for example, micronutrient deficiency—so both MPFs and UPFs are necessary for healthy diets.^76^^,^^68^ Finally, UPFs are high in phosphate, which creates a more acidic environment in the human metabolism.^65^

In the “Recommendation to use NOVA with other SDI(s)” category, it was mentioned that the NOVA system is not interchangeable with the HEI (FBDG adherence).^104^ NOVA’s completion with nutrient-profiling systems was also suggested,^77^^,^^94^ moreover noting that the nutrient-profiling systems are more adequate since nutrient densities vary at all processing levels of foods.^112^ The Siga approach was mentioned as not being a substitute for NOVA but a more objective tool for retailers and agri-food industry stakeholders.^58^ The thematic category “Recommendation for the application of NOVA” included comments on the necessity to qualify the extent of recommendations for G3 intake,^61^ to apply double-coding, and to cross-validate NOVA classification,^104^ so that NOVA could be a benchmark method for the evaluation of healthy foods, with technical adjustment.^66^ Finally, it was noted that the choice of (food-processing level) classification system has a considerable impact on research outcomes.^87^ With regard to “Critics of NOVA,” it was pointed out that NOVA does not support the robust and functional classification of foods^54^; is not adequate to identify healthfulness, cost, and environmental pressure “win-wins”^111^; and puts too great a focus on technological aspects instead of added sugars, salt, and additives.^112^ Finally, in “Supporting argument on NOVA,” it was mentioned that NOVA is capable of capturing the interaction of bioactive compounds, and the food matrix effects in contrast to other nutrient profiling systems,^94^ and that it is necessary and sufficient for both consumers and research purposes^58^ (see further details and references in Table S4).

## DISCUSSION

### NOVA and Other Sustainable Diet Indicators in a Wider Context

#### “Healthfulness”/Dietary Quality Indicators: What Is NOVA’s Role?

Even though not in complete agreement, NOVA was found to be synergistic in the majority of the identified associations with other dietary quality (healthfulness) indicators, regardless of whether the indicator includes nutrients, food, or mixed components or whether the analyses were conducted on the food or diet level. On the other hand, NOVA appears to be more synergistic on diet-level assessments compared with food-level assessments, which suggests that it fits better with more complex dietary quality indicators while shows dissonances with nutrient-level assessment. Focusing on the discordant relations, Estell et al^68^ and Hallinan et al^76^ found that, in case of the complete exclusion of UPFs, a healthy diet is not feasible due to micronutrient deficiencies. These results suggest that, in these terms, a certain amount of UPFs or proper substitutions are necessary to have a healthy diet in terms of nutrient profile. In addition, in the reviewed studies, among healthfulness indicators, NOVA shows great synergy with Mediterranean diet adherence and the FBDG-based HEI, due to their food-based components and recommendations for fresh, minimally processed foods (Table 2). It is important to note that, despite the statistical association supporting the positive relationship of NOVA and other dietary quality indicators, in most cases, there was a set of foods, categorized as “healthy” by the other dietary quality indicator and UPFs (ie, “unhealthy”) by NOVA and vice versa.

While there is a moderate level of evidence that links UPF intake (defined by NOVA G4) and all-cause mortality including common NCDs,^17^^,^^18^ in the same umbrella review very low evidence was found for the association of UPFs and nutrient intake; however, in population studies it was observed that higher UPF intake leads to unfavorable nutrient intake profile.^6^ Barbaresko et al^18^ point out that it is unclear if UPF intakes themselves cause poor health outcomes or if they are merely markers of poor dietary outcomes. Similarly, FoodDrinkEurope^12^ stated that the level of processing is inadequate to measure healthfulness since it does not define nutritional composition. In contrast, according to Fardet et al,^22^ there is no direct evidence of the effect of nutrition on the human body, since humans consume complex food matrices and not isolated nutrients. The complex factors of food matrices could affect glycemic impact, satiety, microbiome modification, neurobiological pathways, etc.^30^^,^^114^ In the reviewed articles, Bonaccio et al^53^ found that NOVA, independent of nutrient composition, was associated with poor health outcomes, while according to Romero Ferreiro et al,^25^ the level of processing may be greater than the nutrient content in influencing health. Moreover, WHO names UPFs as one of the commercial determinants of health besides alcohol, tobacco, and fossil fuels, because of their large contribution to NCDs.^19^ Consequently, these results suggest NOVA’s independent role from nutrient intake on health outcomes.

This review also supported the statement that NOVA and nutrient-profile systems are complementary but not interchangeable^20^^,^^25^^,^^26^^,^^42^^,^^77^ and, applied together, they provide a comprehensive picture of the healthfulness of foods and diets and hybrid labeling could inform consumers of these health implications. According to Srour et al,^26^ in a consumer study testing Nutri-Score V2.0 (incorporating UPFs mark with Nutri-Score on the food label), in the case of discordant foods (eg, UPFs and Nutri-Score A) consumers preferred the non-UPF foods with the best available Nutri-Score rather than Nutri-Score A or B with the UPF mark.

#### NOVA and the Environmental Pressure of Diets

In the case of environmental pressure indicators, the picture is the most complicated, which, in part, resembles the problematic trade-offs of sustainable nutrition in general. The effort to lower environmental impact can lead to micronutrient deficiencies,^5^ diets adjusted to healthy dietary recommendations may lower GHGE and land use, but not water consumption,^3^^,^^115^ yet alignment could be reached, especially if controlled for blue water consumption.^32^^,^^116^ According to the patterns of association of this review, trends for NOVA–environmental pressure relations could not be concluded, which appear if the direction of association is dependent on the context, such as population, system boundaries, study design, model parameters, other indicators, subcategory of environmental pressure indicators, etc. However, the association pattern showed NOVA's synergy with GHGEs and discordance with blue water consumption, which is similar to other healthy diet indicators applied in sustainable nutrition research.^3^^,^^115^ Kesse-Guyot et al^80^ and Berardy et al^50^ pointed out that the production stage of the food chain plays a bigger part in the environmental pressure values of French representative diets and among North American Adventists, while NOVA classification–based higher UPF intakes and related environmental pressure had a greater impact on the postharvest stages, which amounted to significant energy demand besides water use and GHGEs.^80^ Furthermore, Kesse-Guyot et al^80^ concluded that the higher intake of UPFs leads to higher energy intake, which causes elevated environmental pressure; thus, limiting UPF intake would benefit both the public and planetary health.^80^ Moreover, according to Fardet and Rock,^117^ the combination of low-cost ingredients and increased worldwide consumption (of UPFs) could increase the environmental pressure of food production. This is especially important, because, as mentioned, UPFs are linked to higher dietary energy intake,^18^ which can lead to higher environmental pressure.^80^ For example, the dietary contribution of UPFs increased in parallel with environmental pressure in the Brazilian population in the last 30 years.^59^ These results suggest that, in addition to a mixed association on the food level, complex dietary and temporal analysis shows that UPF consumption drives growing environmental pressure through energy intake as a factor. This conclusion was also supported by Hendrie et al,^118^ who analyzed the effect of discretionary foods on dietary environmental pressure in an Australian population study, where they found that excessive UPF intake may be a key driver of avoidable environmental pressure of the diet.

Consequently, when applying NOVA in sustainable nutrition research or a dietary recommendation or policy intervention, to control for the environmental pressure of dietary changes seems to be a prerequisite for reaching alignment with more sustainable diets; however, with this approach, favorable solutions can be reached to achieve more sustainable diets.

#### NOVA and Economic Affordability

One of the attractive characteristics for consumers of UPFs is that they are affordable and convenient,^30^ which assumes a discordant relationship to NOVA that was supported based on the reviewed studies, regardless of food- or diet-level analyses. However, different approaches revealed that the association of affordability and level of processing could be different: Vellinga et al^111^ found that even UPFs are discordant with diet cost, UPDs are not, and they are more expensive than fresh or MPDs. Also, in a diet optimization model, there is a feasible solution to decrease UPF intake and diet cost at the same time.^83^ Moreover, when the nutrient-to-cost ratio was assessed, there was a synergy between the level of processing and economic affordability.^31^ These results point towards the question of which affordability metrics are the best indicator in the case of sustainable nutrition research integrating the level of processing as a dimension. Furthermore, these results also suggest, as concluded by Gupta et al,^75^ that economic affordability in the case of UPFs and their discordant direction towards more sustainable diets should be addressed in FBDGs. Consequently, with regard to food or health policy interventions recommending lower intake of UPFs, especially among economically vulnerable population, it is necessary to offer the substitutions that can provide feasible, sustainable, and healthy diets with the same or lower cost and decreased UPF contents. This issue has been addressed by the WHO, which recommended food taxes for foods not contributing to a healthy diet, which include UPFs and sweetened beverages, while they also recommend subsidizing foods that promote healthy diets, especially to benefit lower-income populations with their disproportionate burden of disease.^119^

#### NOVA and Other Classifications of the Level of Processing

The classification systems incorporating the level, origin, and type of processing differ in terms of defining “healthier” and “less healthy” categories; however, their overall target is to identify food that increases the risk of NCDs. In the association pattern analysis, they showed “poor” and “moderate” agreement in this aspect, among which the IFI and UNC had the greatest level of agreement with NOVA,^73^ while the lowest agreement was found with IARC.^86^ In a systematic review, Moubarac et al,^120^ compared NOVA, IARC, IFIC, National Institute of Public Health in Mexico (NIPH), and International Food Policy Research Institute (IFPRI) classification systems that incorporate food processing and found NOVA to have the best quality, besides being the most often applied system with a global scope.^86^ Moreover, NOVA has also been applied alongside traditional-food eating among First Nations populations to measure dietary quality.^121^

It should be noted that, in a consumer study, the perceived level of processing was strongly correlated with the NOVA classification system’s identification of it as well as with the perceived healthfulness, which means this aspect of food comes naturally to consumers.^33^ Moreover, the majority of European Union (EU) consumers tend to perceive UPFs as unhealthy and unsustainable,^30^ while Srour et al^26^ demonstrated that consumers could benefit from front-of-pack labeling combining the Nutri-Score with UPF label element.

These results suggest that education and food label regulation based on NOVA classification could be effective among consumers and further investigation could help plan policies that target consumer behavior.

### NOVA Is Applied in Classic (Sustainable) Nutrition Research But Not Adapted to It

The global results of this scoping review suggest that, while NOVA is applied in the classic methodological context of (sustainable) nutrition, it has not yet been adapted to it. In the reviewed articles, NOVA is applied in the same way as other sustainable nutrition indicators, although NOVA per se is different and would require special consideration for assessment. The research context analysis and key word co-occurrence (Appendix S4) showed that the reviewed set of articles are applied in a usual sustainable nutrition context with otherwise common SDIs used with NOVA: Nutri-Score, Mediterranean diet adherence, carbon and water footprint, and food prices.^4^^,^^8^ While the overwhelming majority of SDIs are reductionist, they measure a certain parameter (eg, vitamin C content or energy use) and could be described with 1 dimension or integrated value.^4^^,^^8^ NOVA provides food component–based, rather qualitative or categorical, variables that are further processed to give quantitative data (Table S1). In addition, its classification is made by expert judgment and requires as precise information as possible. In the qualitative analysis of the limitations of using NOVA (Table S3) it commonly appeared that the dietary recording methods are not validated in identifying NOVA groups (and UPFs at all), which is a common issue in all research focusing on NOVA.^18^ Moreover, data-management approach, labeling, and food-composition tables fail to provide enough information for classification, especially if done a posteriori. While 1 part of the problem originated in the inherent uncertainties of NOVA classification, such as the ambiguity and unclear definition enabling robust classification,^47^^,^^74^^,^^122^ another, greater part of the problem constitutes issues that all sustainable nutrition research encounters: the problems of matching food lists with food-composition databases, aggregation of food groups where substitution and generalization of foods are unavoidable,^122^ and the generally uncertain methods (eg, 24-hour dietary recalls and FFQs) that are based on self-report.^123^

Consequently, the standardization of study protocols using NOVA seems to be necessary,^18^^,^^29^^,^^47^^,^^100^^,^^111^ especially the data-recording and classification phases with experts using double or triple coding.^104^ Similarly, the effect on results originated in the choice of diet- or food-level analysis, food-group classification and aggregation, database compilation with SDIs, variables estimated from NOVA classification, and the NOVA groups compared in the analysis of results should be further investigated.

### Specific Problems of Food Groups

The results on the proportion of NOVA-identified UPFs in the sample show an apparent difference in food- and dietary-based approaches that could result in great differences, which need to be explored in further research. NOVA identifies the majority (>50%) of foods as UPF and thus as “unhealthy” (ie, UPFs: 25/34 of the reviewed studies assessing on food-level, in contrast with diet-level, analyses [7/30 cases]), which might demonize certain nutritionally adequate foods among consumers who perceive UPFs as unhealthy and non-UPFs as healthy.^26^^,^^30^^,^^33^ This is especially a problem in the case of foods that are identified as UPFs, yet the complete elimination of them could lead to nutrient deficiencies.^64^^,^^68^ The classification of bread was mentioned as problematic by authors (Table S3) since it is difficult to distinguish artisan and ready-to-eat breads^73^^,^^111^; they can be classified either as processed foods or UPFs but it isn't clear for experts. Dairy products are similarly problematic,^66^^,^^71^^,^^98^ especially sweetened yogurts that may fall into separate NOVA groups with no definite differences.^71^ In addition, these food groups may be fortified with key micronutrients (eg, iodine).^68^ This is a paradoxical situation that creates a public health conflict, because some experts categorized these food groups as UPFs^68^ while other FBDGs identify them as “healthy.”^73^ Moreover, several authors mentioned the problem of classifying mixed dishes in the absence of a detailed ingredients list, to distinguish artisan and ready-to-eat foods, etc.^29^^,^^43^^,^^47^^,^^71^^,^^73^^,^^104^^,^^111^ According to Aceves-Martins et al,^43^ to create a more precise classification, home-prepared foods should be separated from industrially produced foods when coding dietary data by processing category.

Another emerging problem is the classification of PBAPs, the considerable share of which are classified as UPFs (in the work of de Las Heras-Delgado et al^62^: 37%; Rizzolo-Brime et al^97^: 93.9%). This is, in part, because processing increases the environmental pressure of plant-based products.^50^ In addition, the plant protein intake is synergistic with NOVA: it is lower if UPF intake is higher.^102^ Also, NOVA was found to be synergistic with plant protein diversity and total protein intake in the same work. In addition, Rauber et al^124^ found a positive association between plant-sourced UPFs and cardiovascular risk in a US cohort study. The purpose of PBAPs is not to replace fresh fruits and vegetables and their nutrient profile but to replace a certain proportion of animal protein intake with plant protein intake.^62^ That discrepancy should be addressed in the communication to consumers in order to finding an optimal trade-off. An example could be the consideration of food-processing level in plant-based dietary quality scores, in which, according to Marchese et al,^125^ UPFs are the least represented (considered for scoring) food group in these scores. Therefore, while NOVA currently has the power to nudge innovation towards improving the nutritional quality of the products from this food group and reducing the environmental impact of their production, strong conflicts can arise in the future if the NOVA classification methodology does not adapt towards a more robust distribution of food items.

Finally, as part of the sustainable nutrition approach, a radical dietary shift (ie, extreme limitations or exclusion of UPFs in the diet) in cultural acceptability or current food consumption could be unlikely, such as in the case of shifting to vegan diets.^3^ Further nutrition research and dietary recommendations should focus on defining the healthy limits of UPF intake within the context of sustainable diets. With regard to this, Fardet and Rock^9^ estimated—based on population studies—a maximum threshold of 15% (of total energy intake) in addition to the consideration of plant- and animal-based food ratio, diet diversity, and biological and local origins of foods.

### NOVA Groups—A Need for More Data on the Effect of G1 and G3 on Sustainable and Healthy Diet Outcomes

In this review, the logic for estimating the NOVA system’s relation to other SDIs was based on the distinction of NOVA-identified UPFs and non-UPFs (interpreted as “unhealthy” and “healthy”), since there is valid evidence linking the term “UPFs” to “unhealthfulness” identified by NOVA,^16–18^ but that does not necessarily define the role of G1, and especially G3. Moreover, as mentioned in 2 studies, a limitation of an assessment based on NOVA classification is that there are not enough data on G1 and G3 to compare results.^47^^,^^102^ Scientific evidence shows that G1 (ie, unprocessed, MPFs) provide positive health effects if their intake is adequate^126^; however, this food group has varying definitions (defined as “fresh” or “unprocessed”), but FBDGs include recommendations for their intake.^14^^,^^126^ Still, the question remains whether G1 defined by NOVA provides a positive health effect, or if it simply just does not promote negative outcomes; moreover, this question in the case of G3 is even more prominent. In the reviewed articles, 41 (out of 77) showed comparable results on all 4 NOVA groups (or a subset of them, typically leaving out G2), while almost half of the studies applied the UPF/non-UPF binarity (*n* = 32), commonly those that conducted analyses on the diet level (*n* = 21), while 2 studies used both approaches.^60^^,^^79^ In this review, 44 reviewed studies showed comparable results for G1 on G3 separately; the role of G3 was commonly considered a positive counterpart to G4, while in 1 study, it was merged with G4 as a “less healthy” group,^99^ further complicating the picture. Standardizing the variables generated based on NOVA, besides harmonizing the dietary recording and classification process, could help create more comparable data. It is especially important because reduced or minimal processing could be an important factor towards a more sustainable food system.^36^ Nevertheless, further studies analyzing the link between UPF consumption and NCDs might focus on all NOVA groups to complete the understanding of the role of G1 and G3.

## LIMITATIONS

This review is not without limitations. First, the review was limited to studies published in English, potentially excluding relevant research published in other languages. Furthermore, the interpretation of the results of content analysis can be considered inherently subjective and may have been seen differently by other peers. However, to ensure transparency, we provided every text segment belonging to each identified category from the analyzed publication in the supplementary material to leave the possibility for re-interpretation for those who are interested in using or citing this review. The connection of the authors to various firms or lobby organizations via direct or indirect financing of research or otherwise was not analyzed. This fact can be a source of bias regarding results.

## CONCLUSION

In this scoping review, the NOVA system’s application in the context of sustainable nutrition research and its relationship with other SDIs was explored. Although established using a different approach, NOVA is synergistic—in the majority—with other nutrient-profiling and FBDG-based healthfulness indicators to which it can be regarded as complementary, although not interchangeable. Previous public health studies suggest the role of UPFs in the development of NCDs, which points towards the conclusion that the NOVA classification that identifies UPF intake could be an independent added value for the assessment of healthy diets and foods in the sustainable nutrition research context. The relationship between NOVA classification and environmental pressure indicators is mixed, similarly to other healthfulness indicators; however, there is variation according to the stages of the food chain assessed and model parameters applied. Consequently, studies and policy interventions applying NOVA should control for the environmental pressure effect of dietary changes. Diets higher in UPFs tends to be cheaper and more convenient for consumers compared with MPFs; thus, dietary interventions towards reducing UPF intake should control for price, especially among economically vulnerable populations.

NOVA is the most widely used system classifying the level of processing of foods that can be globally applied. The most recent reviews defined UPFs as classified by NOVA to investigate the association of dietary quality and health outcomes, which suggests the unavoidable role of NOVA in sustainable nutrition assessments. To consider the level of processing of food is an additional aspect of sustainable nutrition dimensions that could be investigated with diet optimization or other complex approaches that can handle multiple aspects. Sustainable nutrition research integrating NOVA classification should adapt the study design to it by using validated data-recording methods, standardizing classification process applying multiple coding, and improve data processing and analysis.

## Supplementary Material

nuae207_Supplementary_Data

## Data Availability

Detailed bibliometric dataset is available upon request to the authors.
