# Supplementary material for: Fifteen Years of NOVA Food-Processing Classification: “Friend or Foe” Among Sustainable Diet Indicators? A Scoping Review
Source: Nutr Rev. 2025 Jan 23;83(4):771–91. doi: 10.1093/nutrit/nuae207 (PMC11894255; doi:10.1093/nutrit/nuae207)
Supplement: nuae207_Supplementary_Data [file nuae207_supplementary_data.zip › nuae207_Supplementary_Data/Appendix_S2_Searhing_query_and_Prisma_flow_diagram.docx]

*Searching query*

"NOVA" OR "NOVA food classification" OR "NOVA-score" OR "NOVA classification" (Topic) and "application" OR "assessment" OR "evaluation" OR "analysis" OR "quantification" OR "optimization" OR "validation" OR "test" (Topic) and "diet" OR "nutrition" OR "meal" OR "food" (Topic) and Review Article or Early Access or Article (Document Types) and 2023 or 2022 or 2021 or 2020 or 2019 or 2018 or 2017 or 2016 or 2015 or 2014 or 2013 (Publication Years)

*Prisma 2020 flow diagram^1^*

**Identification of studies via databases and registers**

Records removed *before screening*:

Duplicate records removed (n = 513)

Records identified from:

Databases (n = 1612)

Web of Science (n = 471)

Scopus (n = 791)

PubMed (n = 350)

**Identification**

Records screened

(n =1065)

Records excluded

No DOI number: (n = 34)

Reports not retrieved after title and abstract screening

unrelated content (n =713)

Reports sought for retrieval

(n =1031)

**Screening**

Reports assessed for eligibility

(n =318)

Reports excluded:

not meeting with inclusion criteria (n = 208)

no full-text available (n = 8)

no English full-text (n = 25)

Studies included in review

(n = 77)

Reports of included studies

(n = 77)

**Included**

References

S1 Page MJ, McKenzie JE, Bossuyt PM, Boutron I, Hoffmann TC, Mulrow CD, et al. The PRISMA 2020 statement: an updated guideline for reporting systematic reviews. BMJ 2021;372:n71. doi: 10.1136/bmj.n71
