# Supplementary material for: Fifteen Years of NOVA Food-Processing Classification: “Friend or Foe” Among Sustainable Diet Indicators? A Scoping Review
Source: Nutr Rev. 2025 Jan 23;83(4):771–91. doi: 10.1093/nutrit/nuae207 (PMC11894255; doi:10.1093/nutrit/nuae207)
Supplement: nuae207_Supplementary_Data [file nuae207_supplementary_data.zip › nuae207_Supplementary_Data/Appendix_S4_Bibliometric_analysis.docx]

Bibliometric analysis of the reviewed articles

*Publication Channels*

Of the 77 publications^1-77^, nearly a third (n = 26) were published in two journals (Nutrients and Public Health Nutrition), 31 were published in other 12 different journals, while there were 20 publications published in journals appearing only once in the analysis (frequency = 1). As for the research areas, half of the journals (16 out of 34) were targeted at readers interested in food and nutrition sciences, 4 journals belonged to the field of public health, and 9 to clinical medicine.

*Scientific production*

In terms of scientific production, an increasing trend is observed in the examined period regarding the assessment of the NOVA classification with other sustainable diet indicators between 2017 and 2023 (Figure 1.). The number of outputs increased from 2 to 5 per year between 2017 and 2020, indicating an overall upward trend for scientific production, while there was a more notable increase in the number of outputs from 2020, when the number of publications tripled, and the scientific output on the topic has remained consistent since then, ranging between 15 and 20 annually. The average total citation peaked at 2018, however, there were considerable fewer publication in 2018 and before (n = 8), then after (n = 69) pointing towards fundamental works, two of the top 3 most cited work was published in 2018 (Gupta et al., 2019^34^: total citations = 89; Lavigne-Robichaud et al., 2018^42^: total citation = 70; Batal et al., 2018^6^: total citations = 60) (Figure 2.). On the other hand, the average citation per article shows a more equally distributed picture (Figure 3.)


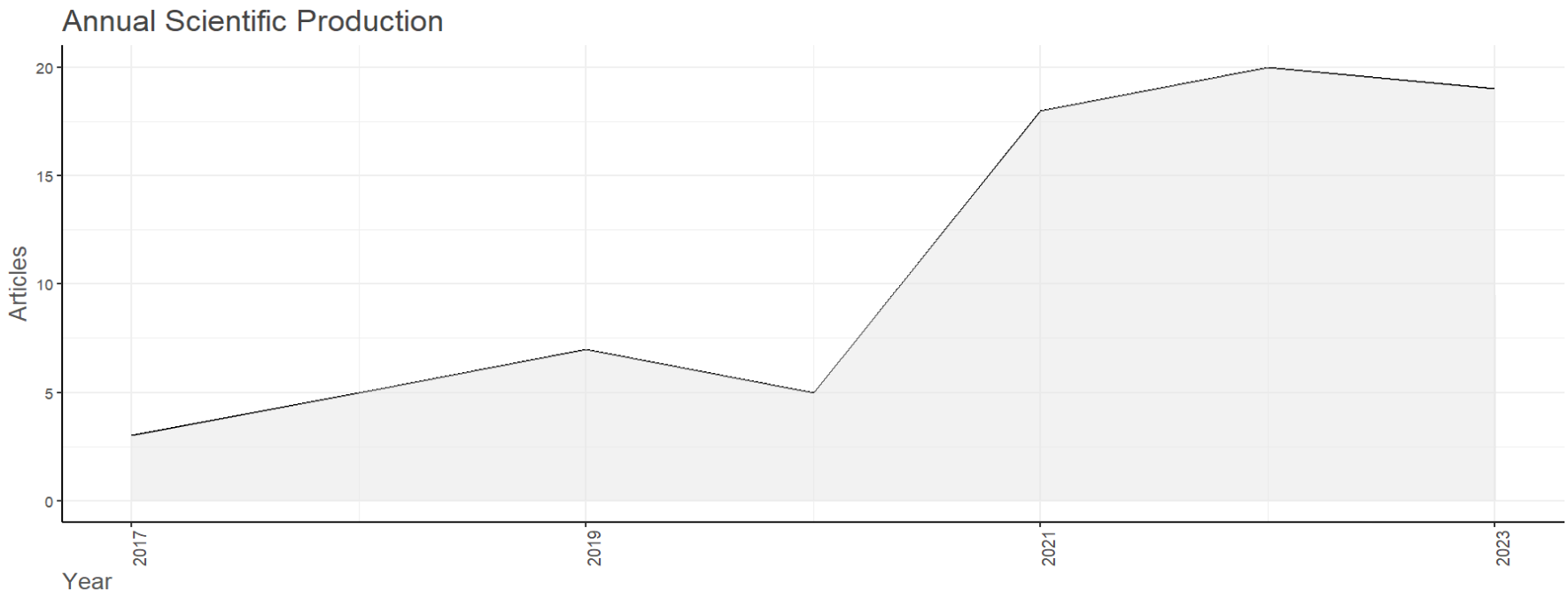


Figure 1.: Scientific output / per year among the reviewed studies (n = 77)


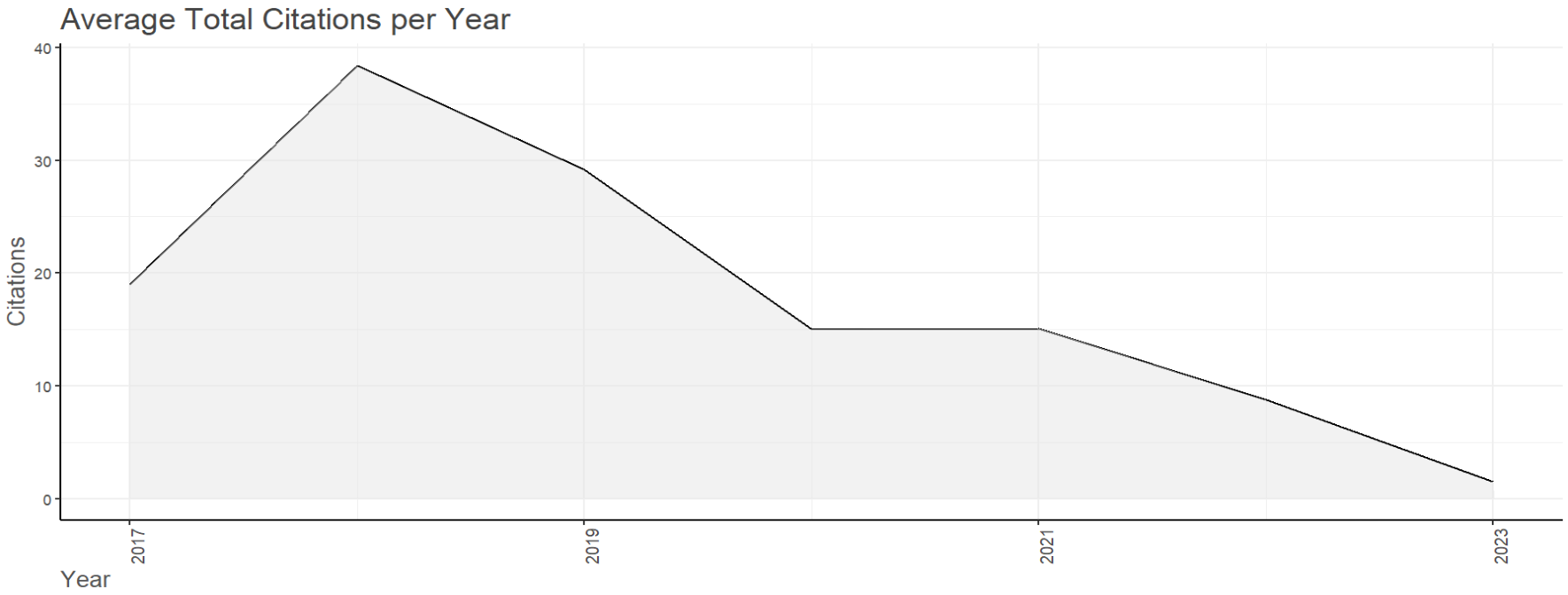


Figure 2.: Average total citations / year of the reviewed studies (n = 77)


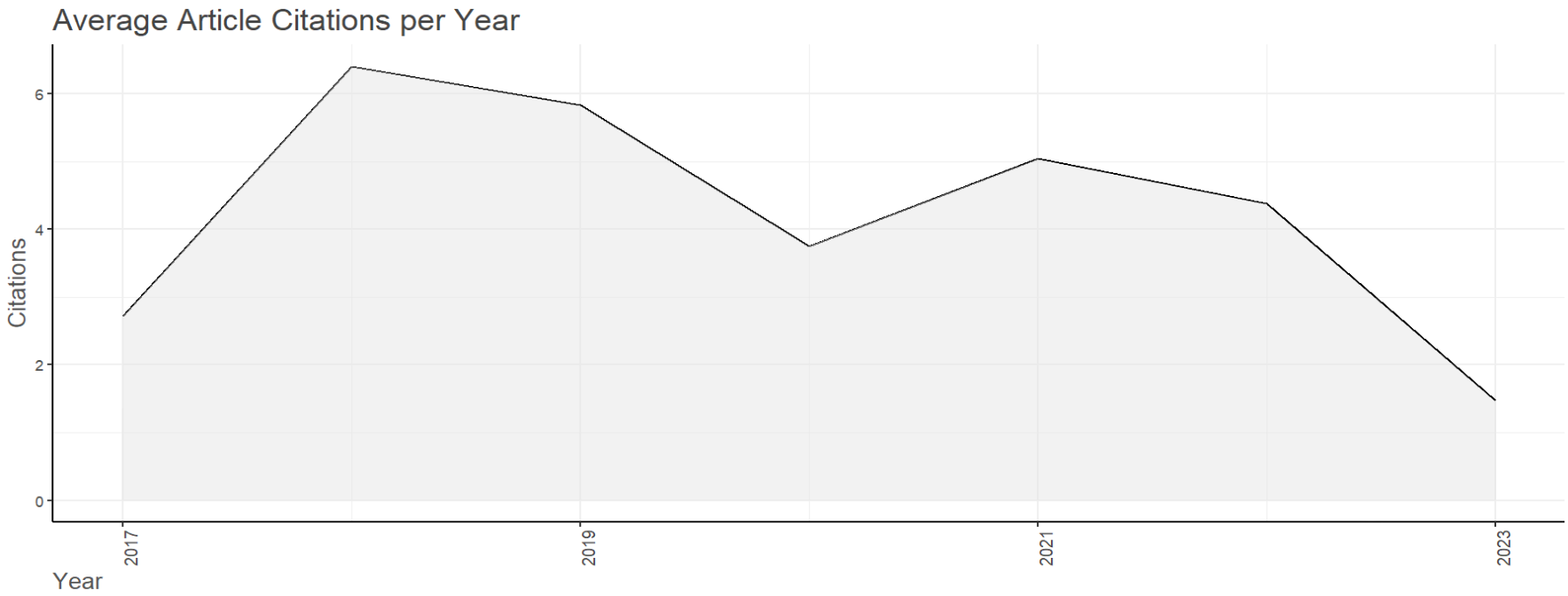


4. Figure: 3. Figure: Average article citations / year of the reviewed studies (n = 77)

*Research context of NOVA application with other sustainable diet indicators – keyword co-occurrences*

The following network visualizes the co-occurrence of author keywords from the selected publications. The network of co-occurrence of author keywords demonstrates the context and structure of research focusing on the NOVA classification. The concepts appearing in the network are divided into five clusters. One cluster includes food price and economic affordability, along with dietary quality indices such as nutrient and energy density, and nutrient-rich food. Another cluster comprises concepts related to the food environment, food advertising, public health, and nutritional quality connected to sustainability. At the center of another cluster are the UPFs and NOVA classification, which is linked to the keywords related to healthiness indicators such as Nutri-Score, nutrient profiling, health star rating and non-communicable diseases, obesity, and traditional food (Figure 4.).


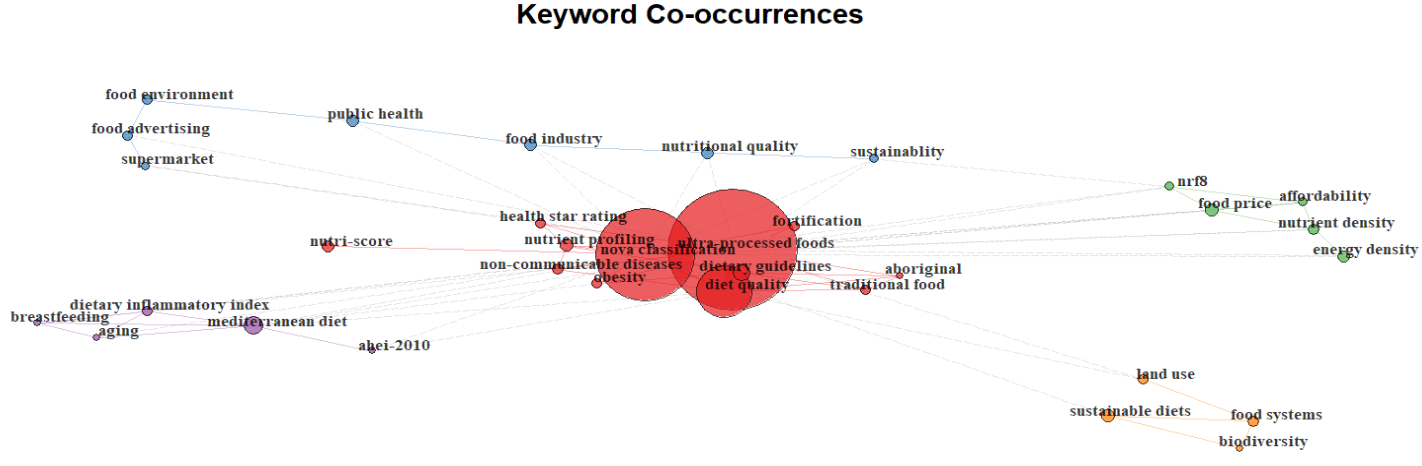


Figure 4.: Keyword-co-occurences based on authors’ keywords from the reviewed studies (n = 77)

References:

S1 Abreu S, Liz Martins M. Cross-Classification Analysis of Food Products Based on Nutritional Quality and Degree of Processing. Nutrients. 2023;15(14):3117. doi:10.3390/nu15143117.

S2 Aceves-Martins M, Bates RL, Craig LC, et al. Nutritional quality, environmental impact and cost of ultra-processed foods: a UK food-based analysis. Int. J. Environ. Res. Public Health. 2022;19(6):3191. doi:10.3390/ijerph19063191

S3 Angelino D, Dinu M, Gandossi B, et al. Processing and nutritional quality of breakfast cereals sold in Italy: results from the Food Labelling of Italian Products (FLIP) Study. Nutrients. 2023;15(8):2013. doi:10.3390/nu15082013

S4 Baldridge AS, Huffman MD, Taylor F, et al. The healthfulness of the US packaged food and beverage supply: a cross-sectional study. Nutrients. 2019;11(8):1704. doi:10.3390/nu11081704

S5 Barrett EM, Gaines A, Coyle DH, et al. Comparing product healthiness according to the Health Star Rating and the NOVA classification system and implications for food labelling systems: An analysis of 25 486 products in Australia. Nutr Bull. 2023;48(4):523-534. doi:10.1111/nbu.12640. Epub 2023 Oct 28

S6 Batal M, Johnson-Down L, Moubarac JC, et al. Quantifying associations of the dietary share of ultra-processed foods with overall diet quality in First Nations peoples in the Canadian provinces of British Columbia, Alberta, Manitoba and Ontario. Public Health Nutr. 2018;21(1):103-113. doi:10.1017/S1368980017001677

S7 Batista CHK, Leite FHM, Borges CA. Association between advertising patterns and ultra-processed food in small markets. Ciência & Saúde Coletiva, 2022;27:2667-2678. doi:10.1590/1413-81232022277.19122021.

S8 Baye K, Yaregal Z. The Global Diet Quality Score predicts diet quality of women of reproductive age in Addis Ababa, Ethiopia. BJN. 2023;130:1573-1579. doi:10.1017/S0007114523000508.

S9 Berardy A, Fresán U, Matos RA, et al. Environmental impacts of foods in the Adventist health study-2 dietary questionnaire. Sustainability. doi:2020;12:10267. doi:10.3390/su122410267

S10 Blanchet R, Willows N, Johnson S, et al. Traditional food, health, and diet quality in Syilx Okanagan adults in British Columbia, Canada. Nutrients. 2020;12:927. doi:10.3390/nu12040927

S11 Bleiweiss-Sande R, Chui K, Evans EW, et al. Robustness of Food Processing Classification Systems. Nutrients. 2019;11:1344. doi:10.3390/nu11061344.

S12 Bonaccio M, Di Castelnuovo A, Ruggiero E, et al. Joint association of food nutritional profile by Nutri-Score front-of-pack label and ultra-processed food intake with mortality: Moli-sani prospective cohort study. BMJ. 2022,378. doi:10.1136/bmj-2022-070688

S13 Braesco V, Souchon I, Sauvant P, et al. Ultra-processed foods: how functional is the NOVA system?. Eur. J. Clin. Nutr. 2022;76(9):1245-1253. doi:10.1038/s41430-022-01099-1

S14 Cediel G, Reyes M, Corvalán C, et al. Ultra-processed foods drive to unhealthy diets: evidence from Chile. Public Health Nutr. 2021;24(7):1698-1707. doi:10.1017/S1368980019004737

S15 Chen YC, Huang YC, Lo YTC, et al. Secular trend towards ultra-processed food consumption and expenditure compromises dietary quality among Taiwanese adolescents. Food Nutr Res. 2018,62. doi:10.29219/fnr.v62.1565

S16 Cooper SL, Pelly FE, Lowe JB. Assessment of the construct validity of the Australian Health Star Rating: a nutrient profiling diagnostic accuracy study. Eur. J. Clin. Nutr. 2017;71(11):1353-1359. doi:10.1038/ejcn.2017.23

S17 da Rocha BRS, Rico-Campà A, Romanos-Nanclares A, et al. Adherence to Mediterranean diet is inversely associated with the consumption of ultra-processed foods among Spanish children: The SENDO project. Public Health Nutr. 2021;24:3294-3303. doi:10.1017/S1368980020001524

S18 da Silva JT, Garzillo JMF, Rauber F, et al. Greenhouse gas emissions, water footprint, and ecological footprint of food purchases according to their degree of processing in Brazilian metropolitan areas: a time-series study from 1987 to 2018. Lancet Planetary Health. 2021,5:775-785.

S19 Davidou S, Christodoulou A, Fardet A, Frank K. The holistico-reductionist Siga classification according to the degree of food processing: an evaluation of ultra-processed foods in French supermarkets. Food Funct. 2020;11(3):2026-2039. doi:10.1039/C9FO02271F

S20 de Las Heras-Delgado S, Shyam S, Cunillera È, et al Are plant-based alternatives healthier? A two-dimensional evaluation from nutritional and processing standpoints. Food Res Int. 2023;169:112857. doi:10.1016/j.foodres.2023.112857

S21 de Moraes MM, Oliveira B, Afonso C, et al. An ultra-processed food dietary pattern is associated with lower diet quality in Portuguese adults and the elderly: The UPPER project. Nutrients. 2021;13:4119. doi:10.3390/nu13114119.

S22 Delgado-Rodríguez R, Moreno-Padilla M, Moreno-Domínguez S, Cepeda-Benito A. Food addiction correlates with emotional and craving reactivity to industrially prepared (ultra-processed) and home-cooked (processed) foods but not unprocessed or minimally processed foods. Food Qual Prefer. 2023;110:104961. doi:10.1016/j.foodqual.2023.104961

S23 Derbyshire, E. Are all ‘ultra-processed’foods nutritional demons? A commentary and nutritional profiling analysis. Trends Food Sci Technol. 2019;94:98-104. doi:10.1016/j.tifs.2019.08.023

S24 Detopoulou P, Dedes V, Pylarinou I, et al. Dietary acid load is associated with waist circumference in university students with low adherence to the Mediterranean diet: The potential role of ultra-processed foods. Clin Nutr ESPEN. 2023;56:43-51. doi:10.1016/j.clnesp.2023.05.005

S25 Dickie S, Woods J, Machado P, Lawrence M. Nutrition classification schemes for informing nutrition policy in Australia: nutrient-based, food-based, or dietary-based?. Curr Dev Nutr. 2022;6(8):112. doi:10.1093/cdn/nzac112

S26 Dinu M, Tristan Asensi M, Pagliai G, et al. Consumption of ultra-processed foods is inversely associated with adherence to the Mediterranean diet: a cross-sectional study. Nutrients. 2022;14:2073. doi:10.3390/nu14102073

S27 Estell ML, Barrett EM, Kissock KR, et al. Fortification of grain foods and NOVA: the potential for altered nutrient intakes while avoiding ultra-processed foods. Eur J Nutr. 2022;1-11. doi:10.1007/s00394-021-02701-1

S28 Fardet A, Rock E. How to protect both health and food system sustainability? A holistic ‘global health’-based approach via the 3V rule proposal. Public Health Nutr. 2020;23:3028-3044. doi:10.1017/S136898002000227X

S29 Fardet A, Méjean C, Labouré H, et al. The degree of processing of foods which are most widely consumed by the French elderly population is associated with satiety and glycemic potentials and nutrient profiles. Food Funct. 2017;8:651-658. doi:10.1039/c6fo01495j

S30 Gallegos-Riofrío CA, Waters WF, Carrasco A, et al. Caliata: an Indigenous Community in Ecuador offers lessons on food sovereignty and sustainable diets. Curr Dev Nutr. 2021;5:61-73. doi:10.1093/cdn/nzab009

S31 García S, Pastor R, Monserrat-Mesquida M, et al. Ultra-processed foods consumption as a promoting factor of greenhouse gas emissions, water, energy, and land use: A longitudinal assessment. Sci Total Environ. 2023;891:164417. doi:10.1016/j.scitotenv.2023.164417

S32 Garzillo JMF, Poli VFS, Leite FHM, et al. Ultra-processed food intake and diet carbon and water footprints: a national study in Brazil. Revista de saude publica, 2022;56:6. doi:10.11606/s1518-8787.2022056004551

S33 Grech A, Rangan A, Allman-Farinelli M, et al. A Comparison of the Australian Dietary Guidelines to the NOVA Classification System in Classifying Foods to Predict Energy Intakes and Body Mass Index. Nutrients. 2022;14:3942. doi:10.3390/nu14193942

S34 Gupta S, Hawk T, Aggarwal A, Drewnowski A. Characterizing ultra-processed foods by energy density, nutrient density, and cost. Front Nutr. 2019;6:454858. doi:10.3389/fnut.2019.00070

S35 Gupta S, Rose CM, Buszkiewicz J, et al. Characterising percentage energy from ultra-processed foods by participant demographics, diet quality and diet cost: Findings from the Seattle Obesity Study (SOS) III. BJN. 2021;126:773-781. doi:10.1017/S0007114520004705

S36 Hallinan S, Rose C, Buszkiewicz J, Drewnowski A. Some ultra-processed foods are needed for nutrient adequate diets: linear programming analyses of the Seattle obesity study. Nutrients. 2021;13:3838. doi:10.3390/nu13113838

S37 Hässig A, Hartmann C, Sanchez-Siles L, Siegrist M. Perceived degree of food processing as a cue for perceived healthiness: the NOVA system mirrors consumers’ perceptions. Food Qual Prefer. 2023;110:104944. doi:10.1016/j.foodqual.2023.104944

S38 Julia C, Baudry J, Fialon M, et al. Respective contribution of ultra-processing and nutritional quality of foods to the overall diet quality: results from the NutriNet-Santé study. Eur J Nutr. 2023;62:157-164. doi:10.1007/s00394-022-02970-4

S39 Juul F, dos Santos Simões B, Litvak J, et al. Processing level and diet quality of the US grocery cart: is there an association?. Public Health Nutr. 2019;22:2357-2366. doi:10.1017/S1368980019001344

S40 Juul F, Lin Y, Deierlein AL, et al. Trends in food consumption by degree of process. BJN. 2021;126:1861-1871. doi:10.1017/S000711452100060X

S41 Kesse-Guyot E, Allès B, Brunin J, et al. Environmental impacts along the value chain from the consumption of ultra-processed foods. Nature Sustainability. 2023;6(2):192-202. doi:10.1038/s41893-022-01013-4

S42 Lavigne-Robichaud M, Moubarac JC, Lantagne-Lopez S, et al. Diet quality indices in relation to metabolic syndrome in an Indigenous Cree (Eeyouch) population in northern Québec, Canada. Public Health Nutr. 2018;21(1):172-180. doi:10.1017/S136898001700115X

S43 Liu J, Steele EM, Li Y, et al. Consumption of ultraprocessed foods and diet quality among US children and adults. Am J Prev Med. 2022;62(2):252-264. doi:10.1016/j.amepre.2021.08.014

S44 Maia EG, Passos CMD, Granado FS, et al. Replacing ultra-processed foods with fresh foods to meet the dietary recomendations: a matter of cost?. Cadernos de Saúde Pública. 2022;37:e00107220. doi:10.1590/0102-311X00107220

S45 Marchese L, Livingstone KM, Woods JL, et al. Ultra-processed food consumption, socio-demographics and diet quality in Australian adults. Public Health Nutr. 2022;25(1):94-104. doi:10.1017/S1368980021003967

S46 Martinez-Perez N, Arroyo-Izaga M. Availability, nutritional profile and processing level of food products sold in vending machines in a Spanish public university. Int. J. Environ. Res. Public Health. 2021;18(13):6842. doi:10.3390/ijerph18136842

S47Martinez-Perez C, San-Cristobal R, Guallar-Castillon P, et al. Use of different food classification systems to assess the association between ultra-processed food consumption and cardiometabolic health in an elderly population with metabolic syndrome (PREDIMED-Plus Cohort). Nutrients. 2021;13(7):2471. doi:10.3390/nu13072471.

S48 Martinez-Perez C, Daimiel L, Climent-Mainar C, et al. Integrative development of a short screening questionnaire of highly processed food consumption (sQ-HPF). Int J Behav Nutr Phys Act. 2022;19(1):6. doi:10.1186/s12966-021-01240-6

S49 Mendes C, Miranda L, Claro R, Horta P. Food marketing in supermarket circulars in Brazil: An obstacle to healthy eating. Preventive Med Reports. 2021;21:101304. doi:10.1016/j.pmedr.2020.101304

S50 Mendoza-Velázquez A, Lara-Arévalo J, Siqueira KB, et al. Affordable nutrient density in brazil: nutrient profiling in relation to food cost and NOVA category assignments. Nutrients. 2022;14(20):4256. doi:10.3390/nu14204256

S51 Mignogna C, Costanzo S, Di Castelnuovo A, et al. The inflammatory potential of the diet as a link between food processing and low-grade inflammation: An analysis on 21,315 participants to the Moli-sani study. Clin Nutr. 2022;41:2226-2234. doi:10.1016/j.clnu.2022.08.020

S52 Morales FJ, Mesías M, Delgado-Andrade C. Association between heat-induced chemical markers and ultra-processed foods: A case study on breakfast cereals. Nutrients. 2020;12:1418. doi:10.3390/nu12051418

S53 Otten JJ, Buszkiewicz J, Tang W, et al. The impact of a city-level minimum-wage policy on supermarket food prices in Seattle-King County. Int. J. Environ. Res. Public Health. 2017;14:1039. doi:10.3390/ijerph14091039

S54 Phulkerd S, Dickie S, Thongcharoenchupong N, et al. Choosing an effective food classification system for promoting healthy diets in Thailand: a comparative evaluation of three nutrient profiling-based food classification systems (government, WHO, and Healthier Choice Logo) and a food-processing-based food classification system (NOVA). Front Nutr. 2023;10:1149813. doi:10.3389/fnut.2023.1149813

S55 Phulkerd S, Thongcharoenchupong N, Dickie S, et al. Profiling ultra-processed foods in Thailand: sales trend, consumer expenditure and nutritional quality. Global Health. 2023;19:64. doi:10.1186/s12992-023-00966-1

S56 Pulker CE, Trapp GS, Scott JA, Pollard CM. Alignment of supermarket own brand foods’ front-of-pack nutrition labelling with measures of nutritional quality: An Australian perspective. Nutrients. 2018;10:1465. doi:10.3390/nu10101465

S57 Rizzolo-Brime L, Orta-Ramirez A, Puyol Martin Y, Jakszyn P. Nutritional assessment of plant-based meat alternatives: a comparison of nutritional information of plant-based meat alternatives in Spanish supermarkets. Nutrients. 2023;15:1325. doi:10.3390/nu15061325

S58 Robert M, Shankland R, Bellicha A, et al. Associations between resilience and food intake are mediated by emotional eating in the NutriNet-Santé Study. Journal of Nutr. 2022;152:1907-1915. doi:10.1093/jn/nxac124

S59 Rodrigues VM, Rayner M, Fernandes AC, et al. Nutritional quality of packaged foods targeted at children in Brazil: which ones should be eligible to bear nutrient claims?. Int J Obes. 2017;41:71-75. doi:10.1038/ijo.2016.167

S60 Romero Ferreiro C, Lora Pablos D, Gómez de la Cámara A. Two dimensions of nutritional value: Nutri-Score and NOVA. Nutrients. 2021;13: 2783. doi:10.3390/nu13082783

S61 Rossato SL, Khandpur N, Lo CH, et al. Intakes of unprocessed and minimally processed and Ultraprocessed food are associated with diet quality in female and male health professionals in the United States: A prospective analysis. J Acad Nutr Diet. 2023;123:1140-1151. doi:10.1016/j.jand.2023.03.011

S62 Ruggiero E, Esposito S, Costanzo S, et al. Ultra-processed food consumption and its correlates among Italian children, adolescents and adults from the Italian Nutrition & Health Survey (INHES) cohort study. Public Health Nutr. 2021;24:6258-6271. doi:10.1017/S1368980021002767

S63 Salomé M, Arrazat L, Wang J, et al. Contrary to ultra-processed foods, the consumption of unprocessed or minimally processed foods is associated with favorable patterns of protein intake, diet quality and lower cardiometabolic risk in French adults (INCA3). Eur J Nutr. 2021;60:4055-4067. doi:10.1007/s00394-021-02576-2

S64 Shim JS, Shim SY, Cha HJ, et al. Association between ultra-processed food consumption and dietary intake and diet quality in Korean adults. JAcad Nutr Diet. 2022;122:583-594. doi:10.1016/j.jand.2021.07.012

S65 Siqueira KB, Borges CA, Binoti ML, et al. Nutrient density and affordability of foods in Brazil by food group and degree of processing. Public Health Nutr. 2021;24(14):4564-4571. doi:10.1017/S1368980020004358

S66 Sneed NM, Ukwuani S, Sommer EC, et al. Reliability and validity of assigning ultraprocessed food categories to 24-h dietary recall data. Am J Clin Nutr. 2023;117:182-190. doi:10.1016/j.ajcnut.2022.10.016.

S67 Spiteri SA, Olstad DL, Woods JL. Nutritional quality of new food products released into the Australian retail food market in 2015–is the food industry part of the solution? BMC Public Health, 2018;18:1-10. doi:10.1186/s12889-018-5127-0

S68 Trübswasser U, Talsma EF, Ekubay S, et al. Factors influencing adolescents' dietary behaviors in the school and home environment in Addis Ababa, Ethiopia. Front Public Health. 2022;10:861463. doi:10.3389/fpubh.2022.861463

S69 Valenzuela A, Zambrano L, Velásquez R, et al. Discrepancy between food classification systems: Evaluation of Nutri-Score, NOVA classification and chilean front-of-package food warning labels. Int. J. Environ. Res. Public Health. 2022,19:14631. doi:10.3390/ijerph192214631

S70 Van Dam I, Vandevijvere S. Benchmarking the nutrition-related commitments and practices of major French food companies. BMC Public Health, 2022;22:1435. doi:10.1186/s12889-022-13780-y

S71 Van Dam I, Reimes N, Vandevijvere S. Benchmarking the nutrition-related commitments and practices of major Belgian food companies. Int J Behav Nutr Phys Act. 2022;19:43. doi:10.1186/s12966-022-01269-1

S72 Vandevijvere S, Pedroni C, De Ridder K, Castetbon, K. The cost of diets according to their caloric share of ultraprocessed and minimally processed foods in Belgium. Nutrients. 2020;12:2787. doi:10.3390/nu12092787

S73 Vellinga RE, van Bakel M, Biesbroek S, et al. Evaluation of foods, drinks and diets in the Netherlands according to the degree of processing for nutritional quality, environmental impact and food costs. BMC Public Health. 2022;22: 877. doi:10.1186/s12889-022-13282-x

S74 Vellinga RE, van den Boomgaard I, Boer JM, Different Levels of Ultraprocessed Food and Beverage Consumption and Associations with Environmental Sustainability and All-cause Mortality in EPIC-NL. Am J Clin Nutr. 2023;118(1):103-113. doi:10.1016/j.ajcnut.2023.05.02Siqueira KB, Borges CA, Binoti ML, et al. Nutrient density and affordability of foods in Brazil by food group and degree of processing. Public Health Nutr. 2021;24(14):4564-4571. doi:10.1017/S1368980020004358

S75 Vergeer L, Veira P, Bernstein JT, et al. The calorie and nutrient density of more-versus less-processed packaged food and beverage products in the Canadian food supply. Nutrients. 2019;11:2782. doi:10.3390/nu11112782

S76 Vicente B M, Almeida Bastos A, de Melo CM, et al. Correlation Between Different Dietary Indexes, and Their Association with An Anti-inflammatory Biomarker in Older Adults: An Exploratory Study. Eur J Geriatr Geront. 2023;5(3). doi:10.4274/ejgg.galenos.2023.2022-10-5

S77 Vogliano C, Raneri JE, Maelaua J, et al. Assessing diet quality of indigenous food systems in three geographically distinct solomon islands sites (Melanesia, Pacific Islands). Nutrients. 2020;13:30. doi:10.3390/nu13010030
