# Supplementary material for: Fifteen Years of NOVA Food-Processing Classification: “Friend or Foe” Among Sustainable Diet Indicators? A Scoping Review
Source: Nutr Rev. 2025 Jan 23;83(4):771–91. doi: 10.1093/nutrit/nuae207 (PMC11894255; doi:10.1093/nutrit/nuae207)
Supplement: nuae207_Supplementary_Data [file nuae207_supplementary_data.zip › nuae207_Supplementary_Data/Table_S1_General_and_specific_aspects_of_the_reviewed_studies.docx]

| **Short reference** | **Country/regio** | **Aims of the study** | **Target population** | **Study design** | **Relevant statistical analysis** | **Data source** | **Source of dietary data** | **Dietary level** | **Level of food item aggregation (shown in the results)** | **Variables calculated based on NOVA classification** | **Variables of NOVA shown in comparative results** | **Other dietary and sustainability metrics** | **proportion of foods classified as NOVA4 (UPF) in the total sample** |
| --- | --- | --- | --- | --- | --- | --- | --- | --- | --- | --- | --- | --- | --- |
| Abreu & Martins, 2023^1^ | Portugal | To compare the classiﬁcation of foods available in the Portuguese market using Nutri-Score and NOVA classiﬁcations and to analyse their ability to discriminate the fat, saturated fat, sugar, and salt content of foods. | NA | cross-sectional | descriptive statistics, categorical principal component analysis (CATPCA) and Spearman’s correlation coefﬁcients | presential or market websites | 12 groups and 27 subgroups, considering the classiﬁcation of FoodEx 2 | foods (n = 2682) | 2682 food products, 12 food groups, 28 food subgroup | classification according to degree of processing; unprocessed/minimally processed foods (group 1), processed culinary ingredients (group 2), processed foods (group 3), and ultra-processed foods (UPF) (group 4) | G1,2,3&4 | Nutri-Score, Multiple Trafﬁc Lights system | The group with the highest frequency (84.8%) was UPF (NOVA 4). |
| Aceves-Martins et al., 2022^2^ | UK | To determine how ultra-processed and processed foods compare to fresh and minimally processed foods in relation to nutritional quality, greenhouse gas emissions and cost on the food and food group level. | NA | cross-sectional | Shapiro–Wilk tests, Kruskal–Wallis tests | National Diet and Nutrition Survey nutrient databank year 11 (2018/2019) | National Diet and Nutrition Survey (NDNS) nutrient databank | foods (n ~ 6000 commonly consumed foods and drinks and prepared dishes) | Individual food and drinks from the NDNS nutrient databank were mapped (based on their main components) into five food groups. Additionally, one group of drinks was created, and one more with food or drink items (e.g., sauces, ketchup) that should be eaten less often and in small amounts; food subgroups: 22 | classification according to degree of processing (based on NOVA); 1,2,3 and 4-> from the analysis, group 2 was excluded | G1,3&4 | nutritional quality (NRF8.3 index), greenhouse gas emission and cost | Thus, 4912 food items were included in this analysis. From these, 20% were categorised as NOVA 1 minimally processed or fresh foods, 32% as NOVA 3 processed foods, and 48% as NOVA 4 ultra-processed foods. |
| Angelino et al., 2023^3^ | Italy | To compare the level of processing (as assessed by the NOVA classification) and the nutritional quality (as assessed by nutrition values, Nutri-Score and NutrInform battery) of breakfast cereals currently on the Italian market. | NA | cross-sectional | descriptive statistics, Kolmogorov–Smirnov test, Mann–Whitney non-parametric test, Kruskal–Wallis test, Principal Component (PC) analysis | Food Labelling of Italian Products (FLIP)FLIP project 2022 | nutrition declarations of breakfast cereal items, available in 13 retailers present on the Italian market | foods (n = 349) | food groups (n = 5) among breakfast cereals | classification according to degree of processing (based on NOVA); 1,2,3 and 4-> there was no food in group 2 in the sample so was not in the analysis | G1,3&4 | Nutri-score, NutrInform battery, presence of Nutrition Claim | A total of 349 items were found, mostly belonging to the NOVA 4 group (66.5%) |
| Baldridge et al., 2019^4^ | USA | To perform a cross-sectional assessment of the state of the US packaged food and beverage supply by reporting the nutritional composition and indicators of healthfulness and processing across the country’s largest food and beverage manufacturers. | NA | cross-sectional | descriptive statistics, frequencies by categories | online database | Label Insight’s Open Data database | foods (n = 230156) | major food categories (n = 15) | classification according to degree of processing; group 1,2, 3 and 4 | UPF&non-UPF | Health Star Rating (HSR) | The overall mean proportion of products considered ultra-processed was 71%. |
| Barrett et al., 2023^5^ | Australia | To further investigate the extent of alignment and discordance between ‘healthier’ products as signalled by the HSR and NOVA systems using a large and representative sample of packaged foods and beverages available in Australia. The second aim was to consider the implications of any discordance for potential approaches to integrating processing information into nutrient classification systems, such as the HSR, and/or integrating nutrient and food ingredient information into the NOVA system. | NA | cross-sectional | descripitve statitics, agrrement analysis with κ statistic, two-tailed t-test | NA | George Institute for Global Health's Australian 2022 FoodSwitch Dataset | foods (n = 25 486) | food groups (n = 15) | classification according to degree of processing (based on NOVA); 1,2,3 and 4 | UPF&non-UPF | Health Star Rating (HSR) | Almost two-thirds (64.2%, n= 16 371) of products were determined as NOVA group 4. |
| Batal et al., 2018^6^ | Canada provinces | To quantify associations of the dietary share of ultra-processed foods (UPF) with the overall diet quality of First Nations peoples. | First Nations peoples in the Canadian provinces of British Columbia, Alberta, Manitoba and Ontario, 19 years or older (n = 3700) | cross-sectional | descriptive statistics,Trend and χ2 analyses, logistic regression | First Nations Food, Nutrition and Environment Study | 24-h dietary recalls | diet (population mean of target population) | 28 food groups | classification according to degree of processing; group 1,2,3 and 4 -> kcal and nutrients density contribution (%) to to total diet from the 4 group (further also assigned as non-uPF and UPF) | G1,2,3&4 and UPF&non-UPF | nutrient quality, traditional food eater | UPF contributed 53.9% of energy. |
| Batista et al., 2022^7^ | Brazilia | To characterize the advertising appeals present in the food environment to market ultra-processed foods and to analyze the nutritional profile of these foods according to PAHO criteria and the presence of food additives. | NA | cross-sectional | descpritive statistics, correlation analysis, linear regression, Exploratory factor analysis, Kaiser-Meyer-Olkin (KMO) test, Cattell’s scree test, Orthogonal rotation | UDITNOVA audit instrument, small grocery stores in three cities | food ingredient lists and nutrient facts labels, manufacturers’ website | UPF foods (n = 18) | food groups (n = 10) | AUDITNOVA, identification of UPF based on NOVA classification | UPF&non-UPF | PAHO Nutrient Profile Model | not relevant: selected sample were UPFs |
| Baye & Yaregal, 2023^8^ | Addis Ababa, Ethiopia | To assess diet quality among non-pregnant non-lactating women of reproductive age (WRA) in Addis Ababa, Ethiopia. | non-pregnant non-lactating women of reproductive age (n = 653) | cross-sectional | descriptive statistics, e multivariate regression model, Pearson/spearman correlation | consumption survey | 24-h dietary recall | diet (population mean by GDQS score classification) | food group (n = 25) | classification according to degree of processing (based on NOVA); 1,2,3 and 4 -> prevalence of UPF consumption (% contribution to total diet) | UPF&non-UPF | dietary diversity score, the Global Diet Quality Score | mean intake 9% |
| Berardy et al., 2020^9^ | North-America | To use life cycle assessment to estimate the environmental impacts (from farm to factory gate) of the 198 hard-coded line-items included in the food frequency questionnaire of the Adventist Health Study-2 survey and to assess differences among food groups. | Adventist population | cross-sectional | descriptive statistics | Adventist Health Study-2 survey | food frequency questionnaire | foods (n = 248) | food groups considered in the AHS2-FFQ (n = 12) | classification according to degree of processing (group 1-4) | G1,2,3&4 | warming potential, land use, and water consumption impacts | Based on NOVA classification, 42% of foods were minimally processed (group 1), 41% were ultra-processed (group 4), 13% were processed (group 3), and 4% were processed culinary ingredients (group 2). |
| Blanchet et al., 2020^10^ | Canada | To describe present TF consumption in a First Nation in Canada, namely among Syilx adults; assess the association of TF consumption with health indicators; and compare diet quality of TF eaters and non-eaters using three methods: nutrient intakes and nutrient adequacy, the HEI-C, and the %E from UPP using the NOVA classification. | Syilx Okanagan Adults (n = 265) | cross-sectional | descriptive statistics, Chi-square tests and t-test, logistic regression analyses, analysis of covariance (ANCOVA) with Bonferroni adjustment to take into account multiple tests | Okanagan Salmon and Our Health Study in 2018, household data | 24-h dietary recall | diet (population means: all, no TFs in diet, TFs ) | traditional foods (n = 21) and non traditional foods (n =?) | classification according to degree of processing; group 1,2, 3 and 4->contributions of ultra-processed products (UPP) to %energy of total diet | G1,2,3&4 and UPF&non-UPF | Healthy Eating Index, traditional foods | UPP accounted for 60.6% of energy (SE 1.5%, range 0–100%), unprocessed or minimally processed foods for 27.6% of energy (SE 1.3%, range 0–87.5%), processed products for 6.6% of energy (SE 0.6%, range 0–62.0%), and culinary ingredients for 5.1% of energy (SE 0.5%, range 0–64.1%). |
| Bleiweiss-Sande et al., 2019^11^ | USA | To evaluate the robustness of processing classification systems and to assess their utility as a measure of healthfulness in children’s diets. The objectives of this study were to (1) investigate the inter-rater reliability of three food processing classification systems, (2) compare classification agreement between the three systems using the top 100 most commonly consumed foods among children in the U.S., and (3) determine whether nutrient concentrations were predictive of each system’s processing categorization for the top 100 foods. | children under nine years old | cross-sectional | inter-rater reliability; Spearman’s rank correlation coefficient, linear discriminant analysis and multinomial logistic regression, Cohen’s kappa coefficient | NHANES 2013–2014 | most commonly consumed foods children consume | foods (n = 100) | no further aggregation | classification according to degree of processing; group 1,3 and 4 | G1,3&4 (G1 and 2 merged) | International Food Information Council, and University of North Carolina at Chapel Hill; nutritional quality (encouraged and discouraged nutrients) | The Nova system classified the most foods as highly processed (70%) compared to the UNC (62%) and IFIC (53%) systems. |
| Bonaccio et al., 2022^12^ | Molise, a southern Italian region | To jointly analyse two food dimensions, the Food Standards Agency Nutrient Profiling System (FSAm- NPS), used to derive the Nutri-Score front-of-pack label, and the NOVA classification in relation to mortality. | general population (n = 22895) | Prospective cohort | descriptive statistics, correlation analysis, specific mortality through multivariable cause specific Cox proportional hazards, multivariable models, sensitivity analyses | Moli-sani Study | semiquantitative European Prospective Investigation into Cancer and Nutrition (EPIC) food frequency questionnaire (FFQ) | diet (population mean quarters by dietary quality) | MedDiet food components (n = 8) | classification according to degree of processing; group 1,2, 3 and 4-> UPF consumption: proportion (%) of ultra-processed food in the total weight of food and beverages consumed (g/d) by creating a weight ratio | UPF&non-UPF | Food Standards Agency Nutrient Profiling System, adherence to a Mediterranean diet | The mean ultra-processed food weight ratio was 10.8% (6.7%); the average energy from ultra-processed foods was 18.3% (8.4%; range 8.4-70.0%) of the total calories consumed daily. |
| Braesco et al., 2022^13^ | France | Explore the robustness and functionality of the NOVA classiﬁcation system. | French specialists (n = 159 and 177) | cross-sectional | Chi-squared tests and Kruskal-Wallis test | online survey data | database of commercially available packaged foods in France (OQALI. French Observatory of Food Quality) | marketed food products (n = 120), and generic foods (n = 111) | no further aggregation | classification according to degree of processing;(NOVA1, NOVA2, NOVA3, or NOVA4) | G1,2,3&4 | Nutri-Score, SAIN-LIM, Nutrient Rich Food | Most of the marketed foods were assigned to NOVA4 (80.0% of the 19,080 assignments). |
| Cediel et al., 2021^14^ | Chile | To assess the consumption of ultra-processed foods and its association with the overall dietary content of nutrients related to non-communicable diseases (NCD) in the Chilean diet and to estimate the population attributable fraction of ultra-processed food consumption on the unhealthy nutrient content. | Chilean population aged ≥2 years (n = 4920). | cross-sectional | descriptive statistics, Crude- and sociodemographic-adjusted standardised regression coefficients (β), Poisson regression models | national survey (2010) | 24-h dietary recalls | diet (population mean of quintiles by the dietary share of ultra-processed foods (% of the total energy intake)) | 36 food groups | classification according to degree of processing (based on NOVA); 1,2,3 and 4 | UPF&non-UPF | NCD-promoting and -protective nutrients | In Chile, ultra-processed foods represented 28.6 % of the total energy intake. The mean dietary contribution of ultra-processed foods for the overall population was 28·6 % ranging from 3-8% kJ to 60.1% kJ. |
| Chen et al., 2018^15^ | Taiwan | To compare two Nutrition and Health Surveys in Taiwan (NAHSITs) 15–18 years apart to evaluate secular changes in ultra-processed food (UPF) consumption and expenditure among Taiwanese adolescents aged 16–18 years and the influences of such changes on dietary quality. | senior high school students (1993-1996, n = 788; NAHSIT 2011, n = 1274) | cross-sectional from two different dates | descriptive statistics, one-way analysis of variance (ANOVA), linear regression analyses, logistic regression | Nutrition and Health Surveys in Taiwan (NAHSITs): 1993-96 and 2011 | 24-h dietary recall | diet (population means by gender and UPF intake % quritles) | YHEI-TwR-90 food components (n = 8) | classification according to degree of processing; group 1,2, 3 and 4-> UPF intake-> population quartiles based on it | G1,2,3&4 | Youth Healthy Eating Index–Taiwan Revised, UPF expenditure | Compared to 1993–1996, adolescents consumed less energy from original foods (55 vs. 39%) but more from processed foods (12 vs. 18%) and UPFs (21 vs. 25%) in 2011, with no apparent gender differences. |
| Cooper et al., 2017^16^ | Australia | To examine the construct validity of the Health Star Rating (HSR) system by determining its diagnostic accuracy and to detect the optimal HSR cutoff points to deﬁne healthiness in packaged dairy foods. | NA | cross-sectional | diagnostic accuracy | dairy foods for sale in an Australian regional supermarket | dairy foods that meet the calcium criteria of the Australian HSR calculator for dairy foods | foods (dairy foods, n = 621) | 14 dairy categoires | classification according to degree of processing; group 1,2,3 and 4-> further binary classification: non-UPF and UPF | UPF&non-UPF | Health Star Rating (HSR) | Of the 621 packaged dairy foods, 415 (67%) were classiﬁed as ultra-processed, with the highest proportion (90%) from yoghurts and other dairy, 73% from dairy beverages and 40% from cheeses. |
| da Rocha et al., 2021^17^ | Spain | To assess whether higher adherence to the traditional Mediterranean diet (MedDiet) was associated with lower consumption of ultra-processed foods (UPF) and lower free sugar intake. | children (52 % boys) (n = 7 386) | cross-sectional analysis of a cohort study | χ2 tests or Student’s t tests, Linear trend tests, Generalised estimating equations with Gaussian distribution, multivariable adjusted model, Pearson's correlation analysis, sensitivity analyses | SENDO project, a Spanish paediatric cohort | semi-quantitative FFQ (149 food items) | diet (population mean by adheence to the Med Diet) | food groups (n = 38) | energy contribution of NOVA group1,2,3 and 4 to total diet in kcal (%) | G1,2,3&4 and UPF&non-UPF | Adherence to the Mediterranean Diet-> KIDMED index | Mean energy intake was 2216 (SD 489·6) [9272 kJ/d (SD 2048)] and mean percentages of energy from unprocessed food or MPF, processed culinary ingredients, processed foods and UPF were 47·5, 10·3, 10·0 and 32·2 %, respectively; overall, 32·2 % of the total energy intake came from UPF. |
| da Silva et al., 2021^18^ | Brazil | To assess the temporal trends in greenhouse gas emissions (GHGE), water footprint, and ecological footprint of food purchases in Brazilian metropolitan areas, and how these are affected by the amount of food processing. | Brazil households (n?) | time-series study: temporal trends (1987–88, 1995–96, 2002–03, 2008–09, 2017–18) | descriptive statistics, linear regression | five Brazilian Household Budget Surveys (1987–88, 1995–96, 2002–03, 2008–09, 2017–18) | household data (purchase) | diet (population mean of target population) | food subgroups (n = 30) | classification according to degree of processing; unprocessed or minimally processed foods (G1); processed culinary ingredients (G2); processed foods (G3); and ultra-processed foods (G4)-> proportion each NOVA food group contributes to daily kcal per person | G1,2,3&4 | GHGE, water footprint, and ecological footprint per 1000 kcal of food and beverages purchased | Proportion of daily kcal (%): 1987-88: 10%; 1995-96: 11%; 2002-03: 16%; 2008-09:19%; 2017-17:23%. |
| Davidou et al., 2020^19^ | France | To take the NOVA classification a step further through a holistico-reductionist approach, taking into consideration the “matrix” effect (which plays a substantial role in chewing, satiety, synergistic actions of nutrients, transit time and nutrient bioavailability); the quantity of salt, sugar and fat added in recipes; the degree of processing of industrial/culinary food ingredients and additives; and the function, number, and potential health risk of additives; (2) to rationalize the characterization of ultra-processing and the definition of UPFs; and (3) to propose to small and large retailers and the agro-food industry a holisticoreductionist score. | NA | cross-sectional | descriptive statistics | online database | packaged foods in French supermarkets | foods (n= 24932) | food groups of pyramids (n =9) a): Legumes, b) Vegetables, c) Cereals, d) Fruits, e) Nuts, f) Fish, g) Eggs, h) Meats, and i) Dairy-> 7 further technological siga subgroups | classification according to degree of processing; group 1,2, 3 and 4 | NA | Siga algorithm | Among the 24 932 packaged foods, 67% were UPFs and therefore contain at least one MUP. Other foods were either un-/minimally processed or processed. |
| de Las Heras-Delgado et al., 2023^20^ | Spain | To characterize and evaluate the plant-based alternatives available on the market in Spain in comparison to animal products in terms of their nutritional composition and profile, and degree of processing. | NA | cross-sectional | descriptive data, Wilcoxon test and Kruskal-Wallis test, Chi-square, discriminating performance | Spanish ‘Veggie base’, branded food composition database, nutritional label products | plant-based alternatives available on the market in Spain | Foods (n = 2790): Five major Plant-Based Alternative Products (PBAPs) categories (n =922) were compared to animal-based processed (n = 922) and unprocessed (n = 381) homologs. | 14 food groups (proteins sources and their alternatives) | classification according to degree of processing; unprocessed or minimally processed foods (group 1), processed culinary ingredients (group 2), processed foods (group 3), and ultra-processed foods (group 4) | G1,2,3&4 | Food Standard Agency Nutrient Profiling System, Nutri-Score | A total of 36.5% (n = 337) of the PBAPs and 71.7% (n = 661) of animal-based products were classified as ultra-processed (NOVA 4). |
| de Moraes et al., 2021^21^ | Portugal | To identify dietary patterns (DPs) and their associations with sociodemographic factors and diet quality in Portuguese adults and the elderly. | Portuguese adults (n = 3102) and the elderly (n = 750) | cross-sectional | multinomial logistic and linear regressions | National Food, Nutrition and Physical Activity Survey (2015–2016) | two non-consecutive dietary 24 h recalls | dietary pattern (traditional, unhealthy and diet concerns) | food groups (n = 44) | food classified according to NOVA in proportion grams to the total daily diet | G1,2,3&4 | diet quality (average dietary content of total energy, macro and micronutrients) | NA |
| Delgado-Rodríguez et al., 2023^22^ | Spain | To compare the potential addictiveness of a priori classified foods into Ultra-Processed (UP) vs Processed (P) vs Minimally Processed (MP) food categories. | women (n = 169) | cross-sectional | analysis of covariance (ANCOVA) | survey data | pictures of foods | foods (n = 45) | no further aggregation | classification according to degree of processing; classified foods into UP (group 4) vs P (group 3) vs MP (group 1) categories | G1,3&4 | Yale Food Addiction Scale 2.0 | NA |
| Derbyshire, 2019^23^ | UK | Evaluating the NOVA classification system and includes an analysis of 50 foods falling under its definition of being ‘ultra-processed’ yet classified as ‘healthy’ according to the UK Nutrient Profiling Model. | NA | cross-sectional | spearman rank-correlation | online supermarket database | food manufacturers’ labels | foods (n = 50) | no further aggregation | definition of being UPF according to the NOVA criteria | G4 | UK the Department of HealthNutrient Profiling Model | analyzed sample was UPFs |
| Detopoulou et al., 2023^24^ | Greece | To assess the association of PRAL and NEAP with adiposity measures in young adults (19.61 ± 3.15 years, mean ± standard deviation) and explore the implication of Mediterranean diet adherence and ultra-processed foods (UPF) intake in this relation. | students of the University of the Peloponnese (n = 346) | cross-sectional | Kolmogorov-Smirnoff test,frequencies (%), t-test, Mann-Whitney test, Chi-square, Spearman correlation, linear regression models | survey | 156 items semi-quantitative food frequency questionnaire (FFQ) | diet (population mean of target population) | food group (n = 23) | energy contribution of UPF (based on NOVA group 4) to total diet in kcal (%) | UPF&non-UPF | MedDietScore, potential renal acid load and net acid production | UPF intake (% energy) 40.7 ± 13.6 |
| Dickie et al., 2022^25^ | Australia | To compare the agreement of nutrient-, food-, and dietary-based NCSs in their assessment of a food's health potential within the Australian food supply, and examine the conceptual underpinnings and technical characteristics that explain differences in performance. | NA | cross-sectional | qualitative analysis, agreement analysis with Cohen κ coefficient, frequencies | Mintel Global New Products Database and the Australian Food Composition Database (AUSNUT 2011–2012) | Australian food supply | foods (n = 7322) | food categories (n = 15) | classification according to degree of processing; group 1,2,3 and 4 ->further assignemnet to non-UPF and UPF | UPF&non-UPF | the Chilean nutrient profile model, Health Star Rating, Nutri-Score, the WHO European Region’s NPM, and the Pan American Health Organization’s; and Australian Dietary Guidelines | The PAHO NPM classified the highest proportion of the total sample as unhealthy at 77%. The WHO-Euro NPM, NOVA, and Chilean NPM also classified over half the total sample as unhealthy, at 69.8%, 65.6%, and 58.1%, respectively. |
| Dinu et al., 2022^26^ | Mediterranean area (Italy) | To evaluate UPF consumption in a group of Italian adults and to assess the possible relationship between MD adherence and UPF consumption using validated tools specifically designed for these purposes. | middle-aged Italian adults (n = 670) | cross-sectional | descriptive statistics, Mann—Whitney test, Chi—Square, Spearman (R) test, general linear model adjusted | online survey | NOVA Food Frequency Questionnaire (NFFQ) | diet (population means by gender) | 94 items divided into nine categories and 27 food subgroups | classification according to degree of processing (group 1-4), based on it calculation of g/day/person intake of each groups-> propotion of NOVA food groups intake | UPF&non-UPF | adherence to the Mediterranean diet (Medi-Lite score) | The percentage of UPF in the diet was 16.4% corresponding to 299 g of UPF per day. |
| Estell et al., 2021^27^ | Australia | To identify if avoidance of ultra-processed grain foods would alter nutrient intakes in an Australian population and whether sample diets using substitute (non-UPF) foods would be likely to meet nutrient requirements. | Autralisan population of all age groups (n = 12153) | cross-sectional | descriptive statistics, Paired t-tests | National Nutrition and Physical Activity Survey 2011-12 | 24-h dietary recall | diet (population mean by ages group and sex) | no further aggregation | classification according to degree of processing (based on NOVA); 1,2,3 and 4-> non-UPFs and UPFs | UPF&non-UPF | nutritional adequacy of sample diets aligned with the Australian Dietary Guidelines | NA |
| Fardet & Rock, 2020^28^ | France | To define a generic diet to protect human health and food system sus- tainability based on three dimensions: animal:plant ratio, degree of food process- ing and food diversity. | French popuation | NA | diet modelling/dietary scenario | Web of Science database | French Ciqual, American USDA | diet (population standard) | food groups (≥42 different animal and plant-based foods), | kcal UPF intake (based on NOVA) contribution to total diet with the threshold of max 15% | NA | animal- and plant-based food ratio, diet diversity | The median daily ultra-processed energetic percentage at which obesity risk begins to significantly increase is approximately 21·3%. |
| Fardet et al., 2017^29^ | France | Exploring relations between the level of food processing, consumption and nutrient proﬁles and satiety and glycemic potentials among 6686 French elderly people (≥65 years). | French elderly people (≥65 years), (n = 6686) | cross-sectional | Descriptive analysis, ANOVA followed by the post hoc, Tukey’s test for means multiple comparison, Pearson’s correlation coeﬃ- cient | French Nutrinet-Santé study, The ongoing NutriNet-Santé web-based  French cohort was launched in 2009 | 24-h dietary recall | 2688 foods-> generic foods (n = 280) | 280 generic food items | classification according to degree of processing (NOVA group 1,3 and 4)->g2 not included | G1,3&4 | nutrient proﬁles, satiety (Fullness Factor™) and glycemic potential (glycemic index and glycemic glucose equivalent), | 9.45 ± 5.61** (consumption %), referred as G3 (UPF) |
| Gallegos-Riofrío et al., 2021^30^ | Ecuador | To explore, through the indigenous community of Caliata in the Ecuadorian highlands, the factors that support or hinder sustainable Andean food systems. | indigenous community of Caliata | cross-sectional | descriptive analysis, Pearson correlations and linear regressions | qualitative methods (n = 49), agroecology-based site analysis, household surveys (n = 57) | modified 48-h dietray recall | foods | reference food groups (www.choosemyplate.gov): grains, dairy, animal protein, legumes and nuts, fruit, dark-green vegetables, red or orange vegetables, other vegetables, and oils (n = 9) | healthy diet index based on NOVA classification; group 1 includes unprocessed or minimally processed foods, group 2 includes culinary ingredients, group 3 includes processed foods, and group 4 includes ultra-processed products | G1,2,3&4 | parcel size and agrodiversity | The diet in Caliata is based principally on foods in group 1, representing 66% of the total. At the other end of the NOVA spectrum, respondents reported using very few ultra-processed items (group 4), being limited mostly to instant coffee (2.5% of households). If this item is removed, the consumption of ultra-processed foods is minimal. |
| García et al., 2023^31^ | Spain | Assessment of the impact of 2-year changes in UPF consumption on greenhouse gas emissions and water, energy and land use. | Southern European population between the ages of 55–75 years with metabolic syndrome (n = 5879 ) | 2 years longitudinal study | General Lin- ear Models, d one-way ANOVA and Bonferroni's post-hoc, Chi-squared test, trends (change in 2 years) | PREDIMED-plus | 143-item food frequency questionnaire | diet (by population tertiles of differences in percentage of UPF consumption between baseline and at 2-year follow-up) | 10 food groups + 4 NOVA groups | % of UPF (based on NOVA classification) in total diet-> classification into population tertiles (T1, T2, T3) | UPF&non-UPF | greenhouse gas emissions and water, energy and land use, Mediterranean diet adherence | Differences in percentage of UPF consumption between baseline and at 2-year follow-up were distributed in tertiles: Tertile 1 (T1): Maximum %UPF reduction: ≤− 37,839; Tertile 2 (T2): Medium %UPF reduction: from−3.7838 to −0.5537; Tertile 3 (T3): Minimum %UPF reduction: ≥− 0.5536. |
| Garzillo et al., 2022^32^ | Brazil | To study the association between ultra-processed food consumption and carbon and water footprints of the Brazilian diet. | Brazilian population aged ≥ 10 years (n = 32,886) | cross-sectional | crude and adjusted linear regression models and tests for linear trends | Cross-sectional analysis on data collected in 2008–2009 | two 24-hour dietary recalls | diet (means by quintiles of UPF contribution (% of total energy intake)) | main food groups (n = 31) | classification according to degree of processing; group 1,2, 3 and 4-> UPF intake-> population quartiles based on it | G1,2,3&4 | freshwater use in liters (water footprint), missions of greenhouse gases in grams of carbon dioxide equivalent (carbon footprint) | The total energy intake and the energy intake from ultra-processed foods across quintiles of their dietary contribution. This contribution ranged from 1.33% of the total energy intake in the lowest quintile to 44.64% in the highest quintile; population mean: On the other hand, the dietary share of G3 foods increased by 1·1 times over 30 years (from 11·2% in 1987–88 to 12·1% in 2017–18; ptrend=0·0032), while the share of G4 foods increased by 2·3 times in the same period (from 9·8% to 23·0%; ptrend<0·0001). |
| Grech et al., 2022^33^ | Australia | To compare the classification of Australian foods under the two systems, evaluate their performance in predicting energy intakes and body mass index (BMI) in free-living Australians, and relate these outcomes to the protein leverage hypothesis of obesity. | free-living Australians | cross-sectional | Univariate and multivariate linear regression | Australian National Nutrition and Physical Activity Survey | 24 h dietary recall | diet (population mean by tertiles of discretionary and UPF% intake) | 5 food group of Australian Dietary Guideline and discretionary foods | energy (kcal) from UPFs of total diet based on NOVA-> population tertiles based on it | UPF&non-UPF | Australian Dietary Guidelines | Using the NOVA system to classify the disaggregated foods by degree of processing, 935 (32.9%) were classed as minimally processed foods, 60 (2.1%) were classed as culinary ingredients, 354 (12.4%) were classed as processed foods, and 1497 (52.6%) were classed as UPF. |
| Gupta et al., 2019^34^ | USA | To characterize ultra-processed foods by energy density, nutrient density, and monetary cost. | NA | cross-sectional | descriptive statistics, ANOVA, sensitivity analyses | the FHCRC FFQ | 384 component foods of Fred Hutch (FHCRC) food frequency questionnaire (FFQ) | foods (n = 384) | MyPyramid Food Groups (n = 7) | classification according to degree of processing; group 1,2, 3 and 4 | G1,2,3&4 | Nutrient Rich Food index NRF9.3, food prices | More than half of the FFQ component foods (57%) fell into the ultra-processed category, with 33% into unprocessed category, and 7% in processed category. |
| Gupta et al., 2021^35^ | USA | To characterise percentage energy from UP foods by participant socio-economic status (SES), diet quality, self-reported food expenditure and energy-adjusted diet cost | Participants of the population-based Seattle Obesity Study III (n = 755) | cross-sectional | multivariable linear regressions | data of Seattle Obesity Study III | FFQ | diet (population mean by tertiles of diet cost and food expenditure) | 378 FFQ component foods | energy (kcal) from UPFs of total diet based on NOVA | UPF&non-UPF | Healthy Eating Index, Nutrient Rich Food Index 9.3, self-reported food expenditure and energy-adjusted diet cost | Mean percentage of dietary energy from UP foods was 59.7%. |
| Hallinan et al., 2021^36^ | USA | To determine whether nutrient-adequate food patterns could be created using unprocessed foods only, or using ultra-processed foods only. | adult population from King, Pierce, and Yakima Counties in Washington State (n = 857) | cross-sectional | diet optimization with linear programming | Seattle Obesity Study (SOS III) | Fred Hutch food frequency questionnaire comprised the market basket | diet (population mean of SOS III sample) | major food groups (n = 7) | classification according to degree of processing; group 1,2, 3 and 4 | G1,2,3&4 | nutrient densities, cost | In the SOS III sample and in all the models, more than half of dietary energy (>50%) came from ultra-processed foods. |
| Hässig et al., 2023^37^ | Schwitzerland | Examine consumers’ associations with processed foods, as well as how their processing and healthiness perceptions are related to food classification systems (i.e. NOVA) and the nutritional value of foods (i.e. Nutri-Score). | Swiss consumers (n = 498) | cross-sectional | ANOVA | online survey | food products were selected from the largest grocery stores in Switzerland | foods (n = 27) | no further aggregation | classification according to degree of processing; group 1,3 and 4 | G1,3&4 | Nutri-Score, consumer's perception | NOVA group 4: 27/8, is it relevant? Preselected sample (The food products were chosen from five food categories: vegetables, fruit, meat and meat alternative prod­ ucts, grain products, and infant food. In each category, several products were selected that all contained the same base product (i.e., straw­ berries, pies, and wheat) but differed in their processing methods. |
| Julia et al., 2023^38^ | France | To identify the respective effects of the nutritional quality of the foods consumed, the ultra-processed nature of foods and their cross-effect contributing to the overall quality of the diet. | French population (n = 98 454) | cross-sectional of a cohort study | descripitve statistics: variation and residual variations, sensitivity analysis | Web-based French NutriNet-Santé cohort study | 24-h dietary recall | diet (population mean by quintiles of PNNS-GS2) | food groups (n = 27) | ultra-processed nature of the foods consumed (qualifed using the proportion of ultra-processed foods consumed UPFp using the NOVA classifcation->population quintiles based on it | UPF&non-UPF | modified Foods Standards Agency nutrient profile model dietary index, PNNS-GS2 score (for Programme National Nutrition Santé Guideline Score 2) | Overall, the difference in the proportion of ultra-processed foods consumed between the first and last quintiles of PNNS-GS2 score was 6.98 percentage points (from 20.04% of UPFp in quintile 1 to 13.06% UPFp in quintile 5). |
| Juul et al., 2019^39^ | USA | To determine if processing level is associated with diet quality of grocery purchases. | US Nationally representative sample of 3961 households | cross-sectional | descriptive statistics, Pearson’s χ2 test,multivariable linear regression | National Household Food Acquisition and Purchase Survey 2012–2013 | household food purchase data | diet (population mean by households tertiles with the highest v. lowest ultraprocessed food purchases) | food components of HEI (n = 9) | classification according to degree of processing; group 1,2, 3 and 4 -> energy share of each processing level (percentage of energy; %E), population tertiles according to the consumption of UPF | G1,2,3&4 and UPF&non-UPF | Healthy Eating Index-2015 component and total scores, recommendations of the Dietary Guidelines for Americans 2015–2020, foods purchase | Ultra-processed foods provided 55·8 %E of grocery purchases, while minimally processed foods provided 28·4 %E. |
| Juul et al., 2021^40^ | USA | To examine longitudinal trends in food consumption according to degree of processing and diet quality from 1991 to 2008 within an ageing US population, using data from the Framingham Offspring Cohort (FOS). | adults (n = 2893) | longitudinal | relevant statistics, trend analyses (mixed effects models with subject-specific random intercepts), sensitivity analysis, Non-linear trends | Framingham Offspring Cohort | FFQ quadrennially 1991–2008 | diet (population means by gender) | food groups (n = 28) | classification according to degree of processing (based on NOVA); 1,2,3 and 4 | G1,2,3&4 | Dietary Guidelines for Americans Adherence Index 2010 | Participants consumed the majority of daily servings in the form of minimally processed foods, followed by ultraprocessed foods in 1991–2008. Ultraprocessed food consumption declined significantly in the whole sample and among females and males between 1991 and 2008. A trend towards decreased consumption of minimally processed food was also identified in the overall sample and among males and females; however, the magnitude of change was minimal (–0·6 servings/d in the overall sample, Ptrend < 0·001).  Finally, consumption of all ultraprocessed foods, except for ultraprocessed meat products and yoghurt, decreased in the overall sample, and among females and males.(NA: its in the dimension of sevrings/day) |
| Kesse-Guyot et al., 2023^41^ | France | Here, using a representative sample of French adults (2,121 enrolled in the Third French Individual and National Food Consumption survey), we investigate the environmental pressures of diets according to UPF consumption. | French adults | cross-sectional | crude and energy-adjusted ANOVA and ANCOVA models, sensitivity analyses | Third French Individual and National Food Consumption survey | 24-hour dietary recall | diet (population mean by quintiles of quintiles of % UPF intake) | no further aggregation | classification according to degree of processing; group 1,2, 3 and 4-> % UPF intake contribution by weight-> population quintiles based on it | UPF&non-UPF | 14 environmental pressure indicators, Programme National Nutrition Santé—Guidelines Score 2, reflecting the adherence to food-based dietary guidelines, and the Diet Quality Index Based on the Probability of Adequate Nutrient Intake | UPF represented 19% of the diet. |
| Lavigne-Robichaud et al., 2018^42^ | canada | To evaluate the association between diet quality and MetS in adult Eeyouch of Eastern James Bay in Québec, using three methods: the aHEI-2010, FQS and the contribution of UPP to total daily dietary energy intake using the NOVA classification. The secondary objective was to assess the pertinence of these diet quality indices in an Eeyouch context. | Eeyouch (n = 811) from seven James Bay communities (≥18 years old). | cross-sectional | descriptive statistics, Shapiro–Wilk test and non-Gaussian distributions underwent a logarithmic transformation, logistic regressions | Study sample from the 2005–2009 cross-sectional Nituuchischaayihititaau Aschii Environment-and-Health Study | 24-h dietary recalls | diet (population quintiles means by dietary quality scores) | no further aggregation | classification according to degree of processing; group 1,2, 3 and 4->contribution of ultra-processed products (UPP) to total daily dietary energy intake | UPF&non-UPF | alternative-Healthy Eating Index 2010, the Food Quality Score | The contribution (52 %) of UPP to total daily dietary energy intake in our sample. |
| Liu et al., 2022^43^ | USA | To investigate how UPF consumption relates to overall diet quality and major food group consumption among nationally representative samples of U.S. adults and children. | adults (n =10064) children (n = 5919) | cross-sectional | generalized linear regressions | National Health and Nutrition Examination Survey (2015–2018) | 24-hour dietary recall | diet (by population quantiles of contribution of Ultraprocessed Foods to Total Energy Intake) | major food groups (n = 6) | energy (kcal) contribution from UPFs (based n NOVA) of total diet->classified into population quintiles | UPF&non-UPF | American Heart Association score, Healthy Eating Index 2015 | Population was classified by UPF intake population means (<50.2->79.0%). |
| Maia et al., 2022^44^ | Brazilia | To analyze the economic impact of the adoption of optimized and nutritionally balanced diets to Brazilian families, considering the Brazilian dietary guidelines and the economic disparities of the population. | Brazilian Household (55,970 households) | cross-sectional | diet optimization with linear programming | Brazilian Household Budget Survey from 2008-2009 | food consumption(derived from household household purchase data) | diets (population means by income level) | food subgroups (n = 35) | classification according to degree of processing; group 1,2, 3 and 4-> The decision variables were the relative contribution (%) of the four food groups from NOVA system | G1,2,3&4 | diet cost (BRL per 2,000Kcal/person/day), nutritional quality | 17.8% UPF in current (observed diet) mean in population (total not by income levels), optimizied: ≤ 9.1% About half (48.9%) of the calories in the current diet were from unprocessed or minimally processed foods, 24.3% from processed culinary ingredients, 8.9% from processed foods and 17.8% from ultraprocessed foods. |
| Marchese et al., 2022^45^ | Australia | To examine how socio-demographic characteristics and diet quality vary with consumption of ultra-processed foods (UPF) in a cross-sectional nationally representative survey of Australian adults. | Australian adults aged ≥ 19 years (n = 8209) | cross-sectional | Descriptive statistics, Linear regression models, Sensitivity analyses | Australian Health Survey 2011–2013 | 24-h dietary recall | diet (population mean of dietary contribution of ultra-processed foods (% of total dietary energy)) | DGI food components: n = 12 | total energy contributed by UPF (in %) based on NOVA classification | UPF&non-UPF | Dietary Guideline Index; compliance with the 2013 Australian Dietary Guidelines | In 2011–2012, Australian adults consumed an average of 8416 kJ (SE 60·4) per day, 38.8% of which were from UPF. |
| Martinez-Perez & Arroyo-Izaga, 2021^46^ | Spain | To assess the availability, nutritional proﬁle and processing level of food products from vending machines at a Spanish public university and to investigate differences in nutritional proﬁle according to the cost and promotion. | NA | cross-sectional | descriptive statistics, agreement analysis | vending machines at a Spanish public university | vending machines product portfolios | foods (n = 3894) | 3 major categories, 18 categories and 78 subcategories | classification according to degree of processing; group 1,2, 3 and 4-> UPF and non-UPF | UPF&non-UPF | Spanish Agency for Consumption, Food Safety and Nutrition and the United Kingdom nutrient proﬁling model, promotion of products, cost | 73.8% of the items were categorised as “ultra-processed”. |
| Martinez-Perez et al., 2021^47^ | Spain | To assess the impact of the food classification system on the cross-sectional association between UPF consumption and cardiometabolic health using the same data set. We hypothesized that applying different food processing-based classification systems to a data set would result in different associations between UPF consumption and cardiometabolic markers. | PREDIMED-Plus participants (n = 5636) | cross-sectional | descripitive statistics, ANOVA, intra-class correlation coefﬁcients, linear regression models | PREDIMED-Plus study data | 143-item food frequency questionnaire | diet (population means by quintiles of UFP % intake) | 136 food and beverage items | classification according to degree of processing; group 1, 2,3 and 4-> UPF assignment and classiciation to population quintiles accordingly | UPF&non-UPF | food processing-based classiﬁcations: International Agency for Research on Cancer, International Food Information Council and University of North Carolina, Mediterreanen Diet adherence | The IARC system had the highest number of food items included in the UPF group (60.7% of all FFQ food items), followed by IFIC and UNC (31.1% for both), while NOVA had the lowest number of food items classified as UPF (27.4%). |
| Martinez‑Perez et al., 2022^48^ | Spain | To develop a short screening questionnaire of HPF consumption (sQ-HPF) that integrates criteria from the existing food classification systems. | Spanish PREDIMED-Plus trial (n = 4400) | cross-sectional | binomial logistic regression, ROC analysis, exploratory factor analysis (EFA) and Cronbach’s analysis, agreement analysis with weighted kappa coefficients. | Spanish PREDIMED-Plus survey | semi qantitative food frequency questionnaire | 143 items sQ-HPF classification proportions of foods | 29 food groups | classification according to degree of processing (based on NOVA); 1,2,3 and 4-> assignement to population tertiles of low/high processed food (HPF) intake | UPF&non-UPF | food processing-based classification systems: IARC, IFIC and UNC, mediterrean diet adherence | NA |
| Mendes et al., 2021^49^ | Brazil | To characterize the foods advertised in supermarket circulars in Belo Horizonte, Brazil, as well as to analyze the price and discounts applied to the products. | NA | cross-sectional | descriptive statistics, Student t-test, Pearson’s chi-square test | five supermarket chains in 2018 | food advertisements | foods (n = 16280) | food subgroups (n = 44) | classification according to degree of processing; group 1,2, 3 and 4 | G1,2,3&4 | Pan-American Health Organization, food prices | Ultra-processed foods responded for 66.9% of ads, followed by fresh or minimally processed foods with 19.9%, processed foods with 9.5%, and processed culinary ingredients with 3.7%. |
| Mendoza-Velázquez et al., 2022^50^ | Brazilia | Exploring nutrient density, cost, and NOVA category assignments within and across food groups in Brazil. | NA | cross-sectional | descriptive analysis, one-way ANOVAs | Brazil Consumption Expenditure Survey (POF) | Brazil Consumer Expenditure Survey (household level) | foods (n = 591) | food groups (n = 11) | classification according to degree of processing (based on NOVA); 1,2,3 and 4 | G1,2,3&4 | Nutrient Rich Food Index, energy density: (ratio of energy and/or nutrient density of foods and retail price per 100 kcal) | The NOVA assignments were MPF (17.94%), PF (31.81%), UPF (48.39%), and culinary ingredients (1.86%). |
| Mignogna et al., 2022^51^ | Molise region, in Southern Italy | To test the hypothesis that an increasing degree of food processing is directly associated with low-grade inﬂammation, and evaluate to what extent this association is mediated by the inﬂammatory potential of highly processed foods. | (n = 21315) | cross-sectional | multivariable-adjusted linear regression models | survey data of Moli-sani study | 188 item FFQ | foods (n = 88) | 188 food items was classiﬁed into 81 individual food items and food groups | classification according to degree of processing; 1) unprocessed/minimally processed foods; 2) processed culinary ingredients; 3) processed foods; 4) ultra-processed foods (UPF) | G1,2,3&4 | Energy-adjusted Dietary Inﬂammatory Index | The diet of participants was mainly made up of minimally processed foods (61.7%; SD ± 11.8), while processed and ultra-processed foods represented, respectively, 25.2% (SD ± 12.2) and 10.8% (SD ± 6.7) of the total food eaten daily; processed culinary ingredients contributed with 2.3% (SD ± 0.8) to the total. |
| Morales et al., 2020^52^ | Spain | To investigate the link between the degree of processing as described by the international NOVA food classification system and the occurrence of heat-induced chemical markers in breakfast cereals. | NA | cross-sectional | descriptive statistics, Levene’s test and the Shapiro–Wilk W test , Student t-test and analysis of variance (one-way ANOVA) with Bonferroni’s multiple comparisons post hoc test, Pearson’s correlation test, Spearman’s rank correlation test | commercial breakfast cereal samples were purchased from Spanish supermarkets in 2018 | food composition/food label | foods (breakfast cereals, n = 53) | food groups by cereal type (n = 5) | classification according to degree of processing; group 1, 3 and 4 | G1,3&4 | NutriScore, Acrylamide and hydroxymethylfurfural (HMF) content as heat-induced chemical markers | A total of 6 samples (11%) were identified as unprocessed or minimally processed foods (NOVA-1), 16 samples (30%) as processed foods (NOVA-3), and 31 samples (59%) as ultra-processed foods (NOVA-4). |
| Otten et al., 2017^53^ | Seattle (USA) | To examine the effects of increasing minimum wage on supermarket food prices in Seattle over 2 years of policy implementation, overall and differentially across food quality metrics. | NA | intervention study (over 2 years of policy implementation in Seattle) | linear regression models | large supermarket chain stores in Seattle and the same chain stores in King County (“control”) | market baskets | foods (n = 106) | food groups (n = 7) | classification according to degree of processing (based on NOVA); 1,2,3 and 4 (by 3 researcher) | G1,2,3&4 | food prices per 100 kcal, nutrient density quartiles based on the Nutrient Rich Foods Index 9.3 | 106/59 |
| Phulkerd et al., 2023^54^ | Thailand | To assess the nutritional quality of food and beverage products in Thailand by comparing four different food classification systems. | NA | cross-sectional | Descriptive statistics, Cohen’s kappa statistic | online database | Mintel Global New Products Database (Mintel GNPD) | foods (n = 17414) | 17 food categories | classification according to degree of processing; unprocessed or minimally processed foods (MP), processed culinary ingredients (PCI), processed foods (P), and ultra-processed foods (UPF) | UPF&non-UPF | Thailand DOH nutrient profiling, WHO SEA nutrient profile model, HCL nutrient profiling model | NOVA group 4: 76.6%. |
| Phulkerd et al., 2023^55^ | Thailand | To analyse the national trends in retail sales, consumer expenditure and nutritional quality of UPFs in Thailand. | Thai consumers | cross-sectional | descriptive statistics | Euromonitor Passport database and consumer expenditure | Mintel Global New Products Database | packaged food products (n = 17414) | main food categories (n = 15) | classification according to degree of processing; group 1,3 and 4-> further assignment: UPF and non-UPF | UPF&non-UPF | WHO Southeast Asian Region nutrient profile model, consumer expenditure | all food items are UPFs |
| Pulker et al., 2018^56^ | Australia | To examine prevalence of front-of-pack nutrition labels (FOPNL) on supermarket own brand foods (SOBF), and alignment with patterns of nutritional quality. | NA | cross-sectional | descriptive statistics, Chi-square tests | Photographic audits of all supermarket own brand foods (SOBF) | Relevant details were extracted from the images into Excel databases created for each of the supermarkets. | foods (n = 3737) | food groups (n =10) | classification according to degree of processing; group 1,2, 3 and 4-> identification of UPF | G1,2,3&4 | nutritious or nutrient-poor based on the Australian Guide to Healthy Eating, Health Star Ratings | Over half (56.9%) of all SOBF were classified as UPF |
| Rizzolo-Brime et al., 2023^57^ | Spain | To evaluate the nutritional profile and processing degree of PBMAs available in Spain. | NA | cross-sectional | descriptive analysis | food products from seven Spanish supermarkets | product's name, list of ingredients, nutritional content, and organic or conventional farming | foods (n = 7 148) | 8 categories of plant-based meat alternatives | classification according to degree of processing (based on NOVA); 1,2,3 and 4 | G3&4 | nutritional profiling systems: Agencia Española de Seguridad Alimentaria y Nutrición, UK’s FoP labeling, organic/conventional farming | According to the NOVA classification system, 93.9% of the products were categorized as ultra-processed food (Group 4) and the remaining 6.08% of PBMAs were categorized as processed food (Group 3, for the plant-based mince group). |
| Robert et al., 2022^58^ | France | To analyze the associations between resilience and diet quality and ultraprocessed food (UPF) and food group consumption, and to assess whether emotional eating was a mediator of these associations. | general population (n = 17840) | cross-sectional | Student t test, and χ2 test, linear regression models, Pearson correlations for continuous variables and Student t tests and ANOVA | NutriNet-Santé study 2017 | ≥3 self-administered 24-h dietary records | diet (population mean of target population) | food groups (n = 18) | classification according to degree of processing; group 1,2, 3 and 4-> UPF consumption: % of total food intake in kcal | UPF&non-UPF | French National Nutrition and Health Program Guideline Score | consumption of UPFs (33%) in the population |
| Rodrigues et al., 2016^59^ | Brazil | To assess the nutritional quality of food products marketed at children, with and without nutrient claims, using two different approaches. | Brazilian children | cross-sectional | descriptive analysis, agreement analysis (Cohen’s κ-statistic), Pearson's χ2 test | packaged food marketed at children sold in a major Brazilian supermarket | food label | foods (n = 535) | no further aggregation | classification according to degree of processing based on NOVA; : ‘natural or minimally processed foods’, ‘processed foods’ and ‘ultraprocessed foods’-> assignment as "less healthy" and "healthier) | "healthy" (G1) & "less healthy" (G3+G4) | UK/Ofcom nutrient proﬁle | The NOVA model was stricter than the UK/Ofcom model, classifying more products as ‘less healthy’ (91.4%) compared with the nutrient proﬁle-based model (75.0%). |
| Romero Ferreiro et al., 2021^60^ | NA | To compare the nutritional quality (as assessed by Nutri-Score) and the ultra-processing (as assessed by the NOVA classiﬁcation) of foods in the Open Food Facts database. | NA | cross-sectional | correspondance analysis | online database | Open Food Facts database | foods (n = 9931) | 9931 foods, 11 food groups | classification according to degree of processing; NOVA groups (1, 3, 4) | G1,3&4 | Nutri-Score | According to the NOVA classification, the group with the highest frequency was ultra-processed foods (NOVA 4) with 56.45%. |
| Rossato et al., 2023^61^ | USA | To assess whether UMP and UPF intake are associated with three diet-quality metrics in female and male health professionals from two US cohorts over 3 decades of follow-up. | Participants were invited in 1976 (NHS) and 1986 (HPFS) | prospective analysis | Generalized estimating equations | This was a cohort study, including data from the Nurses’ Health Study (NHS),  from 1986 to 2010 (N ¼ 51,956) and the Health Professionals Follow-up Study (HPFS)  from 1986 to 2006 (n ¼ 31,307). | semi-quantitative food frequency questionnaire | diet (means by quintiles of unprocessed and minimally processed food (UMP) intake quintiles in percent of energy) | food components of AHEI-2010 (11 components of the diet; aMED; DASH diet components | classification according to degree of processing based on NOVA; minimally processed food (UMP) and ultra-processed food (UPF) in percent of total energy and percent of total grams and milliliters were presented as quintiles  , ranked into ﬁve levels of exposure, ranging from the lowest (ﬁrst quintile) to the highest level (ﬁfth quintile). | G1&G4 | Alternate Healthy Eating Index, Mediterranean diet index, Dietary ApproachesTo Stop Hypertension diet score | The UMP (40%) and the UPF (24%) represented the most food items. |
| Ruggiero et al., 2021^62^ | Italy | To assess ultra-processed food (UPF) consumption and its sociodemographic, psychosocial and behavioural correlates in a general population of Italian children, adolescents and adults. | general population (5–97 years) (n = 9078) | cross-sectional | descriptive statistics, multivariable-adjusted linear regression analysis | Italian Nutrition & Health Survey (INHES) cohort study | 24-h dietary recall | diet (population mean by age groups) | Further classificatio of UPF (NOVA G4) (n = 17) | classification according to degree of processing; group 1,2, 3 and 4-> UPF contribution: % of total food intake in kcal | UPF&non-UPF | Mediterreanen diet adherence | Average energy intake from UPF (95 % CI) was 17·3% among adults and 25·9 % in children/adolescents. Top sources of UPF were processed meats (32·5 %) and bread substitutes (16·7 %). Among adults, age and residing in Southern Italy inversely associated with UPF. |
| Salomé et al., 2021^63^ | France | To study associations between the degree of food processing, patterns of protein intake, diet quality and cardiometabolic risk. | French adults s (18–79y) (n = 1774) | cross-sectional | descriptive, correlation analysis, ANOVA, multiple linear regression models | Individual and National Study on Food Consumption Survey (INCA3) | three nonconsecutive 24-h dietary recalls | diet (population mean by tertiles of proportion of total energy intake from MPF (MPFp), PF (PFp) or UPF (UPFp)) | Food families were either plant-based (“Refned grains”, “Whole grains”, “Legumes”, “Nuts and seeds”, “Fruits”, “Vegetables”, “Potatoes” and “Other plant foods”; n = () or animal-based (“Red meat”, “Poultry”, “Fish”, “Processed meat”, “Other meats”, “Eggs”, “Cheeses”, “Milk”, “Yogurt”, and “Other dairy products”; n = 10) | energy (kcal) contribution from UPFs (based on NOVA) of total diet->population was stratifed into tertiles in three diferent ways according to the proportion of total energy intake from MPF (MPFp), PF (PFp) or UPF (UPFp) in their diet | G1,3&4 | protein intakes (animal, plant and diversity), PANDiet scoring system, the global Plant-based Diet Indices, healthful and unhealthful plant-based diet indices | In the overall population, MPF contributed to 32.8% (SD: 12.1) of energy intake, PF to 24.9% (SD: 12.2) of energy intake and UPF to 30.6% (SD: 15.8) of energy intake. |
| Shim et al., 2020^64^ | South-Korea | To estimate the energy contribution of ultra-processed foods in the diet of Korean adults and to examine the association between ultraprocessed food consumption and dietary intake and diet quality. | Korean adults (n = 16,657) | cross-sectional | multiple regression models | Korea National Health and Nutrition Examination Survey | 24-h dietary recall | diet (population menas by quintiles of of Energy Contribution of Ultra-processed Foods (Weighted %) | 34 food subgroups | energy (kcal) from UPFs of total diet based on NOVA-> population quintiles based on it | UPF&non-UPF | Korean Healthy Eating Index | The mean reported daily energy intake of Korean adults was 2,031 kcal, 59.8% of which came from unprocessed or minimally processed foods, 4.0% from processed culinary ingredients, 11.2% from processed foods, and 25.1% from ultra-processed foods. The largest contributors to dietary energy intake in the ultra-processed food group were cereals, bread, cakes, and sandwiches (6.4%), distilled alcoholic beverages (2.8%), sugar-sweetened beverages (2.8%), and fish and meat processed foods (2.3%). |
| Siqueira et al., 2021^65^ | Brazil | To develop a nutrient density score and nutrient affordability metrics for 377 most consumed foods in Brazil. | NA | cross-sectional | Descriptive statistics,differences between means with the Bonferroni correction, Sensitivity analyses | Brazilian Institute of Geography and Statistics, retailer web- sites | food composition and consumption data | most consumed foods in Brazil (n = 377) | foods were assigned to seven food groups | classification according to degree of processing (based on NOVA); 1,2,3 and 4 | G1,3&4 | Nutrient Rich Food, affordability (price per 100 g and Cost per 418 kJ) affordability (kcal/R$ and nutrients(NFR)/R$.) | The NOVA categories were unprocessed foods (39 %), processed foods (11 %), ‘ultra-processed’ foods (48 %) and culinary ingredients (2 %). |
| Sneed et al., 2023^66^ | USA | Test the reliability and validity of training coders and assigning Nova categories to individual foods collected via 24-h dietary recalls. | 3-6 years old children (n = 610) | randomized controlled trial | Construct validity was evaluated by comparing the average daily macronutrient content of foods between Nova categories. | data were obtained from the randomized controlled trial of the Growing Right Onto Wellness intervention | 24-h dietary recall | diet level (population mean of target population) | 3497 unique foods, of which 397 were assigned a Nova category, 12 food groups | classification according to degree of processing and kcal contribution (%) to food groups in diet 4 Nova categories (minimally processed, processed culinary ingredients, processed, and ultraprocessed) | G1,3&4 | Healthy eating index | On average, UPFs made up 62% (SD 19) of the day’s calories, and a comparatively high percentage of the day’s added sugar (94%; SD 16) and low percentage of the day’s protein (47%; SD 24) came from UPFs. |
| Spiteri et al., 2018^67^ | Australia | To assess the healthfulness of new food products released into the Australian retail market in 2015, and whether those companies who were Healthier Australia Commitment (HAC) members released healthier food options compared to non-HAC members. | NA | cross-sectional | descriptive statistics and chi-squared tests | new retail food products launched in Australia in 2015 | Mintel’s Global New Products Database | foods (n = 4143) | food groups (n = 17) | classification according to degree of processing; group 1,2, 3 and 4 | G1,2,3&4 | Healthy Choices Framework Victoria (HAC Healthier Australia Commitment members or not), Australian Dietary Guidelines | Overall, 51% of new food products were classified as red, 62% as discretionary and 82% as ultra-processed. |
| Trübwasser et al., 2022^68^ | Ethiopia | The objective of this study was to assess whether food environments in and around schools in urban Ethiopia influence dietary diversity, quality, BMI status or perceptions of adolescents. | 20 pupils aged 15–19 years (n = 217) | cross-sectional | descriptive analysis, multiple linear regression analysis, binary logistic regression | high schools were selected in Addis Ababa (private/government) | 24-h dietary recall | diet (population mean of private and government schools) | categorized into 10 food groups | classification according to degree of processing (based on NOVA); 1,2,3 and 4-> non-UPFs and UPFs | UPF&non-UPF | Minimum Dietary Diversity, the Global Dietary Recommendations scores, use of pocket money, Perceptions Home and School Food Environment, internal and external food environment | The majority of the advertisements (89.9%) were of ultra-processed foods. In contrast, ultra-processed foods and beverages, basically sweets and SSBs, were consumed by almost a quarter (23.5%) of adolescents. |
| Valenzuela et al., 2022^69^ | Chile | To evaluate the quantitative and qualitative differences that exist between the NOVA, Nutri-Score and Chilean Front-of-package (FoP) food warning label according to the Chilean basic food basket list. | NA | cross-sectional | descriptive analysis, principal component analysis, comparative analysis | online database | list of foods belonging to the Chilean basic food basket | foods (n = 736) | food groups (cereals, potatoes and fresh pulses as group 1, dairy products as group 2, fish, meat, eggs and dried pulses as group 3, oils and fats as group 4, and finally sugars as group 5.) | classification according to degree of processing; group 1,2, 3 and 4 | G1,2,3&4 | Nutri-Score and Chilean Front-of-package food warning label | The NOVA classification showed that the most frequent categorization was ultra-processed food (NOVA 4) with 54%, followed by unprocessed (NOVA 1) with 19%. About the FoP warning labels, 57% of the foods were categorized as warning label-free, followed by the category of foods with 3 warning labels (23%). |
| van Dam & Vandevijvere, 2022^70^ | France | To highlight where French food companies are demonstrating leadership in relation to obesity prevention and nutrition, and to identify areas for improvement. In addition, to assess whether stronger nutrition-related commitments translated into stronger practices and performance. | NA | cross-sectional | descriptive statistics, Spearman’s rank correlation coefficient | Euromonitor 2018 market share data of 33 French food companies | Open Food Facts data for France in 2018, Mintel GNPD (Global New Products Database), on brand websites or supermarket websites, national brand websites | food portfolio of companies | product categoires (n =12) | proportion of ultra-processed food products as determined by the NOVA-clas- sification | UPF&non-UPF | Nutri-Score, World Health Organisation Europe nutrient profile model | The median proportion of ultra-processed food products was 63%. |
| van Dam, Reimes & Vandevijvere, 2022^71^ | Belgium | To benchmark and quantitatively assess the nutrition related commitments concerning product formulation, labelling, promotion and accessibility made by the biggest Belgian food and non-alcoholic beverage manufacturers, supermarkets and quick-service restaurants, as well as their practices within these same policy domains. | NA | cross-sectional | Spearman’s rank correlation coefcient | Belgian food and non-alcoholic beverage manufacturers (n=19), supermarkets (n=5) and quick-service restaurants (n=7) | Publicly available commitments and policies from company websites, brand websites, financial and corporate social responsibility reports, industry association websites and media articles were taken into account as well as the abovementioned industry pledges and initiative. | food portfolio of companies | product categoires (n =12) | proportion of ultra-processed food products as determined by the NOVA-clas- sification | UPF&non-UPF | Nutri-score, World Health Organisation Europe nutrient profile model | The median proportion of ultra processed foods was 75% (range = 2%-100%) across product portfolios. |
| Vandevijvere et al., 2020^72^ | Belgium | To assess the monetary cost of diets according to their caloric shares of UPF and MPF for different sociodemographic population groups in Belgium. | Belgian population (n = 3146; 3–64 years) | cross-sectional | descriptive statistics, linear regression | Food Consumption Survey (FCS) 2014–2015 | nonconsecutive 24-hour recalls | diet (population mean by tertiles of UPF and MPF intake %) | no further aggregation | classification according to degree of processing; group 1,2, 3 and 4-> average caloric share (percentage of daily energy intake) of ultraprocessed food products (UPF) and unprocessed/minimally processed foods (MPF)-> UPF and MPF contribution tertiles | G1&G4 | diet cost | The average percentage of daily energy intake from UPF and MPF for the total Belgian population was 29.9 and 21.3%, respectively. There were no significant differences between men and women and between different socio-economic population groups in regard to UPF consumption. Intake of UPF among children was significantly higher compared to adolescents and adults. Individuals with high education level consumed a significantly higher proportion of their daily energy from MPF compared to those with lower education levels. |
| Vellinga et al., 2022^73^ | Netherlands | To investigate nutritional quality, environmental impact and costs of foods and drinks and their consumption in daily diets according to the degree of processing across the Dutch population. | Dutch population (n = 4313) | cross-sectional | descriptive statistics, Mann–Whitney U test or Kruskal–Wallis test for non- normally distributed data and ANOVA for normal dis- tributed,Wilcoxon signed rank test for non-normally distributed data and paired t-test for normal distributed data, sensitivity analysis | Dutch National Food Consumption Survey 2012–2016 | two 24-h non- consecutive dietary recalls | foods, drinks, AND diet (*analyses level were multiple*) | Food groups were based on Globodiet; n = 29 | classification according to degree of processing (group 1, 2,3 and 4), g /total diet/person/day | G1,2,3&4 | nutritional quality, environmental impact (greenhouse gas emissions, blue water use) and cost | Around half to two-thirds of the foods (54%) and drinks (62%) identifed in DNFCS 2012–2016 were categorized as ultra-processed foods (UPF) or drinks (UPD). Approximately a quarter of foods (25%) and one-third of drinks (31%) were classifed as unprocessed or minimally processed foods (MPF) or drinks (MPD). |
| Vellinga et al., 2023^74^ | The Netherlands | To assess the association between levels of UPFD, UPF, and UPD consumption and diet-related environmental impacts and all-cause mortality in Dutch adults. | Dutch adults (n = 38261) | cross-sectional | descriptive statistics, general linear models | Dutch European Prospective Investigation into Cancer and Nutrition cohort | 178-item Food Frequency Questionnaire (FFQ) | diet (poplation mean by quartiles of UPFD, UPF, and UPD consumption in g/1000 kcal) | food group (n = 16) | classification according to degree of processing (based on NOVA); 1,3 and 4->quartiles of UPFD (UP foods and drinks), UPF (UP foods), and UPD (UP drinks) consumption | G1,2,3&4 | diet-related environmental impact indicators: GHG emissions, land use, blue water consumption, acidification, freshwater eutrophication, and marine eutrophication | Energy intake from UPFD (En%), total:: 32 |
| Vergeer et al., 2019^75^ | Canada | To compare the nutritional quality of more- versus less-processed packaged foods and beverages in Canada, using a large, branded food database and two processing classification systems. | NA | cross-sectional | descriptive statistics, linear regression | University of Toronto FLIP 2017 database | nutritional information | food products (n = 17269) | Food groups (n = 22) | classification according to degree of processing; group 1,2, 3 and 4 | G1,2,3&4 | Poti et al. processing classification systems, nutritional quality | More than two-thirds of the sampled products were deemed ultra- or highly processed according to the NOVA and Poti et al. systems. Most packaged food and beverage products were classified as ultra-processed or highly processed according to the NOVA (73.5%) and Poti et al. (68.3%) systems, respectively. Fewer products were considered unprocessed or minimally processed (12.7%), processed (10.6%) or processed culinary ingredient (3.1%) under NOVA. Based on the Poti et al. system, 52.2% were highly processed stand-alone products, 13.1% were highly processed ingredients, 16.3% were moderately processed, 9.1% were basic processed and 6.3% were unprocessed/minimally processed. |
| Vicente et al., 2023^76^ | Brazilia | To (i) identify the dietary quality of community-dwelling older adults using three approaches; two conventional a priori indexes and a ratio between minimally processed and ultra-processed foods (UPR; based on the NOVA classification); (ii) explore some differences and similarities among the approaches; (iii) investigate the association of DII and MDS with a biomarker of systemic inflammation, and investigate if UPR could be used in a complementary way to these associations. | independent (non-frail) older adults, without any inflammatory disease (n = 73) | cross-sectional | Kolmogorov-Smirnov test, Pearson's correlation analyses and the regression models | data from a clinical study | 24-h dietary recall | diet (population mean of target population) | MedDiet food components (n = 8) | classification according to degree of processing (based on NOVA); 1,2,3 and 4->ratio between unprocessed or minimally processed/ultra-processed food (UPR) | UPF&non-UPF | dietary inflammatory index, Mediterranean Diet Scale | median intake (%): 0.29 (0.0-1.3) |
| Vogliano et al., 2021^77^ | Three Geographically Distinct Solomon Islands Sites (Melanesia, Pacific Islands) | To assess nutrition transitions and diet quality by comparing three geographically unique rural and urban indigenous Solomon Islands populations. | Indigenous Solomon Islanders | cross-sectional | descriptive statistics, linear regression models | observational mixed-method cross-sectional study | repeat 24-h multiple-pass recall | diet (population means of rural costal, rural inland and urban) | NA | classification according to degree of processing; group 1,2, 3 and 4->UPF contribution to total diet in proportion (%) | UPF&non-UPF | diet diversity, traditional food intake, takeout food intake, WHO recommendations of >400 g of non-starchy fruits and vegetables daily (NSFV) | overall average (of 3 areas): 12.13% |

References:

S1 Abreu S, Liz Martins M. Cross-Classification Analysis of Food Products Based on Nutritional Quality and Degree of Processing. Nutrients. 2023;15(14):3117. doi:10.3390/nu15143117.

S2 Aceves-Martins M, Bates RL, Craig LC, et al. Nutritional quality, environmental impact and cost of ultra-processed foods: a UK food-based analysis. Int. J. Environ. Res. Public Health. 2022;19(6):3191. doi:10.3390/ijerph19063191

S3 Angelino D, Dinu M, Gandossi B, et al. Processing and nutritional quality of breakfast cereals sold in Italy: results from the Food Labelling of Italian Products (FLIP) Study. Nutrients. 2023;15(8):2013. doi:10.3390/nu15082013

S4 Baldridge AS, Huffman MD, Taylor F, et al. The healthfulness of the US packaged food and beverage supply: a cross-sectional study. Nutrients. 2019;11(8):1704. doi:10.3390/nu11081704

S5 Barrett EM, Gaines A, Coyle DH, et al. Comparing product healthiness according to the Health Star Rating and the NOVA classification system and implications for food labelling systems: An analysis of 25 486 products in Australia. Nutr Bull. 2023;48(4):523-534. doi:10.1111/nbu.12640. Epub 2023 Oct 28

S6 Batal M, Johnson-Down L, Moubarac JC, et al. Quantifying associations of the dietary share of ultra-processed foods with overall diet quality in First Nations peoples in the Canadian provinces of British Columbia, Alberta, Manitoba and Ontario. Public Health Nutr. 2018;21(1):103-113. doi:10.1017/S1368980017001677

S7 Batista CHK, Leite FHM, Borges CA. Association between advertising patterns and ultra-processed food in small markets. Ciência & Saúde Coletiva, 2022;27:2667-2678. doi:10.1590/1413-81232022277.19122021.

S8 Baye K, Yaregal Z. The Global Diet Quality Score predicts diet quality of women of reproductive age in Addis Ababa, Ethiopia. BJN. 2023;130:1573-1579. doi:10.1017/S0007114523000508.

S9 Berardy A, Fresán U, Matos RA, et al. Environmental impacts of foods in the Adventist health study-2 dietary questionnaire. Sustainability. doi:2020;12:10267. doi:10.3390/su122410267

S10 Blanchet R, Willows N, Johnson S, et al. Traditional food, health, and diet quality in Syilx Okanagan adults in British Columbia, Canada. Nutrients. 2020;12:927. doi:10.3390/nu12040927

S11 Bleiweiss-Sande R, Chui K, Evans EW, et al. Robustness of Food Processing Classification Systems. Nutrients. 2019;11:1344. doi:10.3390/nu11061344.

S12 Bonaccio M, Di Castelnuovo A, Ruggiero E, et al. Joint association of food nutritional profile by Nutri-Score front-of-pack label and ultra-processed food intake with mortality: Moli-sani prospective cohort study. BMJ. 2022,378. doi:10.1136/bmj-2022-070688

S13 Braesco V, Souchon I, Sauvant P, et al. Ultra-processed foods: how functional is the NOVA system?. Eur. J. Clin. Nutr. 2022;76(9):1245-1253. doi:10.1038/s41430-022-01099-1

S14 Cediel G, Reyes M, Corvalán C, et al. Ultra-processed foods drive to unhealthy diets: evidence from Chile. Public Health Nutr. 2021;24(7):1698-1707. doi:10.1017/S1368980019004737

S15 Chen YC, Huang YC, Lo YTC, et al. Secular trend towards ultra-processed food consumption and expenditure compromises dietary quality among Taiwanese adolescents. Food Nutr Res. 2018,62. doi:10.29219/fnr.v62.1565

S16 Cooper SL, Pelly FE, Lowe JB. Assessment of the construct validity of the Australian Health Star Rating: a nutrient profiling diagnostic accuracy study. Eur. J. Clin. Nutr. 2017;71(11):1353-1359. doi:10.1038/ejcn.2017.23

S17 da Rocha BRS, Rico-Campà A, Romanos-Nanclares A, et al. Adherence to Mediterranean diet is inversely associated with the consumption of ultra-processed foods among Spanish children: The SENDO project. Public Health Nutr. 2021;24:3294-3303. doi:10.1017/S1368980020001524

S18 da Silva JT, Garzillo JMF, Rauber F, et al. Greenhouse gas emissions, water footprint, and ecological footprint of food purchases according to their degree of processing in Brazilian metropolitan areas: a time-series study from 1987 to 2018. Lancet Planetary Health. 2021,5:775-785.

S19 Davidou S, Christodoulou A, Fardet A, Frank K. The holistico-reductionist Siga classification according to the degree of food processing: an evaluation of ultra-processed foods in French supermarkets. Food Funct. 2020;11(3):2026-2039. doi:10.1039/C9FO02271F

S20 de Las Heras-Delgado S, Shyam S, Cunillera È, et al Are plant-based alternatives healthier? A two-dimensional evaluation from nutritional and processing standpoints. Food Res Int. 2023;169:112857. doi:10.1016/j.foodres.2023.112857

S21 de Moraes MM, Oliveira B, Afonso C, et al. An ultra-processed food dietary pattern is associated with lower diet quality in Portuguese adults and the elderly: The UPPER project. Nutrients. 2021;13:4119. doi:10.3390/nu13114119.

S22 Delgado-Rodríguez R, Moreno-Padilla M, Moreno-Domínguez S, Cepeda-Benito A. Food addiction correlates with emotional and craving reactivity to industrially prepared (ultra-processed) and home-cooked (processed) foods but not unprocessed or minimally processed foods. Food Qual Prefer. 2023;110:104961. doi:10.1016/j.foodqual.2023.104961

S23 Derbyshire, E. Are all ‘ultra-processed’foods nutritional demons? A commentary and nutritional profiling analysis. Trends Food Sci Technol. 2019;94:98-104. doi:10.1016/j.tifs.2019.08.023

S24 Detopoulou P, Dedes V, Pylarinou I, et al. Dietary acid load is associated with waist circumference in university students with low adherence to the Mediterranean diet: The potential role of ultra-processed foods. Clin Nutr ESPEN. 2023;56:43-51. doi:10.1016/j.clnesp.2023.05.005

S25 Dickie S, Woods J, Machado P, Lawrence M. Nutrition classification schemes for informing nutrition policy in Australia: nutrient-based, food-based, or dietary-based?. Curr Dev Nutr. 2022;6(8):112. doi:10.1093/cdn/nzac112

S26 Dinu M, Tristan Asensi M, Pagliai G, et al. Consumption of ultra-processed foods is inversely associated with adherence to the Mediterranean diet: a cross-sectional study. Nutrients. 2022;14:2073. doi:10.3390/nu14102073

S27 Estell ML, Barrett EM, Kissock KR, et al. Fortification of grain foods and NOVA: the potential for altered nutrient intakes while avoiding ultra-processed foods. Eur J Nutr. 2022;1-11. doi:10.1007/s00394-021-02701-1

S28 Fardet A, Rock E. How to protect both health and food system sustainability? A holistic ‘global health’-based approach via the 3V rule proposal. Public Health Nutr. 2020;23:3028-3044. doi:10.1017/S136898002000227X

S29 Fardet A, Méjean C, Labouré H, et al. The degree of processing of foods which are most widely consumed by the French elderly population is associated with satiety and glycemic potentials and nutrient profiles. Food Funct. 2017;8:651-658. doi:10.1039/c6fo01495j

S30 Gallegos-Riofrío CA, Waters WF, Carrasco A, et al. Caliata: an Indigenous Community in Ecuador offers lessons on food sovereignty and sustainable diets. Curr Dev Nutr. 2021;5:61-73. doi:10.1093/cdn/nzab009

S31 García S, Pastor R, Monserrat-Mesquida M, et al. Ultra-processed foods consumption as a promoting factor of greenhouse gas emissions, water, energy, and land use: A longitudinal assessment. Sci Total Environ. 2023;891:164417. doi:10.1016/j.scitotenv.2023.164417

S32 Garzillo JMF, Poli VFS, Leite FHM, et al. Ultra-processed food intake and diet carbon and water footprints: a national study in Brazil. Revista de saude publica, 2022;56:6. doi:10.11606/s1518-8787.2022056004551

S33 Grech A, Rangan A, Allman-Farinelli M, et al. A Comparison of the Australian Dietary Guidelines to the NOVA Classification System in Classifying Foods to Predict Energy Intakes and Body Mass Index. Nutrients. 2022;14:3942. doi:10.3390/nu14193942

S34 Gupta S, Hawk T, Aggarwal A, Drewnowski A. Characterizing ultra-processed foods by energy density, nutrient density, and cost. Front Nutr. 2019;6:454858. doi:10.3389/fnut.2019.00070

S35 Gupta S, Rose CM, Buszkiewicz J, et al. Characterising percentage energy from ultra-processed foods by participant demographics, diet quality and diet cost: Findings from the Seattle Obesity Study (SOS) III. BJN. 2021;126:773-781. doi:10.1017/S0007114520004705

S36 Hallinan S, Rose C, Buszkiewicz J, Drewnowski A. Some ultra-processed foods are needed for nutrient adequate diets: linear programming analyses of the Seattle obesity study. Nutrients. 2021;13:3838. doi:10.3390/nu13113838

S37 Hässig A, Hartmann C, Sanchez-Siles L, Siegrist M. Perceived degree of food processing as a cue for perceived healthiness: the NOVA system mirrors consumers’ perceptions. Food Qual Prefer. 2023;110:104944. doi:10.1016/j.foodqual.2023.104944

S38 Julia C, Baudry J, Fialon M, et al. Respective contribution of ultra-processing and nutritional quality of foods to the overall diet quality: results from the NutriNet-Santé study. Eur J Nutr. 2023;62:157-164. doi:10.1007/s00394-022-02970-4

S39 Juul F, dos Santos Simões B, Litvak J, et al. Processing level and diet quality of the US grocery cart: is there an association?. Public Health Nutr. 2019;22:2357-2366. doi:10.1017/S1368980019001344

S40 Juul F, Lin Y, Deierlein AL, et al. Trends in food consumption by degree of process. BJN. 2021;126:1861-1871. doi:10.1017/S000711452100060X

S41 Kesse-Guyot E, Allès B, Brunin J, et al. Environmental impacts along the value chain from the consumption of ultra-processed foods. Nature Sustainability. 2023;6(2):192-202. doi:10.1038/s41893-022-01013-4

S42 Lavigne-Robichaud M, Moubarac JC, Lantagne-Lopez S, et al. Diet quality indices in relation to metabolic syndrome in an Indigenous Cree (Eeyouch) population in northern Québec, Canada. Public Health Nutr. 2018;21(1):172-180. doi:10.1017/S136898001700115X

S43 Liu J, Steele EM, Li Y, et al. Consumption of ultraprocessed foods and diet quality among US children and adults. Am J Prev Med. 2022;62(2):252-264. doi:10.1016/j.amepre.2021.08.014

S44 Maia EG, Passos CMD, Granado FS, et al. Replacing ultra-processed foods with fresh foods to meet the dietary recomendations: a matter of cost?. Cadernos de Saúde Pública. 2022;37:e00107220. doi:10.1590/0102-311X00107220

S45 Marchese L, Livingstone KM, Woods JL, et al. Ultra-processed food consumption, socio-demographics and diet quality in Australian adults. Public Health Nutr. 2022;25(1):94-104. doi:10.1017/S1368980021003967

S46 Martinez-Perez N, Arroyo-Izaga M. Availability, nutritional profile and processing level of food products sold in vending machines in a Spanish public university. Int. J. Environ. Res. Public Health. 2021;18(13):6842. doi:10.3390/ijerph18136842

S47Martinez-Perez C, San-Cristobal R, Guallar-Castillon P, et al. Use of different food classification systems to assess the association between ultra-processed food consumption and cardiometabolic health in an elderly population with metabolic syndrome (PREDIMED-Plus Cohort). Nutrients. 2021;13(7):2471. doi:10.3390/nu13072471.

S48 Martinez-Perez C, Daimiel L, Climent-Mainar C, et al. Integrative development of a short screening questionnaire of highly processed food consumption (sQ-HPF). Int J Behav Nutr Phys Act. 2022;19(1):6. doi:10.1186/s12966-021-01240-6

S49 Mendes C, Miranda L, Claro R, Horta P. Food marketing in supermarket circulars in Brazil: An obstacle to healthy eating. Preventive Med Reports. 2021;21:101304. doi:10.1016/j.pmedr.2020.101304

S50 Mendoza-Velázquez A, Lara-Arévalo J, Siqueira KB, et al. Affordable nutrient density in brazil: nutrient profiling in relation to food cost and NOVA category assignments. Nutrients. 2022;14(20):4256. doi:10.3390/nu14204256

S51 Mignogna C, Costanzo S, Di Castelnuovo A, et al. The inflammatory potential of the diet as a link between food processing and low-grade inflammation: An analysis on 21,315 participants to the Moli-sani study. Clin Nutr. 2022;41:2226-2234. doi:10.1016/j.clnu.2022.08.020

S52 Morales FJ, Mesías M, Delgado-Andrade C. Association between heat-induced chemical markers and ultra-processed foods: A case study on breakfast cereals. Nutrients. 2020;12:1418. doi:10.3390/nu12051418

S53 Otten JJ, Buszkiewicz J, Tang W, et al. The impact of a city-level minimum-wage policy on supermarket food prices in Seattle-King County. Int. J. Environ. Res. Public Health. 2017;14:1039. doi:10.3390/ijerph14091039

S54 Phulkerd S, Dickie S, Thongcharoenchupong N, et al. Choosing an effective food classification system for promoting healthy diets in Thailand: a comparative evaluation of three nutrient profiling-based food classification systems (government, WHO, and Healthier Choice Logo) and a food-processing-based food classification system (NOVA). Front Nutr. 2023;10:1149813. doi:10.3389/fnut.2023.1149813

S55 Phulkerd S, Thongcharoenchupong N, Dickie S, et al. Profiling ultra-processed foods in Thailand: sales trend, consumer expenditure and nutritional quality. Global Health. 2023;19:64. doi:10.1186/s12992-023-00966-1

S56 Pulker CE, Trapp GS, Scott JA, Pollard CM. Alignment of supermarket own brand foods’ front-of-pack nutrition labelling with measures of nutritional quality: An Australian perspective. Nutrients. 2018;10:1465. doi:10.3390/nu10101465

S57 Rizzolo-Brime L, Orta-Ramirez A, Puyol Martin Y, Jakszyn P. Nutritional assessment of plant-based meat alternatives: a comparison of nutritional information of plant-based meat alternatives in Spanish supermarkets. Nutrients. 2023;15:1325. doi:10.3390/nu15061325

S58 Robert M, Shankland R, Bellicha A, et al. Associations between resilience and food intake are mediated by emotional eating in the NutriNet-Santé Study. Journal of Nutr. 2022;152:1907-1915. doi:10.1093/jn/nxac124

S59 Rodrigues VM, Rayner M, Fernandes AC, et al. Nutritional quality of packaged foods targeted at children in Brazil: which ones should be eligible to bear nutrient claims?. Int J Obes. 2017;41:71-75. doi:10.1038/ijo.2016.167

S60 Romero Ferreiro C, Lora Pablos D, Gómez de la Cámara A. Two dimensions of nutritional value: Nutri-Score and NOVA. Nutrients. 2021;13: 2783. doi:10.3390/nu13082783

S61 Rossato SL, Khandpur N, Lo CH, et al. Intakes of unprocessed and minimally processed and Ultraprocessed food are associated with diet quality in female and male health professionals in the United States: A prospective analysis. J Acad Nutr Diet. 2023;123:1140-1151. doi:10.1016/j.jand.2023.03.011

S62 Ruggiero E, Esposito S, Costanzo S, et al. Ultra-processed food consumption and its correlates among Italian children, adolescents and adults from the Italian Nutrition & Health Survey (INHES) cohort study. Public Health Nutr. 2021;24:6258-6271. doi:10.1017/S1368980021002767

S63 Salomé M, Arrazat L, Wang J, et al. Contrary to ultra-processed foods, the consumption of unprocessed or minimally processed foods is associated with favorable patterns of protein intake, diet quality and lower cardiometabolic risk in French adults (INCA3). Eur J Nutr. 2021;60:4055-4067. doi:10.1007/s00394-021-02576-2

S64 Shim JS, Shim SY, Cha HJ, et al. Association between ultra-processed food consumption and dietary intake and diet quality in Korean adults. JAcad Nutr Diet. 2022;122:583-594. doi:10.1016/j.jand.2021.07.012

S65 Siqueira KB, Borges CA, Binoti ML, et al. Nutrient density and affordability of foods in Brazil by food group and degree of processing. Public Health Nutr. 2021;24(14):4564-4571. doi:10.1017/S1368980020004358

S66 Sneed NM, Ukwuani S, Sommer EC, et al. Reliability and validity of assigning ultraprocessed food categories to 24-h dietary recall data. Am J Clin Nutr. 2023;117:182-190. doi:10.1016/j.ajcnut.2022.10.016.

S67 Spiteri SA, Olstad DL, Woods JL. Nutritional quality of new food products released into the Australian retail food market in 2015–is the food industry part of the solution? BMC Public Health, 2018;18:1-10. doi:10.1186/s12889-018-5127-0

S68 Trübswasser U, Talsma EF, Ekubay S, et al. Factors influencing adolescents' dietary behaviors in the school and home environment in Addis Ababa, Ethiopia. Front Public Health. 2022;10:861463. doi:10.3389/fpubh.2022.861463

S69 Valenzuela A, Zambrano L, Velásquez R, et al. Discrepancy between food classification systems: Evaluation of Nutri-Score, NOVA classification and chilean front-of-package food warning labels. Int. J. Environ. Res. Public Health. 2022,19:14631. doi:10.3390/ijerph192214631

S70 Van Dam I, Vandevijvere S. Benchmarking the nutrition-related commitments and practices of major French food companies. BMC Public Health, 2022;22:1435. doi:10.1186/s12889-022-13780-y

S71 Van Dam I, Reimes N, Vandevijvere S. Benchmarking the nutrition-related commitments and practices of major Belgian food companies. Int J Behav Nutr Phys Act. 2022;19:43. doi:10.1186/s12966-022-01269-1

S72 Vandevijvere S, Pedroni C, De Ridder K, Castetbon, K. The cost of diets according to their caloric share of ultraprocessed and minimally processed foods in Belgium. Nutrients. 2020;12:2787. doi:10.3390/nu12092787

S73 Vellinga RE, van Bakel M, Biesbroek S, et al. Evaluation of foods, drinks and diets in the Netherlands according to the degree of processing for nutritional quality, environmental impact and food costs. BMC Public Health. 2022;22: 877. doi:10.1186/s12889-022-13282-x

S74 Vellinga RE, van den Boomgaard I, Boer JM, Different Levels of Ultraprocessed Food and Beverage Consumption and Associations with Environmental Sustainability and All-cause Mortality in EPIC-NL. Am J Clin Nutr. 2023;118(1):103-113. doi:10.1016/j.ajcnut.2023.05.02Siqueira KB, Borges CA, Binoti ML, et al. Nutrient density and affordability of foods in Brazil by food group and degree of processing. Public Health Nutr. 2021;24(14):4564-4571. doi:10.1017/S1368980020004358

S75 Vergeer L, Veira P, Bernstein JT, et al. The calorie and nutrient density of more-versus less-processed packaged food and beverage products in the Canadian food supply. Nutrients. 2019;11:2782. doi:10.3390/nu11112782

S76 Vicente B M, Almeida Bastos A, de Melo CM, et al. Correlation Between Different Dietary Indexes, and Their Association with An Anti-inflammatory Biomarker in Older Adults: An Exploratory Study. Eur J Geriatr Geront. 2023;5(3). doi:10.4274/ejgg.galenos.2023.2022-10-5

S77 Vogliano C, Raneri JE, Maelaua J, et al. Assessing diet quality of indigenous food systems in three geographically distinct solomon islands sites (Melanesia, Pacific Islands). Nutrients. 2020;13:30. doi:10.3390/nu13010030
