# Supplementary material for: Fifteen Years of NOVA Food-Processing Classification: “Friend or Foe” Among Sustainable Diet Indicators? A Scoping Review
Source: Nutr Rev. 2025 Jan 23;83(4):771–91. doi: 10.1093/nutrit/nuae207 (PMC11894255; doi:10.1093/nutrit/nuae207)
Supplement: nuae207_Supplementary_Data [file nuae207_supplementary_data.zip › nuae207_Supplementary_Data/Table_S2_Identified_associations_between_NOVA_system_and_other_SDIs[AU].docx]

| Reference | Identified association between NOVA and other sustainable diet indicators in the reviewed studies |
| --- | --- |
| Abreu & Martins, 2023^1^ | - 73.7% of UPF were classified as Nutri-Score C, D, and E - 22.9% of products in NOVA 4 group had high sugar content - NOVA 3 and 4 had a higher proportion of products with high salt content - NOVA classification negatively correlates with the Multiple Traffic Lights system for total fat - NOVA classification positively correlates with the Multiple Traffic Lights system for total sugar and salt |
| Aceves-Martins et al., 2022^2^ | - On a per 100 kcal basis, ultra-processed and processed foods had a lower nutritional quality, lower greenhouse gas emissions, and were cheaper than minimally processed foods, regardless of their total fat, salt, and/or sugar content. - The most nutritious, environmentally friendly, and affordable foods were generally lower in total fat, salt, and sugar, irrespective of processing level. |
| Angelino et al., 2023^3^ | - The NOVA 4 products showed the highest energy, total fat, saturates, and sugar content per 100 g and had the highest number of items with Nutri-Score C (49%) and D (22%). Conversely, NOVA 1 products had the highest content of fiber and protein, the lowest amounts of sugars and salt, and 82% of them were Nutri-Score A, while few Nutri-Score B and C were found. - Differences were attenuated when products were compared for their NutrInform battery, with NOVA 4 items showing only slightly fuller batteries for saturated fats, sugar, and salt than NOVA 1 and NOVA 3 products - The score plot in Figure 3B confirms a high variability that did not allow products to be grouped based on the NOVA and Nutri-Score values. - On the whole, most of the products with B, C, and D Nutri-Scores were described as having high energy, total carbohydrates, salt, total and saturated fats, and sugars, with no distinctions for NOVA groups. - Then, a main characterization of the A products by fiber and protein was slightly evidenced, but no distinction among NOVA groups could be pointed out. - Data confirmed the evidence of a tight variability for daily energy contribution of a 30 g serving of the products, from 5 to 7%, with few distinctions among the NOVA groups. On the contrary, most of the variability referred to the contribution to the daily amounts of total and saturated fats, but just for a few typologies. |
| Baldridge et al., 2019^4^ | - Healthfulness and processing varied widely by category and manufacturer - The overall mean proportion of products considered ultra-processed was 71% - The level of processing did not always correlate with healthiness using the HSR - Seventy-one percent of products were classified as ultra-processed using the NOVA classification - Forty percent of products had a Health Star Rating (HSR) of 3.5 or higher |
| Barrett et al., 2023^5^ | - Fair agreement between systems in classifying products as ‘healthier’ (HSR ≥3.5 or NOVA group 1–3) or ‘less healthy’ (HSR <3.5 or NOVA group 4); approximately one-third of products were discordant in classification - NOVA group 4 foods had significantly lower healthy star ratings compared to NOVA group 1–3 products - 34.3% of NOVA group 4 products had a healthy star rating of ≥3.5 - Convenience foods had the highest relative percentage of discordant products - The most common discordant products that were in NOVA group 4 but HSR ≥3.5 were convenience foods, sports/diet foods (foods for specific dietary uses), meat alternatives, and cereal and grain products. |
| Batal et al., 2018^6^ | - The UPF fraction of the diet had significantly lower levels of vitamin A, K, and protein compared to nonUPF fraction and had higher levels of free sugars and sodium compared to nonUPF fraction - Increased UPF contribution led to higher overall intakes of energy, carbohydrates, free sugar, saturated fat, sodium, calcium, and vitamin C and lower intakes of protein, fiber, potassium, iron, and vitaminA - Diets with traditional First Nations food were lower in UPF |
| Batista et al., 2022^7^ | - More than 95% of the ultra-processed foods had at least 1 critical nutrient in excess. |
| Baye & Yaregal, 2023^8^ | - Consumption of UPF was inversely associated with GDQS. Wealth score was, however, not associated with any of the GDQS scores but was weakly associated with MDD-W - Multiple linear regression model exploring the association of various factors with GDQS. The association between GDQS and WDDS, snacking, skipping breakfast, eating-out and UPF remained significant. The association between GDQS+ and WDDS, as well as that of GDQS- and UPF remained significant and strong. |
| Berardy et al., 2020^9^ | - Different levels of processing result in varied environmental impacts; Per serving, ultra-processed foods had the highest GWP, processed foods had thehighest land use, and minimally processed foods the highest water consumption. - Meat and ultra-processed foods have the worst environmental impacts - Processing can significantly increase the overall environmental impact, especially for plant-based products |
| Blanchet et al., 2020^10^ | - TF eaters also had significantly better diet quality based on the HEI-C and the %energy from UPP - Participants who ate TF had a lower %E from UPP than participants who did not eat TF, an indicator of better diet quality. TF eaters also had higher %E from fresh and minimally processed foods - Adjusted for covariates, the average dietary cost per 2000 kcal was significantly lower for individuals in the highest compared to the lowest tertile for the proportion of daily energy consumed from UPF, and significantly higher for individuals in the highest compared to the lowest tertile for the proportion of daily energy consumed from MPF |
| Bleiweiss-Sande et al., 2019^11^ | - There was a moderate overall agreement between classification systems (0.41< kappa > 0.60) - High agreement was between Nova and IFIC systems (70.0%) and between Nova and UNC systems (76.0%) - Added sugars and sodium had the lowest mean concentrations in category 1 foods and highest in category 3 foods for all systems |
| Bonaccio et al., 2022^12^ | - Standard deviations of ultra-processed foods are more than four times larger than that for the FSAm-NPS dietary index - The correlation between FSAm-NPS dietary index and ultra-processed food intake is low to moderate (Spearman correlation coefficient=0.34) - Higher levels of FSAm-NPS dietary index or ultra-processed food intake were inversely associated with adherence to a Mediterranean diet, monounsaturated-to-saturated fat ratio, and consumption of fruits and nuts, vegetables, cereals, legumes, fish, alcohol, starch, fiber, and protein. Increases in both scores positively correlated with energy intake, total fat, saturated fat, polyunsaturated fat, and dietary cholesterol - We observed divergent associations for meats, which were inversely associated with ultra-processed food intake but not with the FSAm-NPS dietary index, and for milk and dairy products, which were directly correlated only with ultra-processed food intake. - Energy from carbohydrates decreases with the FSAm-NPS dietary index but increases with ultra-processed food intake - Monounsaturated fatty acids increased with ultra-processed food intake, and sodium was positively associated with the FSAm-NPS dietary index and inversely with ultra-processed food intake |
| Braesco et al., 2022^13^ | - Both NOVA4 marketed and generic foods were distributed across all nutrient profiles. - NOVA3-marketed foods had higher nutritional quality than NOVA4 based on Nutri-Score, SAIN, and LIM values. - For marketed foods, no significant difference in energy density or NRF 9.3 values between NOVA3 and NOVA4 foods. - NOVA1 generic foods had higher nutritional quality than NOVA3, and NOVA3 had higher quality than NOVA4 - NOVA2 foods consistently displayed the worst nutritional quality. |
| Cediel et al., 2021^14^ | - There was a positive association with NCD-promoting nutrients like free sugars and total fats, while negative association with NCD-protective nutrients such as K and fiber with the dietary share of ultra-processed foods , the content of Na presented no significant association. - Higher consumption of ultra-processed foods leads to increased prevalence of nutrient inadequacy, vica-versa, reducing ultra-processed food consumption could significantly reduce nutrient inadequacy |
| Chen et al., 2018^15^ | - Consuming more ultra-processed foods (UPFs) is associated with lower protein energy intake and poorer dietary quality. - Participants who consumed more UPFs had the lowest proportions of protein-energy intake in both surveys; while those who consumed more UPFs had higher levels of saturated fat and lower levels of monounsaturated and polyunsaturated fat, dietary fiber, and micronutrient intakes (vitamins A, CD, B-1, and B-6, as well as niacin, potassium, magnesium, calcium, and iron) - The participants who consumed more UPFs and fewer original foods exhibited poorer dietary quality. - Increased UPF energy intake or expenditure is linked to a higher risk of poor dietary quality |
| Cooper et al., 2017^16^ | - The dairy beverages model can distinguish between healthy and less healthy options - 37% of ultra-processed dairy foods have a rating of 4 stars or above - 33% of non-ultra-processed dairy have a rating of 2 stars or below - The study highlights differences in health ratings between ultra-processed and non-ultra-processed dairy - The median HSR for all dairy products was 3.5 stars - The HSR showed discrimination power for dairy beverages but not for yogurt and cheeses - A cutoff value of four stars demonstrated high sensitivity for identifying healthy dairy beverages |
| da Rocha et al., 2021^17^ | - 32·2% of total energy intake came from UPF - Each two additional points in the KIDMED score was associated with 31 % lower energy intake from UPF. - We also found that 71·6 % of the variability in free sugar intake was explained by the variability in UPF consumption - Two additional points in the - KIDMED scores were associated with a 28 % lower contribution of UPF to the Total Energy Intake (TEI) in the crude model. - A linear trend observed for UPF contribution to total energy intake across MedDiet adherence categories |
| da Silva et al., 2021^18^ | - The effects of ultra-processed foods on GHGE, water footprint, and ecological footprint all increased during the study period. The types of G4 foods contributing the largest proportions to the environmental impact varied across the indicators and the years, with some exceptions. - Between 1987–88 and 2017–18, diet-related GHGE, water footprint, and ecological footprint increased by 21%, 22%, and 17% respectively - No change in environmental indicators for G1 foods over time |
| Davidou et al., 2020^19^ | - Siga technological groups, based on holistic NOVA food groups, distinguish four new subgroups based on more specific and reductionist criteria. |
| de Las Heras-Delgado et al., 2023^20^ | - 68% of PBAPs, 43% of processed, and 75% of unprocessed animal homologs had Nutri-Score ratings of A or B. - 17% of PBAPs, 35% of processed, and 13% of unprocessed animal-based food were in Nutri-Score categories D or E. - Dairy, fish, and meat alternatives had lower FSAm-NPS scores, while cheese alternatives scored higher. |
| de Moraes et al., 2021^21^ | - Being younger and having a lower intake of dietary fiber and vitamins were associated with an 'Unhealthy' dietary pattern - "Unhealthy" DP followers had a higher total energy intake and contributions of UPF, carbohydrates, saturated fats, and free sugars, and a lower contribution of unprocessed or minimally processed foods, processed culinary ingredients, and protein, as well as a lower intake of alcohol, dietary fiber, vitamin A, vitamin C, folates, sodium, potassium, magnesium and iron, and a slightly lower energy density than those following the "Traditional" DP |
| Delgado-Rodríguez et al., 2023^22^ | - Our findings suggest that both UP (NOVA 4) and P (NOVA 3) foods have a greater addictive potential than MP (NOVA 1) foods. We also found clear evidence showing that UP (NOVA 4) foods have greater addictive potential than P (NOVA 3) foods. |
| Derbyshire, 2019^23^ | - The number of ingredients listed was, on average 13.6 but ranged from 5 to 33. Forty percent of the UPFs contained ten ingredients or less. Just under half (44 percent) had 5 to 10 ingredients sodium, fiber or protein) nor nutrient profiling scores listed. These findings demonstrate a large degree of variability in the number of ingredients used between products. Quite often some of the ingredients used were herbs and spices, preservatives, or fortificants. This raises the question of whether it is fair to judge products based on ‘ingredient numbers’ when some ingredients listed have a functional role to play by improving safety, shelf-life, and nutritional profile. It is also unclear why a cut-off of 5 ingredients or more has been used for the NOVA definition of UPFs - Spearman Rank correlations between the number of ingredients and individual nutritional components were also not statistically significant:(3)Previously it has been reported that UPF consumption has a 'protein-diluting effect' – displacing protein from diets whilst driving up energy intakes. The present commentary refutes this as 30 percent of the identified UPFs were a 'source' of protein according to European Nutrition Claims. Of the 50 UPFs identified 28 percent provided fortificants or other functional components. One in five (20 percent) were fortified with riboflavin, vitamin B6, or calcium, 18 percent with vitamin D, 14 percent added vitamin B12, 12 percent were fortified with thiamine, niacin, or iron, 10 percent were fortified with folic acid, 6 percent provided vitamin E, 4 percent were fortified with iodine or pantothenic acid and 2 percent had zinc or magnesium added. Omega-3 fatty acids were added in 6 percent of UPFs and plant stanols in 4 percent of UPF products |
| Detopoulou et al., 2023^24^ | - Spearman correlation coefficients between PRAL, NEAP food groups, MedDietScore, and UPF consumption were analyzed; as can be seen, significant positive associations between Dietary acid load (DAL) indices and sodas, alcohol, meat, fish, refined grains, sweets, fast foods, and UPF intake were documented. |
| Dickie et al., 2022^25^ | - NOVA has a slight agreement with ADG, HSR, Nutri-Score, and Chilean NPM while fair with WHO-Euro and moderate with PAHO. - Regarding the advantages-disadvantageous food as binary classification, NOVA (non-UPFs) has a high agreement with ADG healthy food groups, HSR "healthy" food, and Nutri-score A-C rating, while high disagreement with PAHO and WHO-Euro "unhealthy" food classification. |
| Dinu et al., 2022^26^ | - A significant inverse association was found between the Medi-Lite score and the percentage of ultra-processed food (UPF) in the diet - Association between MD adherence and UPF consumption observed in both women and men - Higher UPF intake was also linked to increased consumption of meat, fats, seasonings, and sugary products - Increased UPF intake was associated with lower intake of MD's typical products like fruits, vegetables, nuts, and fish |
| Estell et al., 2021^27^ | - In the case of modeled intake of key nutrients when UPF were excluded, specifically, thiamin, folate, and iodine decreased, as substitutions are rarely fortified. - Diets with no UPF, where substitutes are carefully chosen, have the potential to meet Nutrient Reference Values, but deviation from customary food choices may mean adoption of substitutes is unlikely - Statistically significant differences were found in all nutrients, including whole grain, dietary fiber and food components of interest observed when UPF were excluded (p < 0.05) |
| Fardet & Rock, 2020^28^ | - The study identifies three main dimensions for food system sustainability: Végétal (plant), Vrai (real), and Varié (varied) while organic, local, and/or seasonal should be preferred - The three rules are interconnected, and excluding one dimension would impair both human health and food system sustainability: no single dimension can be excluded without impacting human health and sustainability. |
| Fardet et al., 2017^29^ | - ANOVA and correlation analyses show that more processed food has lower satiety potential and nutrient density and higher glycemic impact especially when comparing G1 (MPF) and G3 (UPF) - Technological group 1 has significantly higher NDS compared to groups 2 (PF) and 3 with around a 4-fold diﬀerence - LIM values significantly differ within each group, with the highest mean value in group 3 - Group 1 has the highest mean Fullness Factor (FF) value and the lowest in Group 3 |
| Gallegos-Riofrío et al., 2021^30^ | - Statistical models show significant correlations between agroecological richness, land size, and healthy diet index (NOVA-based) - Healthy diet index (NOVA-based) is associated with increased agroecological richness - Increases in agroecological richness lead to greater diet diversity - Parcel size and species richness are linked to the healthy diet index (NOVA-based) |
| García et al., 2023^31^ | - Participants with major reductions in UPF consumption reduced CO2eq and energy impact - Water use increased as the percentage of UPF was reduced - Participants in T1 (max UPF% reduction) had the highest reductions of GHGs and energy consumption - Higher UPF consumption leads to increased GHGs and energy use, and decreased water use - Lower water use and higher energy consumption and CO2 emissions are expected with increased UPF consumption |
| Garzillo et al., 2022^32^ | - In the crude models, the dietary contribution of ultra-processed foods was linearly associated with the carbon and water footprints of the Brazilian diet. After adjustment for potential confounders, the association remained significant only regarding the diet water footprint, which increased by 10.1% between the lowest and highest quintile of the contribution of ultra-processed foods. Additional adjustment for total energy intake eliminated this association indicating that the dietary contribution of ultra-processed foods increases the diet water footprint by increasing energy intake |
| Grech et al., 2022^33^ | - DF intake leads to higher non-protein energy intake compared to UPF, thus DF classification is better at distinguishing foods associated with high energy intakes in Australia - Agreement between discretionary food %E and UPF %E tertiles is moderate - 38.4% of foods were classified in adjacent tertile of discretionary food %E and UPF %E - 10.4% of foods were classified in opposite tertile of discretionary food %E and UPF %E |
| S34 Gupta et al., 2019^34^ | - Both energy density and NRF values were statistically significant among both food groups and food processing categories. - Ultra-processed foods have lower nutrient density and higher energy density compared to unprocessed foods - Ultra-processed foods cost $0.55/100 kcal, while unprocessed foods cost $1.45/100 kcal - Unprocessed foods have the highest NRF9.3 score and the lowest energy density - Unprocessed foods undoubtedly fall in the high NRF category as they have vitamins and minerals, have low energy density, and are unprocessed/fresh like meat, fruit, and vegetables. - However, some of the ultra-processed foods fall into the nutrient-rich category - 50% of ultra-processed foods fall into the low NRF score category - Prices for unprocessed foods were significantly above all other NOVA categories, the lowest cost was for culinary ingredients, mostly fats, oils, and sweeteners: $0.14/100 kcal. |
| S35 Gupta et al., 2021^35^ | - Higher percentage energy from UP foods linked to higher energy density and lower HEI-2015 and NRF9.3 scores - The bottom decile of diet cost is associated with 67.5% energy from UP foods, while the top decile with only 48.7% - Percentage energy from UP foods inversely linked to lower food expenditures and diet cost |
| Hallinan et al., 2021^36^ | - Ultra-processed foods were the main sources of added sugar, saturated fat, and sodium. Ultra-processed foods also contributed most vitamin E, thiamin, niacin, folate, and calcium, and were the main sources of plant protein. LP models failed to create optimal diets using unprocessed foods only and ultra-processed foods only: no mathematical solution was obtained. Relaxing the vitamin D criterion led to optimized diets based on unprocessed or ultra-processed foods only. However, food patterns created using unprocessed foods were significantly more expensive compared to those created using foods in the ultra-processed category |
| Hässig et al., 2023^37^ | - Perceived processing was lowest in NOVA1 and highest in category NOVA4 - Perceived healthiness was highest in NOVA1 and lowest in category NOVA4 - Significant correlation between NOVA system and Nutri-Score (r = 0.58, p <0.01) |
| Julia et al., 2023^38^ | - The study found that ultra-processed food consumption had a significant effect on dietary quality; the cross-effect between the nutritional quality of the foods consumed and ultra-processing was also substantial. - The difference in the proportion of ultra-processed foods consumed between the first and last quintiles of PNNS-GS2 score was significant (from 20.04% of UPFp in quintile 1 to 13.06% UPFp in quintile 5) - The overall effect from nutritional quality and ultra-processed foods consumption accounted for 70% of the total effect. |
| Juul et al., 2019^39^ | - Substituting 10 %E from minimally processed foods and processed culinary ingredients for ultra-processed foods decreased total HEI-2015 score by 1·8 points - Purchases of ultra-processed foods are linked to lower Healthy Eating Index-2015 scores - The energy share of ultra-processed foods was greater among households with a HEI-2015 total score of <40 compared with ≥60 - On average, households purchasing the least ultra-processed foods had 10·7 points higher HEI-2015 total scores than those purchasing the most ultra-processed foods - Households purchasing the highest proportion of ultra-processed foods were furthest from meeting the recommendations of the Dietary Guidelines for Americans 2015–2020 for all food groups and nutrients except whole grains, dairy, fatty acid ratio, and saturated fats. |
| Juul et al., 2021^40^ | - Over 17 years of follow-up, ultra-processed food consumption decreased from 7·5 to 6·0 servings/d and minimally processed food consumption decreased from 11·9 to 11·3 servings/d (Ptrend < 0·001). Changes in intakes of processed foods, culinary ingredients, and culinary preparations were minimal. Trends were similar by sex, BMI, and smoking status. DGAI-2010 score increased from 60·1 to 61·5, |
| Kesse-Guyot et al., 2023^41^ | - Overall, UPF represented 19% of the diet yet contributed 24% to the diet’s greenhouse gas emissions, 23% to water use, 23% to land use, and 26% to energy demand. Compared with low consumers of UPF (quintile 1; median UPF, 7%), high consumers (quintile 5; median UPF, 35%) consumed more caloric energy (+22%). Caloric intake partially explained the higher environmental pressures from high-UPF consumers. - Overall, the quality of the diet was lower in Q5 than in Q1, with a decrease in the simplified Programme National Nutrition Santé—Guidelines Score 2 (sPNNS-GS2), reflecting the adherence to food-based dietary guidelines, and the Diet Quality Index Based on the Probability of Adequate Nutrient Intake (PANDiet) reflecting the overall adequacy relative to nutrient-based references, across quintiles - Diet-related environmental pressures greatly differed depending on whether energy adjustment was applied. In the unadjusted model, most environmental indicators were higher among participants with higher %UPF, including GHGe (Q5 versus Q1, +15%), land use (+17%), fossil resource use (8%), marine and terrestrial eutrophication (+13% and 15%), particulate matter (+13%), ionizing radiation (+16%) and the overall endpoint ecological footprint (EF) score (+11%). In contrast, water use was inversely associated with %UPF, with a lower mean value found in Q5 than in Q1 (−7%). - When the adjustment for energy intake was applied, some associations were no longer significant, including GHGe. The negative association about water use was slightly strengthened (Q5 versus Q1 %UPF, −17%). In addition, associations regarding resource use, freshwater and marine eutrophication, and ozone formation were reversed, as was the association concerning the global EF score (−6%). |
| Lavigne-Robichaud et al., 2018^42^ | - Comparing the highest and lowest quintiles of scores, adjusted odds ratios for Metabolic Syndrome were 0.70 for aHEI-2010, 1.06 for FQS, and 1.90 for UPP contribution to dietary energy intake. - When comparing with the dietary share of UPP, negative correlations were observed with the aHEI-2010 score (r=−0·47) as well as with the FQS (r=−0·29). All correlations were significant. - After adjustment for confounding variables, diet quality scores showed a significant trend with the prevalence of Metabolic Syndrome. - Higher quintiles of energy contribution of UPP showed a significant association with Metabolic Syndrome. |
| Liu et al., 2022^43^ | - Higher consumption of ultra-processed foods was associated with lower AHA and HEI2015 dietary scores - Both children and adults consuming more ultra-processed foods had significantly higher odds of having poor diet quality - Poor diet quality doubled from the lowest to the highest quintile of UPF consumption |
| Maia et al., 2022^44^ | - Optimized (to Brazilian FBDG) diets gradually increased in nutritional profile contributions and unprocessed or minimally processed foods proportion while the relative contribution of processed culinary ingredients and ultra-processed foods decreased in optimized diets, besides, the average cost of optimized diets decreased compared to current diet |
| Marchese et al., 2022^45^ | - A higher percentage of energy from UPF was inversely associated with diet quality and with lower DGI scores - Energy from UPF (continuous) was associated with all diet quality components except for milk, yogurt, cheese, and/or their alternatives and limiting intake of foods and drinks containing added salt |
| Martinez-Perez & Arroyo-Izaga, 2021^46^ | - Regarding the comparison between the NPMs and processing level classiﬁcation, in general, a moderate agreement was observed between the NOVA system and each of the NPMs, separately and also combined. The lowest level of agreement between NPMs and the NOVA system was obtained for cold/hot foods. (The highest level (i.e., "moderate") was for the "total" category for both nutrient profiling score systems |
| Martinez-Perez et al., 2021^47^ | - Higher UPF consumption is linked to lower Mediterranean diet adherence - The IARC system includes the highest number of food items in the UPF group - NOVA classification had the lowest number of food items classified as UPF - Total fat intake was higher in the highest UPF consumption quintile only with NOVA classification - Agreement analysis showed low subject agreement between DP classification systems (IFIC, UNC) for all pairwise comparisons - NOVA-IARC comparison had the lowest concordance and percentage of subject agreement - Subjects with high UPF consumption have a higher intake of energy, simple sugars, saturated fat, and sodium, and lower fiber intake. |
| Martinez‑Perez et al., 2022^48^ | - -Comparison of the four classification systems (IARC, IFIC, UNC, and NOVA) and the sQ-HPF showed a fair to high agreement - Fair agreement was observed between sQ-HPF and NOVA tertiles of HPF - MedDiet adherence score was significantly higher in the lower HPF consumption population tertile compared to the highest tertile |
| Mendes et al., 2021^49^ | - Regarding the nutritional profile of processed foods and ultra-processed foods, only 3.5% of them did not exceed any of the critical nutrients and 59.4% was excessive in total fat; 50.3% in added sugars; 49.4% in saturated fat, 46.9% in sodium and 45.7% in trans-fat - About prices, processed foods had a statistically significant higher cost (R$ 3.27/100 g) than the other groups of the NOVA classification fresh or minimally processed foods: R$ 2.71/100 g; processed culinary ingredients: R$ 2.41/100 g and ultra-processed foods: R$ 2.75/100 g. |
| Mendoza-Velázquez et al., 2022^50^ | - Nutrient density is positively linked to per 100 kcal food cost. - Ultra-processed foods contain more energy, fat, sugar, and salt and have lower NRF scores compared to minimally processed foods, beside, UPF is less expensive compared to MPF - Nutrient-rich foods below the median per 100 kcal costs included MPF foods, but also processed foods (PF) and UPF. - Minimally processed foods had the highest NRF9 scores and the lowest LIM and LIMt scores. - Foods in the top quartile of NRF9.3 scores were mostly minimally processed (44.52%), while those in the bottom quartile were mostly ultra-processed (77.70%). - The top quartile of NRF6.3 scores were mostly PF (56.08%), UPF (22.30%), and MPF (21.62%). Foods in the bottom quartile of NRF6.3 scores were mostly UPF foods (77.55%), followed by PF (12.24%) and culinary ingredients (7.48%), as opposed to MPF (2.72%) - Foods in the UPF category had higher energy density and contained more saturated fat, total sugar, and added sugar, as compared to other categories. - Foods in the UPF category tended to be energy-dense, had lower nutrient density scores, and lower per kcal costs. However, as indicated in the Figure 5 panels, some UPF foods were both inexpensive and nutrient-rich. Some of those foods provided the needed priority nutrients, as captured by the NRF6.3 score |
| Mignogna et al., 2022^51^ | - Greater intake of unprocessed/minimally processed foods was associated with lower levels of E-DII, - E-DII increased with the consumption of processed culinary ingredients, processed food, and UPF |
| Morales et al., 2020^52^ | - Ultra-processed breakfast cereals have low nutritional quality and high energy density - Acrylamide content correlates with HMF, sugar content, and NOVA food classification system scores - Significant differences found in acrylamide and HMF distribution within NOVA groups - NOVA system may not accurately reflect the intensity of thermal treatment in breakfast cereals; differences in heat-induced chemical markers not significantly different between NOVA-3 and NOVA-4 groups |
| Otten et al., 2017^53^ | - No minimum wage effect was detected on supermarket food prices by USDA food group, food processing, or nutrient density categories |
| Phulkerd et al., 2023^54^ | - Products eligible for HCL had the highest proportion of UPF products (72.7%) - Poor agreement between NOVA and other systems in classifying products as healthy/healthier - Group A products had 69.2% UPF products, while 'marketing permitted' products had 65.0% - 30.8% of non-UPF products were classified under HCL as healthy/healthier - DOH, WHO SEA, and HCL systems showed varying percentages for classifying products as healthy/healthier |
| Phulkerd et al., 2023^55^ | - Growth in consumer expenditure per capita on UPFs is forecast to grow between 26% and 30% till 2026 - More than half of UPFs exceeded at least one nutrient cutoff, 59.3% for total fats, 24.8% for saturated fats, 68.2% for total sugars, and 94.3% for sodium. |
| Pulker et al., 2018^56^ | - The food groups recommended in the NOVA classification system as the foundation of healthy dietary patterns, unprocessed and minimally processed foods, achieved a mean HSR of 4.4. Processed foods achieved a mean HSR of 3.5, and processed culinary ingredients achieved a mean HSR of 2.6. The food group recommended to be avoided in the NOVA classification system, nutrient-poor UPF, achieved a mean HSR of 2.5. |
| Rizzolo-Brime et al., 2023^57^ | - Of the 148 products, the majority were low in sugars but moderate in carbohydrates, total and saturated fat, and high in salt. The main vegetable protein sources were soy (91/148) and wheat gluten (42/148). Comparatively, 43/148 contained animal protein, the most common being egg. Overall, PBMAs had a long list of ingredients and additives, and they were classified as ultra-processed foods (UPFs) according to the NOVA system. - According to the NOVA classification system, 93.9% of the products were categorized as ultra-processed food (Group 4) and the remaining 6.08% of PBMAs were categorized as processed food (Group 3, for the plant-based mince group). |
| Robert et al., 2022^58^ | - More resilient participants had greater overall diet quality, greater intakes of seafood, whole-grain foods, fats, unsalted oleaginous fruits, and alcoholic beverages, and lower intakes of UPFs, starchy foods, dairy desserts, sugary fatty products, and sugar and confectionery (all P < 0.05) |
| Rodrigues et al., 2016^59^ | - The NOVA model was stricter compared with the UK/Ofcom model, classifying more products as 'less healthy' (91.40%) compared with the NP-based model (74.95%), also, it was stricter compared with the UK/Ofcom model when applied to food products with nutrient claims - Agreement between food classification systems models for foods bearing nutrient claims was 74.44%, whereas the agreement for foods without nutrient claims was 84.23% - UK/Ofcom model does not consider non-caloric sweeteners and artificial food additives, unlike the NOVA model - Foods like sliced bread, chicken nuggets, and diet carbonated drinks are classified as 'healthier' by the UK/Ofcom model but not by the NOVA model |
| Romero Ferreiro et al., 2021^60^ | - Ultra-processed foods are present in all Nutri-Score categories - 75.50% of NOVA 4 foods are classified as medium-low nutritional quality by Nutri-Score - Nutri-Score as a continuous score is lower (healthier) for unprocessed or minimally processed foods than for ultra-processed foods (it is higher; unhealthier) - The percentage of ultra-processed foods increases with lower nutritional categories |
| Rossato et al., 2023^61^ | - The multivariable analysis shows a rise in AHEI-2010, aMED, and DASH-diet scores from lowest to highest UMP quintiles. - The AHEI-2010, aMED, and DASH-diet scores are consistently higher in the fifth quintile of UMP intake. - The first quintile of percent of energy from UPF had lower diet-quality mean scores - In HPFS, AHEI-2010, aMED, and DASH diet scores were lower in the highest quintiles of UPF intake |
| Ruggiero et al., 2021^62^ | - For all ages, Mediterranean diet was inversely associated with UPF - Compared with the lowest (Q1), adult subjects in the highest quartile of UPF consumption (Q4) had a higher intake of energy, sugar, protein, total fat, saturated fat, polyunsaturated fats, dietary cholesterol, and Na, but lower intakes of total carbohydrate, fiber and monounsaturated fat - Good adherence to the Mediterranean diet is associated with 5.08% less energy from ultra-processed foods and 5.98% higher energy from unprocessed/minimally processed food |
| Salomé et al., 2021^63^ | - -MPFp* was positively associated with animal protein intake and plant protein diversity - PFp* was positively associated with plant protein intake and negatively with plant protein diversity - The PANDiet score and its sub-scores increased with the proportion of MPF and to a lesser extent with the proportion of PF, similarly, the PDI and hPDI scores increased with the proportion of MPF and decreased with the proportion of UPF, while uPDI was in a negative association with MPFp (p: proportion in diet) |
| Shim et al., 2020^64^ | - KHEI score inversely related to daily energy from ultra-processed foods, however, all levels of ultra-processed food consumption linked to poor diet quality - -.UPF intake was found to be in positive associations with energy, sugar, fat, and sodium - UPF intake was found to have negative associations with carbohydrates, fiber, and various nutrients - Most adequacy and moderation components are negatively associated with high energy from ultra-processed foods, except for milk and dairy group - Both sodium and potassium were negatively associated with percentage of energy from ultra-processed foods. However, the sodium-to-potassium ratio was high regardless of quintile of energy contribution from ultra-processed foods, and the ratio was positively associated with percentage of total energy from ultra-processed foods |
| Siqueira et al., 2021^65^ | - Ultra-processed foods have higher energy density and lower NRF8.2 scores than unprocessed foods - Processed foods also have higher energy density compared to unprocessed foods - Unprocessed foods cost more per kJ compared to processed and 'ultra-processed' foods - Unprocessed foods have a better nutrient-to-cost ratio than processed and 'ultra-processed' foods - Nutrient affordability metrics confirm unprocessed foods have a better nutrient-to-cost ratio than processed and 'ultra-processed' foods |
| Sneed et al., 2023^66^ | - There was a significant correlation between higher rates of UPF (NOVA 4) consumption and unhealthier diet quality (e.g., lower HEI total and subcomponent scores) |
| Spiteri et al., 2018^67^ | - The majority of new products were classified in each scheme's least healthy category (i.e. red, discretionary, and ultra-processed). Fruits and vegetables represented just 3% of new products. Healthier Australia Commitment (HAC) members launched a significantly greater proportion of foods classified as red (59% vs 51% for members and non-members, respectively) discretionary (79% vs 61%), and ultra-processed (94% vs 81%), and significantly fewer were classified as green (8% vs 15%), core foods (18% vs 36%) and minimally processed (0% vs 6%) (all p < 0.001). |
| Trübwasser et al., 2022^68^ | - 436 food outlets and 246 food or drink advertisements around each school on average: 89.9% of the advertisements were of ultra-processed foods, mainly sugar-sweetened beverages (SSBs), and 26.3% of the outlets displayed SSBs or sweets, while 17.9% displayed fresh fruits and vegetables - Adolescents had poor dietary diversity, consuming only 3.6 food groups out of 10 in the last 24 hours - Higher assets in adolescents’ households were associated with better dietary diversity and consumption of healthy food groups |
| Valenzuela et al., 2022^69^ | - For the NOVA system, most of the foods were not recommended, while the other two systems placed most foods in their recommended classification. - Nutri-Score and NOVA classification showed similarities and inconsistencies. - 88.1% compatibility was found in categorizing the least recommended products by each system (E and NOVA 4). - There was total congruence between NOVA categories and the absence of warning labels for certain products. - Nutri-Score and FoP warning labels had similar classification guidelines, differing from the dimension targeted by NOVA. |
| van Dam & Vandevijvere, 2022^70^ | - The median proportion of foods with Nutri-Score A or B within product portfolios was 38%, while the median proportion of non-permitted products was 84% and the median proportion of ultra-processed food products was 63%. - Stronger company commitments did not translate into better performance metrics. |
| van Dam, Reimes & Vandevijvere, 2022^71^ | - Food companies' portfolios consist of 0-100% A and B Nutri-Score products (median=29%) - The portfolios' median proportion of products not permitted for children: 81% (range=12%-100%) - The portfolios' median proportion of ultra-processed foods: 75% (range=2%-100%) - No significant correlations between commitments and performance indicators were found |
| Vandevijvere et al., 2020^72^ | - The average price per 100 kcal for UPF was significantly cheaper than for MPF. UPF contributed between 21.9% (female adults) and 29.9% (young boys), while MPF contributed between 29.5% (male adolescents) and 42.3% (female adults) to the daily dietary cost. |
| Vellinga et al., 2022^73^ | - UPFD consumption determined 45% of GHG emissions, 23% of blue water use, and 39% of expenses for daily food consumption. - Ultra-processed foods (UPF) are more energy-dense and less healthy than unprocessed or minimally processed foods (MPF) they also are associated with higher greenhouse gas (GHG) emissions and lower blue water use and are cheaper - The energy and sugar content of UPD were similar to those of unprocessed or minimally processed drinks (MPD); associated with similar GHG emissions but blue water use was less, and they were also more expensive. - Compared with unprocessed or minimally processed foods and drinks, UPF and UPD were found to be less healthy considering their high energy, SFA, sugar and sodium content. - The consumption of UPFD was found to be unhealthy given its significant contribution to the intake of nutrients such as sodium (72%), sugar (64%) and SFA (54%). |
| Vellinga et al., 2023^74^ | - High UPF consumption was statistically significantly inversely associated with all environmental impact indicators, whereas high UPD consumption was, except for land use, statistically significant positively associated with all environmental impact indicators - Higher UPFD consumption (Q4vsQ1) was significantly associated with slightly higher eutrophication of fresh water and GHG emissions compared to low UPFD consumption. - On the contrary, blue water consumption, eutrophication of marine water, land use, and terrestrial acidification were lower for those with higher UPFD consumption compared with those with lower UPFD consumption (Q4vsQ1) |
| Vergeer et al., 2019^75^ | - Overall, the most-processed products under both systems were more likely to be lower in protein, and higher in total and free sugars, compared with less-processed foods, the direction and strength of the association between other nutrients/components and level of processing were less consistent - Sodium: under NOVA, overall median sodium contents of ultra-processed products were higher than those of unprocessed/minimally processed foods and processed culinary ingredients, but lower than those of processed foods. - Saturated Fat: based on NOVA, median levels of saturated fat in ultra-processed products were lower than those of processed culinary ingredients among the overall sample, but higher than those of unprocessed/minimally processed foods and processed foods. - Total and Free Sugars: according to NOVA, the overall median total and free sugars per 100 g (or 100 mL) in ultra-processed foods were greater than those of unprocessed/minimally processed foods, processed foods, and culinary ingredients. - Under NOVA, mean and median fiber contents of ultra-processed products among the total sample were comparable to those of foods in other processing categories for the total sample. Compared with ultra-processed products, unprocessed/minimally processed foods were more likely to be higher in fiber, while processed foods were more likely to be lower in fiber when adjusted for food category. - Protein: according to NOVA, average protein contents of ultra-processed foods per 100 g (or 100 mL) in the total sample were higher than those of processed culinary ingredients but less than those of unprocessed minimally processed and processed foods |
| Vicente et al., 2023^76^ | - MDS and DII showed similar trends in indicating inflammatory potential - UPR had a non-significant, inverse correlation with the other indicators - Only MDS had a significant positive explanation for AII values - The combination of MDS or DII with UPR did not change the association level with the anti-inflammatory index |
| Vogliano et al., 2021^77^ | - Ultra-processed foods (NOVA 4) were consumed exponentially according to proximity to the urban center, and the most commonly consumed such foods were white bread, instant noodles, donuts, Milo drink mix, milk tea, and sausages. - Urban populations consumed significantly more protein and ultra-processed foods (NOVA 4), were more likely to eat takeout foods, and had less diverse diets compared to rural populations. Less than half of urban participants met their recommended NSFV intakes. - Food fortification may solve single micronutrient deficiencies but could ultimately reduce the sustainability of the indigenous food system and give rise to diet-related noncommunicable diseases (NCDs), particularly since most fortified foods are imported and tend to be processed. |

**orange: studies that met with all inclusion criteria, however, the association between NOVA and other sustainable dietary quality indicator was not directly analyzed*

References

S1 Abreu S, Liz Martins M. Cross-Classification Analysis of Food Products Based on Nutritional Quality and Degree of Processing. Nutrients. 2023;15(14):3117. doi:10.3390/nu15143117.

S2 Aceves-Martins M, Bates RL, Craig LC, et al. Nutritional quality, environmental impact and cost of ultra-processed foods: a UK food-based analysis. Int. J. Environ. Res. Public Health. 2022;19(6):3191. doi:10.3390/ijerph19063191

S3 Angelino D, Dinu M, Gandossi B, et al. Processing and nutritional quality of breakfast cereals sold in Italy: results from the Food Labelling of Italian Products (FLIP) Study. Nutrients. 2023;15(8):2013. doi:10.3390/nu15082013

S4 Baldridge AS, Huffman MD, Taylor F, et al. The healthfulness of the US packaged food and beverage supply: a cross-sectional study. Nutrients. 2019;11(8):1704. doi:10.3390/nu11081704

S5 Barrett EM, Gaines A, Coyle DH, et al. Comparing product healthiness according to the Health Star Rating and the NOVA classification system and implications for food labelling systems: An analysis of 25 486 products in Australia. Nutr Bull. 2023;48(4):523-534. doi:10.1111/nbu.12640. Epub 2023 Oct 28

S6 Batal M, Johnson-Down L, Moubarac JC, et al. Quantifying associations of the dietary share of ultra-processed foods with overall diet quality in First Nations peoples in the Canadian provinces of British Columbia, Alberta, Manitoba and Ontario. Public Health Nutr. 2018;21(1):103-113. doi:10.1017/S1368980017001677

S7 Batista CHK, Leite FHM, Borges CA. Association between advertising patterns and ultra-processed food in small markets. Ciência & Saúde Coletiva, 2022;27:2667-2678. doi:10.1590/1413-81232022277.19122021.

S8 Baye K, Yaregal Z. The Global Diet Quality Score predicts diet quality of women of reproductive age in Addis Ababa, Ethiopia. BJN. 2023;130:1573-1579. doi:10.1017/S0007114523000508.

S9 Berardy A, Fresán U, Matos RA, et al. Environmental impacts of foods in the Adventist health study-2 dietary questionnaire. Sustainability. doi:2020;12:10267. doi:10.3390/su122410267

S10 Blanchet R, Willows N, Johnson S, et al. Traditional food, health, and diet quality in Syilx Okanagan adults in British Columbia, Canada. Nutrients. 2020;12:927. doi:10.3390/nu12040927

S11 Bleiweiss-Sande R, Chui K, Evans EW, et al. Robustness of Food Processing Classification Systems. Nutrients. 2019;11:1344. doi:10.3390/nu11061344.

S12 Bonaccio M, Di Castelnuovo A, Ruggiero E, et al. Joint association of food nutritional profile by Nutri-Score front-of-pack label and ultra-processed food intake with mortality: Moli-sani prospective cohort study. BMJ. 2022,378. doi:10.1136/bmj-2022-070688

S13 Braesco V, Souchon I, Sauvant P, et al. Ultra-processed foods: how functional is the NOVA system?. Eur. J. Clin. Nutr. 2022;76(9):1245-1253. doi:10.1038/s41430-022-01099-1

S14 Cediel G, Reyes M, Corvalán C, et al. Ultra-processed foods drive to unhealthy diets: evidence from Chile. Public Health Nutr. 2021;24(7):1698-1707. doi:10.1017/S1368980019004737

S15 Chen YC, Huang YC, Lo YTC, et al. Secular trend towards ultra-processed food consumption and expenditure compromises dietary quality among Taiwanese adolescents. Food Nutr Res. 2018,62. doi:10.29219/fnr.v62.1565

S16 Cooper SL, Pelly FE, Lowe JB. Assessment of the construct validity of the Australian Health Star Rating: a nutrient profiling diagnostic accuracy study. Eur. J. Clin. Nutr. 2017;71(11):1353-1359. doi:10.1038/ejcn.2017.23

S17 da Rocha BRS, Rico-Campà A, Romanos-Nanclares A, et al. Adherence to Mediterranean diet is inversely associated with the consumption of ultra-processed foods among Spanish children: The SENDO project. Public Health Nutr. 2021;24:3294-3303. doi:10.1017/S1368980020001524

S18 da Silva JT, Garzillo JMF, Rauber F, et al. Greenhouse gas emissions, water footprint, and ecological footprint of food purchases according to their degree of processing in Brazilian metropolitan areas: a time-series study from 1987 to 2018. Lancet Planetary Health. 2021,5:775-785.

S19 Davidou S, Christodoulou A, Fardet A, Frank K. The holistico-reductionist Siga classification according to the degree of food processing: an evaluation of ultra-processed foods in French supermarkets. Food Funct. 2020;11(3):2026-2039. doi:10.1039/C9FO02271F

S20 de Las Heras-Delgado S, Shyam S, Cunillera È, et al Are plant-based alternatives healthier? A two-dimensional evaluation from nutritional and processing standpoints. Food Res Int. 2023;169:112857. doi:10.1016/j.foodres.2023.112857

S21 de Moraes MM, Oliveira B, Afonso C, et al. An ultra-processed food dietary pattern is associated with lower diet quality in Portuguese adults and the elderly: The UPPER project. Nutrients. 2021;13:4119. doi:10.3390/nu13114119.

S22 Delgado-Rodríguez R, Moreno-Padilla M, Moreno-Domínguez S, Cepeda-Benito A. Food addiction correlates with emotional and craving reactivity to industrially prepared (ultra-processed) and home-cooked (processed) foods but not unprocessed or minimally processed foods. Food Qual Prefer. 2023;110:104961. doi:10.1016/j.foodqual.2023.104961

S23 Derbyshire, E. Are all ‘ultra-processed’foods nutritional demons? A commentary and nutritional profiling analysis. Trends Food Sci Technol. 2019;94:98-104. doi:10.1016/j.tifs.2019.08.023

S24 Detopoulou P, Dedes V, Pylarinou I, et al. Dietary acid load is associated with waist circumference in university students with low adherence to the Mediterranean diet: The potential role of ultra-processed foods. Clin Nutr ESPEN. 2023;56:43-51. doi:10.1016/j.clnesp.2023.05.005

S25 Dickie S, Woods J, Machado P, Lawrence M. Nutrition classification schemes for informing nutrition policy in Australia: nutrient-based, food-based, or dietary-based?. Curr Dev Nutr. 2022;6(8):112. doi:10.1093/cdn/nzac112

S26 Dinu M, Tristan Asensi M, Pagliai G, et al. Consumption of ultra-processed foods is inversely associated with adherence to the Mediterranean diet: a cross-sectional study. Nutrients. 2022;14:2073. doi:10.3390/nu14102073

S27 Estell ML, Barrett EM, Kissock KR, et al. Fortification of grain foods and NOVA: the potential for altered nutrient intakes while avoiding ultra-processed foods. Eur J Nutr. 2022;1-11. doi:10.1007/s00394-021-02701-1

S28 Fardet A, Rock E. How to protect both health and food system sustainability? A holistic ‘global health’-based approach via the 3V rule proposal. Public Health Nutr. 2020;23:3028-3044. doi:10.1017/S136898002000227X

S29 Fardet A, Méjean C, Labouré H, et al. The degree of processing of foods which are most widely consumed by the French elderly population is associated with satiety and glycemic potentials and nutrient profiles. Food Funct. 2017;8:651-658. doi:10.1039/c6fo01495j

S30 Gallegos-Riofrío CA, Waters WF, Carrasco A, et al. Caliata: an Indigenous Community in Ecuador offers lessons on food sovereignty and sustainable diets. Curr Dev Nutr. 2021;5:61-73. doi:10.1093/cdn/nzab009

S31 García S, Pastor R, Monserrat-Mesquida M, et al. Ultra-processed foods consumption as a promoting factor of greenhouse gas emissions, water, energy, and land use: A longitudinal assessment. Sci Total Environ. 2023;891:164417. doi:10.1016/j.scitotenv.2023.164417

S32 Garzillo JMF, Poli VFS, Leite FHM, et al. Ultra-processed food intake and diet carbon and water footprints: a national study in Brazil. Revista de saude publica, 2022;56:6. doi:10.11606/s1518-8787.2022056004551

S33 Grech A, Rangan A, Allman-Farinelli M, et al. A Comparison of the Australian Dietary Guidelines to the NOVA Classification System in Classifying Foods to Predict Energy Intakes and Body Mass Index. Nutrients. 2022;14:3942. doi:10.3390/nu14193942

S34 Gupta S, Hawk T, Aggarwal A, Drewnowski A. Characterizing ultra-processed foods by energy density, nutrient density, and cost. Front Nutr. 2019;6:454858. doi:10.3389/fnut.2019.00070

S35 Gupta S, Rose CM, Buszkiewicz J, et al. Characterising percentage energy from ultra-processed foods by participant demographics, diet quality and diet cost: Findings from the Seattle Obesity Study (SOS) III. BJN. 2021;126:773-781. doi:10.1017/S0007114520004705

S36 Hallinan S, Rose C, Buszkiewicz J, Drewnowski A. Some ultra-processed foods are needed for nutrient adequate diets: linear programming analyses of the Seattle obesity study. Nutrients. 2021;13:3838. doi:10.3390/nu13113838

S37 Hässig A, Hartmann C, Sanchez-Siles L, Siegrist M. Perceived degree of food processing as a cue for perceived healthiness: the NOVA system mirrors consumers’ perceptions. Food Qual Prefer. 2023;110:104944. doi:10.1016/j.foodqual.2023.104944

S38 Julia C, Baudry J, Fialon M, et al. Respective contribution of ultra-processing and nutritional quality of foods to the overall diet quality: results from the NutriNet-Santé study. Eur J Nutr. 2023;62:157-164. doi:10.1007/s00394-022-02970-4

S39 Juul F, dos Santos Simões B, Litvak J, et al. Processing level and diet quality of the US grocery cart: is there an association?. Public Health Nutr. 2019;22:2357-2366. doi:10.1017/S1368980019001344

S40 Juul F, Lin Y, Deierlein AL, et al. Trends in food consumption by degree of process. BJN. 2021;126:1861-1871. doi:10.1017/S000711452100060X

S41 Kesse-Guyot E, Allès B, Brunin J, et al. Environmental impacts along the value chain from the consumption of ultra-processed foods. Nature Sustainability. 2023;6(2):192-202. doi:10.1038/s41893-022-01013-4

S42 Lavigne-Robichaud M, Moubarac JC, Lantagne-Lopez S, et al. Diet quality indices in relation to metabolic syndrome in an Indigenous Cree (Eeyouch) population in northern Québec, Canada. Public Health Nutr. 2018;21(1):172-180. doi:10.1017/S136898001700115X

S43 Liu J, Steele EM, Li Y, et al. Consumption of ultraprocessed foods and diet quality among US children and adults. Am J Prev Med. 2022;62(2):252-264. doi:10.1016/j.amepre.2021.08.014

S44 Maia EG, Passos CMD, Granado FS, et al. Replacing ultra-processed foods with fresh foods to meet the dietary recomendations: a matter of cost?. Cadernos de Saúde Pública. 2022;37:e00107220. doi:10.1590/0102-311X00107220

S45 Marchese L, Livingstone KM, Woods JL, et al. Ultra-processed food consumption, socio-demographics and diet quality in Australian adults. Public Health Nutr. 2022;25(1):94-104. doi:10.1017/S1368980021003967

S46 Martinez-Perez N, Arroyo-Izaga M. Availability, nutritional profile and processing level of food products sold in vending machines in a Spanish public university. Int. J. Environ. Res. Public Health. 2021;18(13):6842. doi:10.3390/ijerph18136842

S47Martinez-Perez C, San-Cristobal R, Guallar-Castillon P, et al. Use of different food classification systems to assess the association between ultra-processed food consumption and cardiometabolic health in an elderly population with metabolic syndrome (PREDIMED-Plus Cohort). Nutrients. 2021;13(7):2471. doi:10.3390/nu13072471.

S48 Martinez-Perez C, Daimiel L, Climent-Mainar C, et al. Integrative development of a short screening questionnaire of highly processed food consumption (sQ-HPF). Int J Behav Nutr Phys Act. 2022;19(1):6. doi:10.1186/s12966-021-01240-6

S49 Mendes C, Miranda L, Claro R, Horta P. Food marketing in supermarket circulars in Brazil: An obstacle to healthy eating. Preventive Med Reports. 2021;21:101304. doi:10.1016/j.pmedr.2020.101304

S50 Mendoza-Velázquez A, Lara-Arévalo J, Siqueira KB, et al. Affordable nutrient density in brazil: nutrient profiling in relation to food cost and NOVA category assignments. Nutrients. 2022;14(20):4256. doi:10.3390/nu14204256

S51 Mignogna C, Costanzo S, Di Castelnuovo A, et al. The inflammatory potential of the diet as a link between food processing and low-grade inflammation: An analysis on 21,315 participants to the Moli-sani study. Clin Nutr. 2022;41:2226-2234. doi:10.1016/j.clnu.2022.08.020

S52 Morales FJ, Mesías M, Delgado-Andrade C. Association between heat-induced chemical markers and ultra-processed foods: A case study on breakfast cereals. Nutrients. 2020;12:1418. doi:10.3390/nu12051418

S53 Otten JJ, Buszkiewicz J, Tang W, et al. The impact of a city-level minimum-wage policy on supermarket food prices in Seattle-King County. Int. J. Environ. Res. Public Health. 2017;14:1039. doi:10.3390/ijerph14091039

S54 Phulkerd S, Dickie S, Thongcharoenchupong N, et al. Choosing an effective food classification system for promoting healthy diets in Thailand: a comparative evaluation of three nutrient profiling-based food classification systems (government, WHO, and Healthier Choice Logo) and a food-processing-based food classification system (NOVA). Front Nutr. 2023;10:1149813. doi:10.3389/fnut.2023.1149813

S55 Phulkerd S, Thongcharoenchupong N, Dickie S, et al. Profiling ultra-processed foods in Thailand: sales trend, consumer expenditure and nutritional quality. Global Health. 2023;19:64. doi:10.1186/s12992-023-00966-1

S56 Pulker CE, Trapp GS, Scott JA, Pollard CM. Alignment of supermarket own brand foods’ front-of-pack nutrition labelling with measures of nutritional quality: An Australian perspective. Nutrients. 2018;10:1465. doi:10.3390/nu10101465

S57 Rizzolo-Brime L, Orta-Ramirez A, Puyol Martin Y, Jakszyn P. Nutritional assessment of plant-based meat alternatives: a comparison of nutritional information of plant-based meat alternatives in Spanish supermarkets. Nutrients. 2023;15:1325. doi:10.3390/nu15061325

S58 Robert M, Shankland R, Bellicha A, et al. Associations between resilience and food intake are mediated by emotional eating in the NutriNet-Santé Study. Journal of Nutr. 2022;152:1907-1915. doi:10.1093/jn/nxac124

S59 Rodrigues VM, Rayner M, Fernandes AC, et al. Nutritional quality of packaged foods targeted at children in Brazil: which ones should be eligible to bear nutrient claims?. Int J Obes. 2017;41:71-75. doi:10.1038/ijo.2016.167

S60 Romero Ferreiro C, Lora Pablos D, Gómez de la Cámara A. Two dimensions of nutritional value: Nutri-Score and NOVA. Nutrients. 2021;13: 2783. doi:10.3390/nu13082783

S61 Rossato SL, Khandpur N, Lo CH, et al. Intakes of unprocessed and minimally processed and Ultraprocessed food are associated with diet quality in female and male health professionals in the United States: A prospective analysis. J Acad Nutr Diet. 2023;123:1140-1151. doi:10.1016/j.jand.2023.03.011

S62 Ruggiero E, Esposito S, Costanzo S, et al. Ultra-processed food consumption and its correlates among Italian children, adolescents and adults from the Italian Nutrition & Health Survey (INHES) cohort study. Public Health Nutr. 2021;24:6258-6271. doi:10.1017/S1368980021002767

S63 Salomé M, Arrazat L, Wang J, et al. Contrary to ultra-processed foods, the consumption of unprocessed or minimally processed foods is associated with favorable patterns of protein intake, diet quality and lower cardiometabolic risk in French adults (INCA3). Eur J Nutr. 2021;60:4055-4067. doi:10.1007/s00394-021-02576-2

S64 Shim JS, Shim SY, Cha HJ, et al. Association between ultra-processed food consumption and dietary intake and diet quality in Korean adults. JAcad Nutr Diet. 2022;122:583-594. doi:10.1016/j.jand.2021.07.012

S65 Siqueira KB, Borges CA, Binoti ML, et al. Nutrient density and affordability of foods in Brazil by food group and degree of processing. Public Health Nutr. 2021;24(14):4564-4571. doi:10.1017/S1368980020004358

S66 Sneed NM, Ukwuani S, Sommer EC, et al. Reliability and validity of assigning ultraprocessed food categories to 24-h dietary recall data. Am J Clin Nutr. 2023;117:182-190. doi:10.1016/j.ajcnut.2022.10.016.

S67 Spiteri SA, Olstad DL, Woods JL. Nutritional quality of new food products released into the Australian retail food market in 2015–is the food industry part of the solution? BMC Public Health, 2018;18:1-10. doi:10.1186/s12889-018-5127-0

S68 Trübswasser U, Talsma EF, Ekubay S, et al. Factors influencing adolescents' dietary behaviors in the school and home environment in Addis Ababa, Ethiopia. Front Public Health. 2022;10:861463. doi:10.3389/fpubh.2022.861463

S69 Valenzuela A, Zambrano L, Velásquez R, et al. Discrepancy between food classification systems: Evaluation of Nutri-Score, NOVA classification and chilean front-of-package food warning labels. Int. J. Environ. Res. Public Health. 2022,19:14631. doi:10.3390/ijerph192214631

S70 Van Dam I, Vandevijvere S. Benchmarking the nutrition-related commitments and practices of major French food companies. BMC Public Health, 2022;22:1435. doi:10.1186/s12889-022-13780-y

S71 Van Dam I, Reimes N, Vandevijvere S. Benchmarking the nutrition-related commitments and practices of major Belgian food companies. Int J Behav Nutr Phys Act. 2022;19:43. doi:10.1186/s12966-022-01269-1

S72 Vandevijvere S, Pedroni C, De Ridder K, Castetbon, K. The cost of diets according to their caloric share of ultraprocessed and minimally processed foods in Belgium. Nutrients. 2020;12:2787. doi:10.3390/nu12092787

S73 Vellinga RE, van Bakel M, Biesbroek S, et al. Evaluation of foods, drinks and diets in the Netherlands according to the degree of processing for nutritional quality, environmental impact and food costs. BMC Public Health. 2022;22: 877. doi:10.1186/s12889-022-13282-x

S74 Vellinga RE, van den Boomgaard I, Boer JM, Different Levels of Ultraprocessed Food and Beverage Consumption and Associations with Environmental Sustainability and All-cause Mortality in EPIC-NL. Am J Clin Nutr. 2023;118(1):103-113. doi:10.1016/j.ajcnut.2023.05.02Siqueira KB, Borges CA, Binoti ML, et al. Nutrient density and affordability of foods in Brazil by food group and degree of processing. Public Health Nutr. 2021;24(14):4564-4571. doi:10.1017/S1368980020004358

S75 Vergeer L, Veira P, Bernstein JT, et al. The calorie and nutrient density of more-versus less-processed packaged food and beverage products in the Canadian food supply. Nutrients. 2019;11:2782. doi:10.3390/nu11112782

S76 Vicente B M, Almeida Bastos A, de Melo CM, et al. Correlation Between Different Dietary Indexes, and Their Association with An Anti-inflammatory Biomarker in Older Adults: An Exploratory Study. Eur J Geriatr Geront. 2023;5(3). doi:10.4274/ejgg.galenos.2023.2022-10-5

S77 Vogliano C, Raneri JE, Maelaua J, et al. Assessing diet quality of indigenous food systems in three geographically distinct solomon islands sites (Melanesia, Pacific Islands). Nutrients. 2020;13:3
