# Supplementary material for: Fifteen Years of NOVA Food-Processing Classification: “Friend or Foe” Among Sustainable Diet Indicators? A Scoping Review
Source: Nutr Rev. 2025 Jan 23;83(4):771–91. doi: 10.1093/nutrit/nuae207 (PMC11894255; doi:10.1093/nutrit/nuae207)
Supplement: nuae207_Supplementary_Data [file nuae207_supplementary_data.zip › nuae207_Supplementary_Data/Table_S3_Limitations_of_NOVA_mentioned_by_authors másolata.docx]

| Reference | Limitation of using NOVA in the reviewed study mentioned by authors | Identified topics |
| --- | --- | --- |
| Abreu & Martins 2023^1^ | - The ability to discriminate food processing degree with food labelling information may lead to overestimation or underestimation of foods into different NOVA groups. Nonetheless, in addition to each NOVA group’s deﬁnition, examples of foods were identiﬁed. - In addition, we cannot ignore the bias associated with selecting food products since most of the products chosen were processed and ultra-processed food products available in the Portuguese market. - All queries were discussed and resolved in consensus among all authors. | - Lack of information for NOVA classification: food labelling information may lead to overestimation or underestimation of foods into different NOVA groups - Inherent classification problems: In addition, we cannot ignore the bias associated with selecting food products since most of the products chosen were processed and ultra-processed food products available in the Portuguese market |
| Aceves-Martins et al., 2022^2^ | - Because of the lack of information on the recipes of certain items and the debate surrounding the classiﬁcation of homemade dishes versus industrially prepared ready meals, homemade products were classiﬁed as NOVA 3 foods, while ready meals were classiﬁed as NOVA 4 foods. - One of the main limitations of the NOVA system is that the basis of this classiﬁcation system is not explained further than “degree of processing” and is limited to the methods reported by the industry. As a result, some food products might have the exact same attributes, whether culinary home preparations or manufactured. Additionally, there is no clear distinction between reﬁned and whole foods within the NOVA category, nor does it include processing steps across the entire food chain, such as storage or transportation. - Further, it does not consider the amount of ingredients, such as fat, sugar, or salt, which when consumed in higher amounts can cause adverse health outcomes. Therefore, future research would beneﬁt from a food-level analysis that considers food composition beyond the broad categories of food processing. | - Lack of information for NOVA classification: Because of the lack of information on the recipes of certain items and the debate surrounding the classiﬁcation of homemade dishes versus industrially prepared ready meals, homemade products were classiﬁed as NOVA 3 foods, while ready meals were classiﬁed as NOVA 4 foods. - Inherent classification problems: classiﬁcation system is not explained further than “degree of processing” and is limited to the methods reported by the industry. As a result, some food products might have the exact same attributes, whether culinary home preparations or manufactured. Additionally, there is no clear distinction between reﬁned and whole foods within the NOVA category, nor does it include processing steps across the entire food chain, such as storage or transportation. - "Blindness" for foods high in fat, salt and/or sugar: it does not consider the amount of ingredients, such as fat, sugar, or salt , which when consumed in higher amounts can cause adverse health outcomes |
| Barrett et al., 2023^3^ | - Although our method used to identify NOVA group 4 products was systematic and based on the recommended approach in existing liter-ature (Monteiro, Cannon, Levy, et al., 2019), there is some debate about whether an ingredient-based approach is appropriate for a food-based classification system. As an example, if NOVA classifications were applied based on the broad food category rather than the presence of markers of ultra-processing in the in-gredients list, some of the discordant products found here (e.g. ‘raw’ chocolates and potato chips made from potato, salt and oil) would be categorised as ultra-pro-cessed regardless of their ingredients. However, in the absence of a definitive list of rules or conditions pub-lished to apply NOVA classifications across individual products, we consider our approach the most appropriate and justified when applied to very large datasets | - Inherent classification problems: there is some debate about whether an ingredient-based approach is appropriate for a food-based classification system |
| Batal et al., 2018^4^ | - As the NOVA food classiﬁcation was applied a posteriori from the entry and coding of the recalls, it is possible that some foods were misclassiﬁed due to limited food choices in the CNF and a lack of brand names in the database. If data entry had been designed to correspond with the NOVA classiﬁcation, more accurate distribution may have been obtained and other studies have noted this possible limitation. Since the NOVA food classiﬁcation does not group foods according to their nutritional quality, but rather based on the degree and nature of processing, some groupings might appear unusual from a nutritional perspective. However, the current study and others have shown the linear relationship between the increased contribution to energy from UPF and reduced diet quality, regardless of the inclusion/exclusion of speciﬁc food items in the UPF group and their individual nutrient proﬁle. In addition, CANDAT, our nutrient analysis software, does not allow us to disaggregate mixed foods based on recipes, so it is possible that some UPF used in culinary preparations, such as margarine used in making a traditional First Nations’ quick bread called bannock, may have been included in the fresh and minimally processed foods. Future studies could beneﬁt from standard guidelines for assignment to NOVA categories, such as brand names, home prepared v. ready-to-consume, and name of restaurant where food is purchased. Future analyses might identify the MF that, when consumed in combination with TF, will contribute to a diet for First Nations peoples that is more congruent with the current dietary recommendations and more protective against the development of NCD. | - Problems with dietary data recording: NOVA food classiﬁcation was applied a posteriori from the entry and coding of the recalls, it is possible that some foods were misclassiﬁed due to limited food choices in the CNF and a lack of brand names in the database - Lack of information for NOVA classification: CANDAT, our nutrient analysis software, does not allow us to disaggregate mixed foods based on recipes, so it is possible that some UPF used in culinary preparations, such as margarine used in making a traditional First Nations’ quick bread called bannock, may have been included in the fresh and minimally processed foods - Inherent classification problems: Since the NOVA food classiﬁcation does not group foods according to their nutritional quality, but rather based on the degree and nature of processing, some groupings might appear unusual from a nutritional perspective - Fewer studies assessing G1 and G3 compared to G4: future analyses might identify the MF that, when consumed in combination with TF, will contribute to a diet for First Nations peoples |
| Bonaccio et al., 2022^5^ | - Moreover, the FFQ used in this study, like most FFQs used in large scale prospective cohort studies,18 19 was not originally developed to assess the degree of food processing, so many food items were not included (for example, pre-prepared dishes, energymbars, slimming products). Although no one gold standard for applying the NOVA categorisation exists, we recognise that FFQs may not cover the full spectrum of foods consumed, including ultra-processed foods, owing to the limited number of predefined food lists and the lack of supporting information on cooking methods, ingredients, eating place, and the brand names of the packaged foods which would be extremely useful in identifying ultra- processed foods. | - Problems with dietary data recording: FFQ used in this study, like most FFQs used in large scale prospective cohort studies,18 19 was not originally developed to assess the degree of food processing, so many food items were not included (for example, pre-prepared dishes, energymbars, slimming products - Lack of information for NOVA classification: no one gold standard for applying the NOVA categorisation exists, we recognise that FFQs may not cover the full spectrum of foods consumed, including ultra-processed foods, owing to the limited number of predefined food lists and the lack of supporting information on cooking methods, ingredients, eating place, and the brand names of the packaged foods which would be extremely useful in identifying ultra- processed foods. |
| Cediel et al., 2020^6^ | - In addition, although information indicative of food processing, such as place of meals and product brands, was collected, these data were missing for some food items and thus may have led to errors in food classification. | - Lack of information for NOVA classification: although information indicative of food processing, such as place of meals and product brands, was collected, these data were missing for some food items and thus may have led to errors in food classification |
| Chen et al., 2018^7^ | - The classification of UPF by NOVA has come to be a reference method. However, its cross-cultural extrapolation requires assumptions that may not be valid or feasible, with consequent misclassification of food groups. | - Cross-cultural extrapolation: cross-cultural extrapolation requires assumptions that may not be valid or feasible, with consequent misclassification of food groups. |
| da Rocha et al., 2023^8^ | - FFQ are not expected to collect information about food processing; thus, misclassification of some food items cannot be discarded. | - Problems with dietary data recording: FFQ are not expected to collect information about food processing; thus, misclassification of some food items cannot be discarded |
| da Silva et al., 2021^9^ | - Finally, the lack of available information in terms of quantities and manufacturing of industrial ingredients or food grade chemicals such as preservatives and dyes has meant that it has not been possible to account for their effects or to include them in this study. Accounting for these ingredients would mainly increase the effects of G4 products. | - Lack of information for NOVA classification: the lack of available information in terms of quantities and manufacturing of industrial ingredients or food grade chemicals such as preservatives and dyes has meant that it has not been possible to account for their effects or to include them in this study. Accounting for these ingredients would mainly increase the effects of G4 products. |
| Delgado-Rodríguez et al., 2023^10^ | - NOVA proponents should also consider the advisability of further refining their food processing classification by either further dividing or regrouping foods across the UP and P categories or adding greater nuance to their descriptions of UP and P foods. - Regarding difficulties differentiating between UP and P foods, we acknowledge that our design probably inherited some of the intrinsic shortcomings of the NOVA classification system, which can be imperfect but nonetheless largely reliable and valid | - Inherent classification problems: further refining their food processing classification by either further dividing or regrouping foods across the UP and P categories or adding greater nuance to their descriptions of UP and P foods |
| Detopoulou et al., 2021^11^ | - Regarding UPF intake, the NOVA food classification system was used, but some argue that it has “little additive value” over nutrient evaluation. Moreover, it is not always clear whether a food is UPF or not. For example, bread made from wheat flour, water, salt and yeast, constitutes a processed food, while if it contains emulsifiers or colors is considered a UPF. | - Inherent classification problems: it has “little additive value” over nutrient evaluation - Food group specific classification problems: bread made from wheat flour, water, salt and yeast, constitutes a processed food, while if it contains emulsifiers or colors is considered a UPF |
| Dickie et al., 2022^12^ | - However, NOVA could need some small technical adjustments to strengthen its applicability to policy actions targeting individual foods. For example, foods high in added sugar or salt, such as sweetened beverages or beef jerky, classified as unhealthy by other NCSs, can be classified as healthy by NOVA (Table 5). Furthermore, some foods classified as healthy by the ADGs, such as cheese, yogurts, and breads, can be classified as ultraprocessed by NOVA. However, these recommended healthy foods could potentially be reformulated by removing the cosmetic additives and industrial ingredients (markers of ultraprocessing) that transform the textural and sensory properties of foods, and this could be encouraged with regulation. This has been described as “wholefood reformulation,” whereby food innovations result in a range of new minimally processed (NOVA group 3) products. | - Inherent classification problems: NOVA could need some small technical adjustments to strengthen its applicability to policy actions targeting individual foods - "Blindness" for foods high in fat, salt and/or sugar: foods high in added sugar or salt, such as sweetened beverages or beef jerky, classified as unhealthy by other NCSs, can be classified as healthy by NOVA - Food group specific classification problems: some foods classified as healthy by the ADGs, such as cheese, yogurts, and breads, can be classified as ultraprocessed by NOVA |
| García et al., 2023^13^ | - The first limitation for this study is the lack of a validated questionnaire that measures UPF consumption. The use of the FFQ provides an overview, but it is not precisely assessing UPF consumption. For example, fruit juices, milkshakes, meatballs, hamburgers and pizza can be consumed as artisanal, but here they were also considered industrial and classified as ultraprocessed products. FFQ does not distinguish between plain, sweetened, or flavored varieties of yogurts and whole-grain cereals so these foods were considered to belong to unprocessed or minimally processed foods group. | - Problems with dietary data recording: lack of a validated questionnaire that measures UPF consumption - Food group specific classification problems: (1) fruit juices, milkshakes, meatballs, hamburgers and pizza can be consumed as artisanal, but here they were also considered industrial and classified as ultraprocessed products, (2) FFQ does not distinguish between plain, sweetened, or flavored varieties of yogurts and whole-grain cereals so these foods were considered to belong to unprocessed or minimally processed foods group |
| Grech et al., 2022^14^ | - A limitation of this study is that processing is not always adequately captured in the product description in the nutrient composition database, making the NOVA classiﬁcation difﬁcult to apply. Bread is one category where this is problematic as it is not possible to distinguish artisanal or homemade breads and mass-produced breads. To minimise differences, the NOVA coding system of Australian foods emulated that of previous research where foods were agreed upon by at least two researchers who applied expert knowledge in the Australian food supply, but there may still be some arbitrary misclassiﬁcation of foods. | - Lack of information for NOVA classification: processing is not always adequately captured in the product description in the nutrient composition database - Food group classification problems: bread is one category where this is problematic as it is not possible to distinguish artisanal or homemade breads and mass-produced breads - Inherent classification problems: there may still be some arbitrary misclassiﬁcation of foods by experts |
| Gupta et al., 2021^15^ | - The ambiguity in the definition of NOVA classification may have resulted in some misclassification, though this has been minimised by employing two independent researchers to assign food items. | - Inherent classification problems: ambiguity in the definition of NOVA classification |
| Juul et al., 2019^16^ | - Misclassification of foods may have occurred for items for which insufficient details were available to determine processing level with certainty. | - Lack of information for NOVA classification: Misclassification of foods may have occurred for items for which insufficient details were available to determine processing level with certainty. |
| Juul et al., 20211^17^ | - Processing level may have been misclassified as certain FFQ line items included foods of potentially different processing levels. However, we attempted to minimise misclassification by basing assumptions of processing level of FFQ items on the current literature and on actual consumption data from adults in NHANES 2001–2002 with similar demographics to the FOS cohort. The Harvard FFQ differentiates between homemade and ready-made cookies, cakes, pies and pastries; distinguishes between homemade and commercially prepared fried foods and allow respondents to specify the type and brand of breakfast cereals consumed, which enabled a more precise classification of these foods. | - Problems with dietary data recording: Processing level may have been misclassified as certain FFQ line items included foods of potentially different processing levels |
| Lavigne-Robichaud et al., 2018^18^ | - Finally, the 24 h recall was not designed to use the NOVA classification; therefore, some foods could have been misclassified due to lack of sufficient details. | - Problems with dietary data recording: 24 h recall was not designed to use the NOVA classification; therefore, some foods could have been misclassified due to lack of sufficient details |
| Marchese et al., 2021^19^ | - A potential limitation is that the 24-h recall instrument and the food composition tables were not designed for evaluating food based on the level of processing. Hence, some items may have been misclassified. | - Problems with dietary data recording: limitation is that the 24-h recall instrument not designed for evaluating food based on the level of processing - Lack of information for NOVA classification: food composition tables were not designed for evaluating food based on the level of processing |
| Martínez-Perez, 2021^20^ | - Althought the suitability of FFQs for evaluating dietary consumption in large epidemiological studies is undeniable, it does present limitations regarding recall bias and nutrient intake estimation. The latter is even more pronounced when highly processed foods are considered, as there is a wide variety and they are not present in food composition tables. Therefore, it would be worth considering the inclusion of food processing-criteria when designing FFQs for studies aimed to investigate the role of UPFs on health. - Assumptions about dietary patterns, cooking methods and food composition and processing were made to ensure consistency when applying the criteria of different systems, but foods may fall into different categories when other dietary patterns are considered. | - Lack of information for NOVA classification: (1)when highly processed foods are considered, as there is a wide variety and they are not present in food composition tables, (2) assumptions about dietary patterns, cooking methods and food composition and processing were made to ensure consistency |
| Robert et al., 2022^21^ | - Finally, the UPF outcome overlapped with several of the food groups assessed (e.g., dairy desserts or processed meat). | - Food group specific classification problems: UPF outcome overlapped with several of the food groups assessed (e.g., dairy desserts or processed meat) |
| Rossato et al., 2023^22^ | - Lastly, the fact that food intake was assessed using FFQ also indicates a potential limitation.First, the list of foods included in the FFQ is limited, and it might not incorporate the usual food-processing categories. Second, categorizing food items into processing categories relies on the investigator’s classiﬁcation, which might lead to misclassiﬁcation or conﬁrmation biases. Building FFQs to assess food-processing categories is a plausible option to mitigate the ﬁrst FFQ limitation for future studies; however, it would not mitigate this FFQ food list limitation for large ongoing prospective studies. To mitigate misclassiﬁcation and conﬁrmation biases, standardizing food classiﬁcation using food composition tables should remove personal inﬂuences or propensity to decision-making errors and ensure comparability among study results. | - Problems with dietary data recording: First, the list of foods included in the FFQ is limited, and it might not incorporate the usual food-processing categories. - Inherent classification problems: categorizing food items into processing categories relies on the investigator’s classiﬁcation, which might lead to misclassiﬁcation or conﬁrmation biases |
| Ruggiero et al., 2021^23^ | - Finally, the NOVA classification we used is still debated, mainly because of its equivocal definition of UPF and also because it has been revised and refined over time, however, its usefulness in nutrition research has been widely acknowledged. | - Inherent classification problems: the NOVA classification we used is still debated, mainly because of its equivocal definition of UPF |
| Salome et al., 2021^24^ | - One limitation of this study may concern our use of the NOVA classifcation, where the ultra-processed foods group is very broad and includes markedly differing foods that have been ascribed to this group for different reasons (presence of additives or highly technological food processing). Some food items are also classifed according to their method of preparation, requiring imputations that could be questionable (i.e. systematically considering foods from fast-food restaurant as industrially processed). However, the NOVA classification has been largely employed in the literature and its use ofers a clear reference when discussing our results. Another limitation concerns our food composition database. Indeed, a food item with the same overall label was assigned to diferent NOVA categories depending on its origin despite there being a lack of data regarding its composition. Regarding the study size and generalizability, the present study took advantage of a representative setting in a country with western dietary patterns and an important share of industrial foods, with a diversity of patterns. High consumers of MPF and UPF appear to have dietary patterns comparable to those reported in studies using other samples. High consumers of PF, which represent a more traditional pattern might be less generalizable, but data are lacking for further analysis since this NOVA category has been much less studied. Last, although the data came from the latest French national representative survey (in 2014–2015), they may not have been sufciently recent to refect current dietary transitions and the increased use of plant-based products. | - Inherent classification problems: (1) ultra-processed foods group is very broad and includes markedly differing foods that have been ascribed to this group for different reasons (presence of additives or highly technological food processing). Some food items are also classifed according to their method of preparation, requiring imputations that could be questionable (i.e. systematically considering foods from fast-food restaurant as industrially processed) - Lack of information for NOVA classification: a food item with the same overall label was assigned to different NOVA categories depending on its origin despite there being a lack of data regarding its composition - Fewer studies assessing G1 and G3 compared to G4: High consumers of MPF and UPF appear to have dietary patterns comparable to those reported in studies using other samples. High consumers of PF, which represent a more traditional pattern might be less generalizable, but data are lacking for further analysis since this NOVA category has been much less studied. - Food group specific classification problems: latest French national representative survey (in 2014–2015), they may not have been sufciently recent to refect current dietary transitions and the increased use of plant-based products |
| Shim et al., 2020^25^ | - Although the concepts and guidelines of the NOVA food classiﬁcation system are well known, some foods are still likely to be interpreted and classiﬁed differently. For this reason, the present study made a special effort to classify the foods reliably. Three researchers independently categorized foods according to the NOVA classiﬁcation system and then, items for which there were inconsistencies were resolved by discussion. | - Inherent classification problems: some foods are still likely to be interpreted and classiﬁed differently |
| Siqueira et al. 2020^26^ | - Third, the study was based on foods and not total diets. However, the results of NOVA studies for diets are also inconsistent. | - Dietary level of analysis: the study was based on foods and not total diets |
| Sneed et al., 2023^27^ | - We were unable to determine the composition of ingredients used to make mixed dishes (e.g., homemade vs. ready-made). This may have resulted in the misclassiﬁcation of calories, leading us to potentially under- or overestimate the caloric contributions of some Nova categories. - It is recommended that future researchers employ a double-coding methodology followed by expert adjudication of discordant food items when conducting Nova categorization | - Lack of information for NOVA classification: lack of information on ingredients of mixed dishes (hommade vs ready-made) - Inherent classification problems: It is recommended that future researchers employ a double-coding methodology followed by expert adjudication of discordant food items when conducting Nova categorization |
| Spiteri et al., 2018^28^ | - It is possible that some product misclassifications occurred, however, having a second, independent researcher check all classifications and a consensus approach when there were differences should have limited any misclassifications. | - Inherent classification problems: some product misclassifications occurred |
| Vandevijvere et al., 2020^29^ | - Home-prepared dishes and meals were disaggregated into ingredients, and those ingredients were categorized according to the NOVA classification. For some home prepared composite foods, this disaggregation was not possible (e.g., some milk-based desserts, cakes, pies and pastries, some soups and sauces), but they represented only about 0.9% of all foods consumed. Such foods were classified as processed. Alcohol was not categorized using NOVA and was kept as a separate group. | - Lack of information for NOVA classification:For some home prepared composite foods, this disaggregation was not possible (e.g., some milk-based desserts, cakes, pies and pastries, some soups and sauces), |
| Vellinga  et al., 2023^30^ | - Dietary data was collected using a FFQ that was not designed to collect data on the degree of food processing. For instance, misclassification could occur because, according to the FFQ, it was not clear if tomato sauce was homemade (unprocessed or minimally processed food) or ready-to-eat (processed food). - Although the NOVA classification is most used to classify foods according to their degree of processing, insufficient standardization of the application of the methodology makes food classification with NOVA difficult and can lead to confusion and subjective coding of foods and drinks.The influence of differences in dietary assessment method, application of food processing application methods such as NOVA, and time period might have influenced the results and cannot be disentangled. | - Problems with dietary data recording: a FFQ that was not designed to collect data on the degree of food processing - Inherent classification problems: insufficient standardization of the application of the methodology makes food classification with NOVA difficult and can lead to confusion and subjective coding of foods and drinks. |
| Vellinga et al., 2022^31^ | - It should be noted that classification systems such as NOVA conceptually differ from processing level concepts in food science technology. The use of NOVA enables comparison with other studies. Nevertheless, different definitions of UPFD and insufficient standardization make food classification with NOVA difficult and can lead to confusion and subjective recoding of national food consumption data-bases - Although we had to make assumptions as well, food consumption data in our study was collected with a great level of detail and systematically stored. We were, therefore, able to systematically categorize foods according to NOVA with little inconsistencies or subjective classifications. - The difficulty of classifying bread according to NOVA has been addressed previously as terminology such as artisanal bread, sliced or unsliced, mass-produced is used, but their exact interpretation is not self-evident | - Inherent classification problems: different definitions of UPFD and insufficient standardization make food classification with NOVA difficult and can lead to confusion and subjective recoding of national food consumption data-bases - Food group specific classification problems: The difficulty of classifying bread according to NOVA has been addressed previously as terminology such as artisanal bread, sliced or unsliced, mass-produced is used, but their exact interpretation is not self-evident |

References:

S1 Abreu S, Liz Martins M. Cross-Classification Analysis of Food Products Based on Nutritional Quality and Degree of Processing. Nutrients. 2023;15(14):3117. doi:10.3390/nu15143117.

S2 Aceves-Martins M, Bates RL, Craig LC, et al. Nutritional quality, environmental impact and cost of ultra-processed foods: a UK food-based analysis. Int. J. Environ. Res. Public Health. 2022;19(6):3191. doi:10.3390/ijerph19063191

S3 Barrett EM, Gaines A, Coyle DH, et al. Comparing product healthiness according to the Health Star Rating and the NOVA classification system and implications for food labelling systems: An analysis of 25 486 products in Australia. Nutr Bull. 2023;48(4):523-534. doi:10.1111/nbu.12640. Epub 2023 Oct 28

S4 Batal M, Johnson-Down L, Moubarac JC, et al. Quantifying associations of the dietary share of ultra-processed foods with overall diet quality in First Nations peoples in the Canadian provinces of British Columbia, Alberta, Manitoba and Ontario. Public Health Nutr. 2018;21(1):103-113. doi:10.1017/S1368980017001677

S5 Bonaccio M, Di Castelnuovo A, Ruggiero E, et al. Joint association of food nutritional profile by Nutri-Score front-of-pack label and ultra-processed food intake with mortality: Moli-sani prospective cohort study. BMJ. 2022,378. doi:10.1136/bmj-2022-070688

S6 Cediel G, Reyes M, Corvalán C, et al. Ultra-processed foods drive to unhealthy diets: evidence from Chile. Public Health Nutr. 2021;24(7):1698-1707. doi:10.1017/S1368980019004737

S7 Chen YC, Huang YC, Lo YTC, et al. Secular trend towards ultra-processed food consumption and expenditure compromises dietary quality among Taiwanese adolescents. Food Nutr Res. 2018,62. doi:10.29219/fnr.v62.1565

S8 da Rocha BRS, Rico-Campà A, Romanos-Nanclares A, et al. Adherence to Mediterranean diet is inversely associated with the consumption of ultra-processed foods among Spanish children: The SENDO project. Public Health Nutr. 2021;24:3294-3303. doi:10.1017/S1368980020001524

S9 da Silva JT, Garzillo JMF, Rauber F, et al. Greenhouse gas emissions, water footprint, and ecological footprint of food purchases according to their degree of processing in Brazilian metropolitan areas: a time-series study from 1987 to 2018. Lancet Planetary Health. 2021,5:775-785.

S10 Delgado-Rodríguez R, Moreno-Padilla M, Moreno-Domínguez S, Cepeda-Benito A. Food addiction correlates with emotional and craving reactivity to industrially prepared (ultra-processed) and home-cooked (processed) foods but not unprocessed or minimally processed foods. Food Qual Prefer. 2023;110:104961. doi:10.1016/j.foodqual.2023.104961

S11 Detopoulou P, Dedes V, Pylarinou I, et al. Dietary acid load is associated with waist circumference in university students with low adherence to the Mediterranean diet: The potential role of ultra-processed foods. Clin Nutr ESPEN. 2023;56:43-51. doi:10.1016/j.clnesp.2023.05.005

S12 Dickie S, Woods J, Machado P, Lawrence M. Nutrition classification schemes for informing nutrition policy in Australia: nutrient-based, food-based, or dietary-based?. Curr Dev Nutr. 2022;6(8):112. doi:10.1093/cdn/nzac112

S13 García S, Pastor R, Monserrat-Mesquida M, et al. Ultra-processed foods consumption as a promoting factor of greenhouse gas emissions, water, energy, and land use: A longitudinal assessment. Sci Total Environ. 2023;891:164417. doi:10.1016/j.scitotenv.2023.164417

S14 Grech A, Rangan A, Allman-Farinelli M, et al. A Comparison of the Australian Dietary Guidelines to the NOVA Classification System in Classifying Foods to Predict Energy Intakes and Body Mass Index. Nutrients. 2022;14:3942. doi:10.3390/nu14193942

S15 Gupta S, Rose CM, Buszkiewicz J, et al. Characterising percentage energy from ultra-processed foods by participant demographics, diet quality and diet cost: Findings from the Seattle Obesity Study (SOS) III. BJN. 2021;126:773-781. doi:10.1017/S0007114520004705

S16 Juul F, dos Santos Simões B, Litvak J, et al. Processing level and diet quality of the US grocery cart: is there an association?. Public Health Nutr. 2019;22:2357-2366. doi:10.1017/S1368980019001344

S17 Juul F, Lin Y, Deierlein AL, et al. Trends in food consumption by degree of process. BJN. 2021;126:1861-1871. doi:10.1017/S000711452100060X

S18 Lavigne-Robichaud M, Moubarac JC, Lantagne-Lopez S, et al. Diet quality indices in relation to metabolic syndrome in an Indigenous Cree (Eeyouch) population in northern Québec, Canada. Public Health Nutr. 2018;21(1):172-180. doi:10.1017/S136898001700115X

S19 Marchese L, Livingstone KM, Woods JL, et al. Ultra-processed food consumption, socio-demographics and diet quality in Australian adults. Public Health Nutr. 2022;25(1):94-104. doi:10.1017/S1368980021003967

S20 Martinez-Perez N, Arroyo-Izaga M. Availability, nutritional profile and processing level of food products sold in vending machines in a Spanish public university. Int. J. Environ. Res. Public Health. 2021;18(13):6842. doi:10.3390/ijerph18136842

S21 Robert M, Shankland R, Bellicha A, et al. Associations between resilience and food intake are mediated by emotional eating in the NutriNet-Santé Study. Journal of Nutr. 2022;152:1907-1915. doi:10.1093/jn/nxac124

S22 Rossato SL, Khandpur N, Lo CH, et al. Intakes of unprocessed and minimally processed and Ultraprocessed food are associated with diet quality in female and male health professionals in the United States: A prospective analysis. J Acad Nutr Diet. 2023;123:1140-1151. doi:10.1016/j.jand.2023.03.011

S23 Ruggiero E, Esposito S, Costanzo S, et al. Ultra-processed food consumption and its correlates among Italian children, adolescents and adults from the Italian Nutrition & Health Survey (INHES) cohort study. Public Health Nutr. 2021;24:6258-6271. doi:10.1017/S1368980021002767

S24 Salomé M, Arrazat L, Wang J, et al. Contrary to ultra-processed foods, the consumption of unprocessed or minimally processed foods is associated with favorable patterns of protein intake, diet quality and lower cardiometabolic risk in French adults (INCA3). Eur J Nutr. 2021;60:4055-4067. doi:10.1007/s00394-021-02576-2

S25 Shim JS, Shim SY, Cha HJ, et al. Association between ultra-processed food consumption and dietary intake and diet quality in Korean adults. JAcad Nutr Diet. 2022;122:583-594. doi:10.1016/j.jand.2021.07.012

S26 Siqueira KB, Borges CA, Binoti ML, et al. Nutrient density and affordability of foods in Brazil by food group and degree of processing. Public Health Nutr. 2021;24(14):4564-4571. doi:10.1017/S1368980020004358

S27 Sneed NM, Ukwuani S, Sommer EC, et al. Reliability and validity of assigning ultraprocessed food categories to 24-h dietary recall data. Am J Clin Nutr. 2023;117:182-190. doi:10.1016/j.ajcnut.2022.10.016.

S28 Spiteri SA, Olstad DL, Woods JL. Nutritional quality of new food products released into the Australian retail food market in 2015–is the food industry part of the solution? BMC Public Health, 2018;18:1-10. doi:10.1186/s12889-018-5127-0

S29 Vandevijvere S, Pedroni C, De Ridder K, Castetbon, K. The cost of diets according to their caloric share of ultraprocessed and minimally processed foods in Belgium. Nutrients. 2020;12:2787. doi:10.3390/nu12092787

S30 Vellinga RE, van den Boomgaard I, Boer JM, Different Levels of Ultraprocessed Food and Beverage Consumption and Associations with Environmental Sustainability and All-cause Mortality in EPIC-NL. Am J Clin Nutr. 2023;118(1):103-113. doi:10.1016/j.ajcnut.2023.05.02

S31 Vellinga RE, van Bakel M, Biesbroek S, et al. Evaluation of foods, drinks and diets in the Netherlands according to the degree of processing for nutritional quality, environmental impact and food costs. BMC Public Health. 2022;22: 877. doi:10.1186/s12889-022-13282-x
