# Supplementary material for: Fifteen Years of NOVA Food-Processing Classification: “Friend or Foe” Among Sustainable Diet Indicators? A Scoping Review
Source: Nutr Rev. 2025 Jan 23;83(4):771–91. doi: 10.1093/nutrit/nuae207 (PMC11894255; doi:10.1093/nutrit/nuae207)
Supplement: nuae207_Supplementary_Data [file nuae207_supplementary_data.zip › nuae207_Supplementary_Data/Table_S4_Key_findings_related_to_NOVA.docx]

| Reference | Key conclusions related to NOVA | Identified topics |
| --- | --- | --- |
| Abreu & Martins, 2023^1^ | - Many Ultra-Processed Foods (UPFs) found in all Nutri-Score categories - Food manufacturers reformulate products to achieve better Nutri-Score - A weak positive correlation was found between Nutri-Score and NOVA classification - Food processing and nutritional quality should both be considered in labeling - Reformulation includes reducing sugar, fat, salt, and increasing fiber content | - NOVA alignment with other SDI(s): (1) Many Ultra-Processed Foods (UPFs) found in all Nutri-Score categories (2) A weak positive correlation was found between Nutri-Score and NOVA classification - Policy implications: Food processing and nutritional quality should both be considered in labeling |
| Aceves-Martins et al., 2022^2^ | - The high variability in greenhouse gas emissions and cost across food groups and processing levels offer opportunities for food swaps representing the healthiest, greenest, and most affordable options. - This is an important issue to consider as our analysis indicated that ultra-processed and processed foods were cheaper than minimally processed foods, regardless of their total fat, salt, and/or sugar content, which may underpin the increased consumption levels of ultra-processed foods | - Sustainable dietary implications related to NOVA classification: (1) The high variability in greenhouse gas emissions and cost across food groups and processing levels offer opportunities for food swaps representing the healthiest, greenest, and most affordable options. (2) It is an important issue to consider as our analysis indicated that ultra-processed and processed foods were cheaper than minimally processed foods, regardless of their total fat, salt, and/or sugar content, which may underpin the increased consumption levels of ultra-processed foods |
| Angelino et al., 2023^3^ | - Overall, these results suggest that the NOVA classification partially overlaps with systems based on the nutritional quality of foods. The lower nutritional quality of NOVA 4 foods may at least partially explain the association found between the consumption of ultra-processed foods and the risk of chronic diseases. - We argue whether both the NOVA and Nutri-Score systems may converge in a unique definition of the healthiness of the product. | - NOVA alignment with other SDI(s): (1) Overall, these results suggest that the NOVA classification partially overlaps with systems based on the nutritional quality of foods (2) The lower nutritional quality of NOVA 4 foods may at least partially explain the association found between the consumption of ultra-processed foods and the risk of chronic diseases. |
| Baldridge et al., 2019^4^ | - When examining results by category, the level of processing did not always correlate with products considered healthy using the HSR | - NOVA alignment with other SDI(s): when examining results by category, the level of processing did not always correlate with products considered healthy using the HSR |
| Barrett et al., 2023^5^ | - Our analysis strengthens the evidence for the similarities and differences in product healthiness according to a nutrient-based classification system and a processing-based classification system. Although the systems' classifications align for the majority of food and beverage products, the discordance found for some product categories indicates the potential for confusion if systems are deployed alongside each other within food policies | - Policy implications: Our analysis strengthens the evidence for the similarities and differences in product healthiness according to a nutrient-based classification system and a processing-based classification system. Although the systems' classifications align for the majority of food and beverage products, the discordance found for some product categories indicates the potential for confusion if systems are deployed alongside each other within food policies |
| Batal et al., 2018^6^ | - UPF was prevalent in First Nations diets. Efforts to curb UPF consumption and increase intake of traditional First Nations foods and other fresh or minimally processed foods would improve diet quality and health in First Nations peoples. | - Sustainable dietary implications related to NOVA classification: UPF was prevalent in First Nations diets. Efforts to curb UPF consumption and increase intake of traditional First Nations foods and other fresh or minimally processed foods would improve diet quality and health in First Nations peoples |
| Batista et al., 2022^7^ | - More than 95% of the foods available in the stores assessed by this study were nutritionally unbalanced, exceeding the limits set by the PAHO for at least one critical nutrient and illustrating the mass exposure of consumers to unhealthy foods | - Policy implications: More than 95% of the foods available in the stores assessed by this study were nutritionally unbalanced, exceeding the limits set by the PAHO for at least one critical nutrient and illustrating the mass exposure of consumers to unhealthy foods |
| Baye & Yaregal, 2023^8^ | - Unlike UPF and WDDS alone, GDQS was able to predict both nutrient adequacy and unhealthy dietary practices. - The diet quality of women of reproductive age (WRA) in Addis Ababa is low in diversity, possibly exposing them to a higher risk of nutrient inadequacy and non-communicable diseases as reflected by the low GDQS. Understanding what drives food and dietary choices in urban settings is urgently needed. | - NOVA alignment with other SDI(s): (1) GDQS was positively associated with WDDS, age and skipping breakfast, whereas it was negatively associated with eating out of home and UPF. The GDQS was, however, not associated with wealth.Unlike UPF and WDDS alone, GDQS was able to predict both nutrient adequacy and unhealthy dietary practices. - Policy implications: The diet quality of women of reproductive age (WRA) in Addis Ababa is low in diversity, possibly exposing them to a higher risk of nutrient inadequacy and non-communicable diseases as reflected by the low GDQS. - Recommended research direction: Understanding what drives food and dietary choices in urban settings is urgently needed. |
| Berardy et al., 2020^9^ | - Ultra-processed foods had the highest GWP as expected, but processed foods had the highest land use, and unprocessed or minimally processed foods had the highest water consumption. - Plant-based foods had the overall lowest environmental impacts, while animal-based foods had the overall highest environmental impacts. This held when comparing common sources of protein on a protein content functional unit basis. Finally, although ultra-processed foods had the highest GWP, processed foods had the highest land use, and unprocessed foods had the highest water use, implying that the production of raw ingredients drives impacts more than their processing. | - NOVA alignment with other SDI(s): (1) Ultra-processed foods had the highest GWP as expected, but processed foods had the highest land use, and unprocessed or minimally processed foods had the highest water consumption. (2) Finally, although ultra-processed foods had the highest GWP, processed foods had the highest land use, and unprocessed foods had the highest water use, implying that the production of raw ingredients drives impacts more than their processing. |
| Blanchet et al., 2020^10^ | - Although TF contributed to a low proportion of the daily energy intake of Syilx adults, similar to previous studies with other Indigenous populations in Canada, TF - consumption was significantly associated with enhanced nutrient intakes, higher HEI-C scores, lower %E from UPP, and higher %E from fresh and minimally processed foods. Participants who ate TF may have been more likely to make home-prepared meals and less likely to purchase UPP | - NOVA alignment with other SDI(s): Although TF contributed to a low proportion of the daily energy intake of Syilx adults, similar to previous studies with other Indigenous populations in Canada, TF consumption was significantly associated with enhanced nutrient intakes, higher HEI-C scores, lower %E from UPP, and higher %E from fresh and minimally processed foods. - Policy implications Participants who ate TF may have been more likely to make home-prepared meals and less likely to purchase UPP |
| Bleiweiss-Sande et al., 2019^11^ | - Current classification systems may not identify high-nutrient foods for children - UNC and Nova systems had the highest agreement - Higher added sugar and sodium categories were significant predictors of highly processed foods (but not other examined nutrients) | - NOVA alignment with other SDI(s): (1) UNC and Nova systems had the highest agreement (2) Higher added sugar and sodium categories were significant predictors of highly processed foods (but not other examined nutrients) - Policy implications: Current classification systems may not identify high-nutrient foods for children |
| Bonaccio et al., 2022^12^ | - Adults with the lowest quality diet, measured by FSAm-NPS dietary index and high ultra-processed food consumption, have the highest risk of all-cause and cardiovascular mortality. - Diets composed of higher FSAm-NPS and a large dietary share of ultra-processed foods are associated with higher hazards of all-cause and cardiovascular disease mortality. - Increased ultra-processed food consumption, not the FSAm-NPS dietary index, is an independent risk factor for mortality due to ischaemic heart disease/cerebrovascular disease and other causes. - The associations of the FSAm-NPS dietary index with mortality were significantly attenuated when considering both nutrient balance and food processing, while estimations for ultra-processed food intake remained almost unchanged. - These findings suggest that highly processed foods are associated with poor health outcomes independently of their low nutritional composition, but not the other way around. | - NOVA alignment with other SDI(s): (1) Adults with the lowest quality diet, measured by FSAm-NPS dietary index and high ultra-processed food consumption, have the highest risk of all-cause and cardiovascular mortality. (2) These findings suggest that highly processed foods are associated with poor health outcomes independently of their low nutritional composition, but not the other way around. - Sustainable dietary implications related to NOVA classification: Diets composed of higher FSAm-NPS and a large dietary share of ultra-processed foods are associated with higher hazards of all-cause and cardiovascular disease mortality. |
| Braesco et al., 2022^13^ | - The consistency of food assignments varied for different foods. However, overall consistency among evaluators was low. Even with ingredient information available, the results suggest that the current NOVA criteria do not support robust and functional food assignments. | - Critics on NOVA: NOVA criteria do not support robust and functional food assignments. |
| Cediel et al., 2021^14^ | - As the consumption of ultra-processed foods increased, the intake of other food groups decreased. - Ultra-processed foods exhibit unhealthier nutrient profiles compared to other food groups: they contain higher levels of NCD-promoting nutrients such as energy, free sugars, and saturated fats. In contrast, they have lower levels of NCD-protective nutrients such as fiber and potassium. - This highlights the negative impact of ultra-processed foods on overall dietary quality. | - NOVA alignment with other SDI(s): Ultra-processed foods exhibit unhealthier nutrient profiles compared to other food groups: they contain higher levels of NCD-promoting nutrients such as energy, free sugars, and saturated fats. In contrast, they have lower levels of NCD-protective nutrients such as fiber and potassium. - Sustainable dietary implications related to NOVA classification: this highlights the negative impact of ultra-processed foods on overall dietary quality. |
| Chen et al., 2018^15^ | - The observed trends in expenditure on food suggest that lower UPF costs influenced food choices during this period. Increasing UPF intake and expenditure was associated with poor dietary quality - The adolescents investigated in this study who consumed more UPFs and fewer original foods had less healthy nutrient and cardiometabolic risk profiles without having incurred additional expenditure on foods. - These findings indicate that national health system planning, and policy-making, should be more responsive to significant changes occurring in the food system, related nutritional and health impact trajectories, and cost factors in food choices. | - NOVA alignment with other SDI(s): The observed trends in expenditure on food suggest that lower UPF costs influenced food choices during this period. Increasing UPF intake and expenditure was associated with poor dietary quality - Policy implications: These findings indicate that national health system planning, and policy-making, should be more responsive to significant changes occurring in the food system, related nutritional and health impact trajectories, and cost factors in food choices. |
| Cooper et al., 2017^16^ | - At the optimal cutoff point of four stars, the HSR has a high sensitivity but a low speciﬁcity to correctly classify healthy packaged dairy beverages, as deﬁned by NOVA. - Our results show that the nutrient proﬁling model used to classify dairy beverages (category 1D) discriminates between non-ultra-processed (that is, healthy) and ultra-processed (that is, less healthy) foods and, therefore, conﬁrms the construct validity of the HSR for dairy beverages. | - NOVA alignment with other SDI(s): the HSR has a high sensitivity but a low speciﬁcity to correctly classify healthy packaged dairy beverages, as deﬁned by NOVA |
| da Rocha et al., 2021^17^ | - High adherence to the Mediterranean Diet linked to an 8.5% decrease in energy intake from ultra-processed foods - Variability in free sugar intake explained by variability of ultra-processed food consumption - Yogurts classified as ultra-processed foods showed a slightly lower reduction in energy intake - Correlation and regression analyses favored the classification of yogurts as ultra-processed foods - Importance of confronting the contribution of sweetened dairy products to variability in sugar intake | - Sustainable dietary implications related to NOVA classification: (1) High adherence to the Mediterranean Diet linked to an 8.5% decrease in energy intake from ultra-processed foods (2) Importance of confronting the contribution of sweetened dairy products to variability in sugar intake - NOVA alignment with other SDI(s): Variability in free sugar intake explained by variability of ultra-processed food consumption |
| da Silva et al., 2021^18^ | - The environmental effects of the Brazilian diet have increased over the past three decades along with increased effects from ultra-processed foods. This means that dietary patterns in Brazil are becoming potentially more harmful to human and planetary health. | - Sustainable dietary implications related to NOVA classification: The environmental effects of the Brazilian diet have increased over the past three decades along with increased effects from ultra-processed foods. This means that dietary patterns in Brazil are becoming potentially more harmful to human and planetary health. |
| Davidou et al., 2020^19^ | - Siga is not intended to substitute the NOVA classification but rather is intended to be a pragmatic tool and a rational, objective, and actionable score for retailers and the agro-food industry to improve the quality of both diets and processed foods in a more gradual or step-by-step manner. NOVA is necessary and sufficient for research and consumers. | - Recommendation to use NOVA with other SDI(s): Siga is not intended to substitute the NOVA classification but rather is intended to be a pragmatic tool and a rational, objective, and actionable score for retailers and the agro-food industry to improve the quality of both diets and processed foods in a more gradual or step-by-step manner - Supporting argument on NOVA: NOVA is necessary and sufficient for research and consumers. |
| de Las Heras-Delgado et al., 2023^20^ | - Most PBAPs in Spain are classified as Nutri-Score A and B but as processed and ultra-processed products by the NOVA system while their nutritional values significantly vary which justifies the need for a two-dimensional approach to assess the nutritional value of PBAPs. - PBAPs generally have a better nutrient profile than animal-based homologs, but cheese, fish, and meats PBAPs are more processed and have poorer profiles. | - NOVA alignment with other SDI(s): (1) Most PBAPs in Spain are classified as Nutri-Score A and B but as processed and ultra-processed products by the NOVA system while their nutritional values significantly vary which justifies the need for a two-dimensional approach to assess the nutritional value of PBAPs. (2) PBAPs generally have a better nutrient profile than animal-based homologs, but cheese, fish, and meats PBAPs are more processed and have poorer profiles |
| de Moraes et al., 2021^21^ | - Individuals on a 'Unhealthy' diet consume more energy and processed foods, they have a lower intake of nutrients like vitamins and fiber | - NOVA alignment with other SDI(s): Individuals on a 'Unhealthy' diet consume more energy and processed foods, they have a lower intake of nutrients like vitamins and fiber |
| Delgado-Rodríguez et al., 2023^22^ | - Our findings concur with previous suggestions that foods can be classified along a continuum of addictiveness potential, but our findings are the first to demonstrate that such classification might be accomplished by following the NOVA classification system. The findings also imply that nutrition experts may need to refine their NOVA classification system and, perhaps, even their healthy diet recommendations. - The dietary recommendation to avoid UP foods and encourage natural and home-cooked meals likely needs to deemphasize or qualify the extent to which consumption of P foods is recommended. | - Recommendation for the application of NOVA: dietary recommendation to avoid UP foods and encourage natural and home-cooked meals likely needs to deemphasize or qualify the extent to which consumption of P foods is recommended - NOVA alignment with other SDI(s): foods can be classified along a continuum of addictiveness potential, but our findings are the first to demonstrate that such classification might be accomplished by following the NOVA classification system |
| Derbyshire, 2019^23^ | - No statistically significant correlations were found between the number of ingredients and energy, saturated fat, total sugar, sodium, AOAC fiber and protein. The majority of UPFs identified were low in saturated fat (84 percent), low in sugar (80 percent), and a source of fiber (60 percent). Thirty-eight percent were low in salt and 30 percent were a source of protein. - Based on the findings of this commentary it is suggested that rigorous nutritional analyses should be conducted alongside 'processing loads'. Overarching and tautological definitions could lead to the public avoiding all UPFs and have unintended consequences such as fiber and micronutrient shortfalls. | - NOVA alignment with other SDI(s): No statistically significant correlations were found between the number of ingredients and energy, saturated fat, total sugar, sodium, Association of Analytical Chemists (AOAC) fiber and protein. - Policy implications: (1) Based on the findings of this commentary it is suggested that rigorous nutritional analyses should be conducted alongside 'processing loads'. Overarching and tautological definitions could lead to the public avoiding all UPFs and have unintended consequences such as fiber and micronutrient shortfalls (2) Quite often some of the ingredients used were herbs and spices, preservative or fortificants. This yields the question about whether it is fair to judge products based on ‘ingredient numbers’ when some ingredients listed have a functional role to play by improving safety, shelf-life and nutritional profile. |
| Detopoulou et al., 2023^24^ | - Dietary acid load (DAL) may be linked to central obesity, particularly in those with poor dietary quality - University students with low adherence to the Mediterranean Diet showed a positive association between DAL and waist circumference - Foods containing phosphates, like ultra-processed foods, sodas, and fast foods, may contribute to an acidogenic environment | - Sustainable dietary implications related to NOVA classification: Foods containing phosphates, like ultra-processed foods, sodas, and fast foods, may contribute to an acidogenic environment |
| Dickie et al., 2022^25^ | - Wide variation observed in the agreement of healthy/unhealthy food classification among NCSs - NOVA could serve as a benchmark for evaluating food's health potential but may need technical adjustments | - NOVA alignment with other SDI(s): Wide variation observed in the agreement of healthy/unhealthy food classification among NCSs - Recommendation for the application of NOVA: NOVA could serve as a benchmark for evaluating food's health potential but may need technical adjustments |
| Dinu et al., 2022^26^ | - A study in middle-aged Italian adults found an inverse relation between adherence to Mediterranean Diet (MD) and consumption of ultra-processed food (UPF) | - NOVA alignment with other SDI(s): A study in middle-aged Italian adults found an inverse relation between adherence to Mediterranean Diet (MD) and consumption of ultra-processed food (UPF) |
| Estell et al., 2021^27^ | - Exclusion of ultra-processed foods may lower intake of key nutrients, especially in at-risk groups. - Avoidance of ultra-processed grain foods could lead to significant decreases in nutrient intake in Australians. - Substituting (UPFs) fortified versions with non-fortified ones may result in decreased intake of iodine, folate, thiamin, and iron. - Classification of certain breads as non-UPF raises issues of nutritional equivalency, cost, and access. - Practicality and acceptability of recommended ultra-processed food replacements must be considered. | - Sustainable dietary implications related to NOVA classification: (1) Exclusion of ultra-processed foods may lower intake of key nutrients, especially in at-risk groups.(2) Avoidance of ultra-processed grain foods could lead to significant decreases in nutrient intake in Australians - Food groups specific problems (3) Classification of certain breads as non-UPF raises issues of nutritional equivalency, cost, and access. - Policy implications: (1) Practicality and acceptability of recommended ultra-processed food replacements must be considered. (2) Substituting (UPFs) fortified versions of ‘Bread and bread rolls’ and ‘breakfast cereals ready-to-eat’ with non-fortified ones may result in decreased intake of iodine, folate, thiamin, and iron. |
| Fardet & Rock, 2020^28^ | - Tending towards a 3V-based diet, while respecting local constraints, should allow preserving human health, the environment (greenhouse gas emissions, pollution, deforestation, etc.), small farmers, animal welfare and biodiversity, culinary traditions, and socioeconomics (including alleviation of public health cost). | - Policy implications: Tending towards a 3V-based diet, while respecting local constraints, should allow preserving human health, the environment (greenhouse gas emissions, pollution, deforestation, etc.), small farmers, animal welfare and biodiversity, culinary traditions, and socioeconomics (including alleviation of public health cost). |
| Fardet et al., 2017^29^ | - Degree of food processing affects satiety potential and glycemic impact - Glycemic glucose equivalent (GGE) may better reflect food processing than glycemic index (GI) - The relationship between food processing, satiety, and glycemic potential needs further study | - NOVA alignment with other SDI(s): (1) degree of food processing affects satiety potential and glycemic impact (2) Glycemic glucose equivalent (GGE) may better reflect food processing than glycemic index (GI) - Recommended research direction The relationship between food processing, satiety, and glycemic potential needs further study |
| Gallegos-Riofrío et al., 2021^30^ | - Caliata provides important perspectives on linkages between diet, biodiversity, use of agroecological space, and rural-urban dynamics. - Caliata's residents express a strong preference for a healthy diet composed of unprocessed foods while the community showed a low prevalence of chronic disease and high fruit and vegetable consumption, thus Caliata may illustrate positive deviance in the context of food systems | - NOVA alignment with other SDI(s): Caliata provides important perspectives on linkages between diet, biodiversity, use of agroecological space, and rural-urban dynamics |
| García et al., 2023^31^ | - Lower ultra-processed food (UPF) consumption can reduce greenhouse gas emissions and energy use while increasing water use - Adherence to the Mediterranean diet can help reduce UPF consumption due to its focus on fresh, minimally processed foods - Diets based on plant-based food groups, such as the Mediterranean diet, are associated with greater water use due to the water-intensiveness of growing vegetables, fresh fruits, and nuts - Land use was not found to be significant in the study, with relatively low contributions from meat-free UPF products in terms of land use - Higher percentage of UPF consumption would suppose an increase of GHGs and use of energy and a decrease of water use in both years. | - Sustainable dietary implications related to NOVA classification: (1) Lower ultra-processed food (UPF) consumption can reduce greenhouse gas emissions and energy use while increasing water use (2) Adherence to the Mediterranean diet can help reduce UPF consumption due to its focus on fresh, minimally processed foods - NOVA alignment with other SDI(s): Land use was not found to be significant in the study, with relatively low contributions from meat-free UPF products in terms of land use. Higher percentage of UPF consumption would suppose an increase of GHGs and use of energy and a decrease of water use in both years. |
| Garzillo et al., 2022^32^ | - Based on the analysis of data collected by two 24-hour food records in a probabilistic sample of the Brazilian population aged 10 years and over (n = 32,886), we found a significant linear association between the dietary contribution of the ultra-processed food group and the diet water footprint, even after adjusting for potential sociodemographic confounders. As far as we know, this is the first nationally representative study to show a negative environmental effect of consuming ultra-processed foods. The same linear association shown for the diet carbon footprint in the crude analysis was no longer significant after adjusting for the same sociodemographic variables. - Our results also showed that the additional adjustment for energy intake eliminated the association between the dietary contribution of ultra-processed foods and the diet water footprint, indicating the mediating role of total energy intake in that association. | - NOVA alignment with other SDI(s): (1) We found a significant linear association between the dietary contribution of the ultra-processed food group and the diet water footprint, even after adjusting for potential sociodemographic confounders. The same linear association shown for the diet carbon footprint in the crude analysis was no longer significant after adjusting for the same sociodemographic variables. (2) Our results also showed that the additional adjustment for energy intake eliminated the association between the dietary contribution of ultra-processed foods and the diet water footprint, indicating the mediating role of total energy intake in that association. |
| Grech et al., 2022^33^ | - NOVA and ADG systems show considerable overlap in classification - Some foods classified as unhealthy in ADG are not in NOVA - - NOVA and ADG systems differ in their impact on energy intake; the NOVA system includes relatively protein-dense foods as ultra-processed | - NOVA alignment with other SDI(s): NOVA and ADG systems show considerable overlap in classification |
| Gupta et al., 2019^34^ | - Ultra-processed foods tend to be energy-dense, low-cost, and nutrient-poor. Low energy costs could be one mechanism linking ultra-processed foods with negative health outcomes. Food-based Dietary Guidelines may need to address food processing about economic aspects of food choice - The NOVA categories were characterized by sharply different food costs. Ultra-processed foods had lower NRF9.3 scores than unprocessed foods but were also much less expensive. | - Policy implications: Ultra-processed foods tend to be energy-dense, low-cost, and nutrient-poor. Low energy costs could be one mechanism linking ultra-processed foods with negative health outcomes. Food-based Dietary Guidelines may need to address food processing about economic aspects of food choice - NOVA alignment with other SDI(s): The NOVA categories were characterized by sharply different food costs. Ultra-processed foods had lower NRF9.3 scores than unprocessed foods but were also much less expensive. |
| Gupta et al., 2021^35^ | - Low-cost foods of high energy density and low nutritional value that are selected by lower-income groups have long been associated with adverse health outcomes. - Studies on socio-economic determinants of health would do well to take food prices and affordability into account. - Efforts to reduce UP foods consumption, an increasingly common policy measure, need to take affordability, food expenditures and diet costs into account. | - Policy implications: Efforts to reduce UP foods consumption, an increasingly common policy measure, need to take affordability, food expenditures and diet costs into account |
| Hallinan et al., 2021^36^ | - First, we demonstrated that a combination of unprocessed and ultra-processed foods was required for the model to generate a feasible food pattern. Notably, models consisting of just unprocessed foods or ultra-processed foods did not have any solutions. - The present analyses clearly show that foods that fall into the NOVA ultra-processed category were the principal sources of added sugar, sodium, and saturated fat. On the other hand, those same foods were also among the major contributors of vitamin E, thiamin, niacin, folate, and calcium, and were the main sources of plant protein | - Sustainable dietary implications related to NOVA classification: we demonstrated that a combination of unprocessed and ultra-processed foods was required for the model to generate a feasible food pattern. Notably, models consisting of just unprocessed foods or ultra-processed foods did not have any solutions. - NOVA alignment with other SDI(s): NOVA ultra-processed category were the principal sources of added sugar, sodium, and saturated fat. On the other hand, those same foods were also among the major contributors of vitamin E, thiamin, niacin, folate, and calcium, and were the main sources of plant protein |
| Hässig et al., 2023^37^ | - NOVA classification system reflects laypeople's perceptions - Consumers have negative associations with food processing - Perceived degree of processing influences the evaluation of food healthiness - Strong agreement between laypeople's perceptions and NOVA classification | - NOVA alignment with other SDI(s): (1)NOVA classification system reflects laypeople's perceptions (2) Consumers have negative associations with food processing (3)Perceived degree of processing influences the evaluation of food healthiness (4) Strong agreement between laypeople's perceptions and NOVA classification |
| Julia et al., 2023^38^ | - Nutritional quality and ultra-processing are correlated but distinct dimensions of the diet - Both dimensions contribute substantially to the healthiness of the diet, it highlights the importance of considering both dimensions in diet analysis - Cross-effects between nutritional quality and ultra-processed foods have synergistic effects | - NOVA alignment with other SDI(s): (1) Nutritional quality and ultra-processing are correlated but distinct dimensions of the diet (2) Cross-effects between nutritional quality and ultra-processed foods have synergistic effects - Recommendation to use NOVA with other SDI(s): Both dimensions contribute substantially to the healthiness of the diet, it highlights the importance of considering both dimensions in diet analysis |
| Juul et al., 2019^39^ | - Higher purchases of ultra-processed foods linked to lower overall diet quality - At similar levels of ultra-processed food purchases, households purchasing more minimally processed fruits, vegetables, roots and legumes had higher diet quality than households purchasing less of these foods - Saturated fat component scores did not differ based on the processing level of food purchases - Households purchasing the most ultra-processed foods achieved the highest component scores for whole grains - Increasing the proportion of minimally processed plant foods and reducing ultra-processed foods linked to better diet quality | - NOVA alignment with other SDI(s): (1) Higher purchases of ultra-processed foods linked to lower overall diet quality (2) At similar levels of ultra-processed food purchases, households purchasing more minimally processed fruits, vegetables, roots and legumes had higher diet quality than households purchasing less of these foods (3) Saturated fat component scores did not differ based on the processing level of food purchases (4) Households purchasing the most ultra-processed foods achieved the highest component scores for whole grains - Sustainable dietary implications related to NOVA classification: Increasing the proportion of minimally processed plant foods and reducing ultra-processed foods linked to better diet quality |
| Juul et al., 2021^40^ | - In the Framingham Offspring Cohort (FOS), the declining intake of ultra-processed foods occurred in parallel to modest improvements in diet quality and reductions in the intakes of trans fats and Na, as well as sucrose and fructose, the predominant added sugars in the US food supply - Our findings align with previous observations in NHANES that the quality of the US diet improved from 1999 to 2011 - Our findings contribute to the existing evidence suggesting that limiting ultra-processed foods may be an effective strategy to improve diet quality | - Sustainable dietary implications related to NOVA classification: (1) In the Framingham Offspring Cohort (FOS), the declining intake of ultra-processed foods occurred in parallel to modest improvements in diet quality and reductions in the intakes of trans fats and Na, as well as sucrose and fructose, the predominant added sugars in the US food supply (2) Our findings contribute to the existing evidence suggesting that limiting ultra-processed foods may be an effective strategy to improve diet quality |
| Kesse-Guyot et al., 2023^41^ | - After we adjusted for calories consumed, the associations with greenhouse gas emissions and land use vanished, and the associations with water use and energy demand became negative. However, the processing and packaging stages contributed significantly to energy demand. Post-farm stages, such as final-product creation and packaging, contributed to greater environmental impacts of UPF-rich diets. - In the present study conducted on a representative survey of the French population, we observed that participants with a higher percentage of UPF in their diets had a higher energy intake, which explained their higher environmental pressure for most of the studied indicators. - Most pressures occurred at the stage of agricultural production, apart from energy demand, for which the processing and packaging stages were also important contributors. NOVA1 (unprocessed or minimally processed) food consumption highly contributed to land use and GHGe in all quintiles. NOVA4 (UPF) food consumption greatly contributed to energy demand. - In addition to potential human health benefits, the reduction of high UPF consumption (associated with a greater overall energy intake) could be a driver in the transition towards a more sustainable food system by contributing to the reduction of GHGe, energy demand, land use, soil and water degradation, and pollution. Reduced UPF consumption is thus an important factor to consider in the fight against the global syndemic of obesity, undernutrition, and climate change | - Sustainable dietary implications related to NOVA classification: (1) In the present study conducted on a representative survey of the French population, we observed that participants with a higher percentage of UPF in their diets had a higher energy intake, which explained their higher environmental pressure for most of the studied indicators. (2) In addition to potential human health benefits, the reduction of high UPF consumption (associated with a greater overall energy intake) could be a driver in the transition towards a more sustainable food system by contributing to the reduction of GHGe, energy demand, land use, soil and water degradation, and pollution. - NOVA alignment with other SDI(s): (1) most pressures occurred at the stage of agricultural production, apart from energy demand, for which the processing and packaging stages were also important contributors. NOVA1 (unprocessed or minimally processed) food consumption highly contributed to land use and GHGe in all quintiles. NOVA4 (UPF) food consumption greatly contributed to energy demand. (2) After we adjusted for calories consumed, the associations with greenhouse gas emissions and land use vanished, and the associations with water use and energy demand became negative. However, the processing and packaging stages contributed significantly to energy demand. Post-farm stages, such as final-product creation and packaging, contributed to greater environmental impacts of UPF-rich diets - Policy implications: Reduced UPF consumption is thus an important factor to consider in the fight against the global syndemic of obesity, undernutrition, and climate change |
| Lavigne-Robichaud et al., 2018^42^ | - Although diet quality indices have been associated with cardiometabolic risk, only the dietary intake of UPP was significantly associated with MetS in the Eeyouch. - Diet quality scores (aHEI-2010, FQS) were very low and the contribution of UPP to energy intake was relatively high at 52 %. When assessing the aHEI-2010, FQS and contribution of UPP to total daily dietary energy intake with risk of MetS, the contribution of UPP showed the strongest association. However, no significant association was observed with the diet quality scores provided by aHEI-2010 and FQS | - NOVA alignment with other SDI(s): (1) Although diet quality indices have been associated with cardiometabolic risk, only the dietary intake of UPP was significantly associated with MetS in the Eeyouch. (2) Diet quality scores (aHEI-2010, FQS) were very low and the contribution of UPP to energy intake was relatively high at 52 %. When assessing the aHEI-2010, FQS and contribution of UPP to total daily dietary energy intake with risk of MetS, the contribution of UPP showed the strongest association. However, no significant association was observed with the diet quality scores provided by aHEI-2010 and FQS |
| Liu et al., 2022^43^ | - - Higher UPF consumption linked to poor diet quality in children and adults - - As UPF calories increase, consumption of healthy food decreases, and unhealthy food increases | - NOVA alignment with other SDI(s): Higher UPF consumption linked to poor diet quality in children and adults |
| Maia et al., 2022^44^ | - Increased consumption of unprocessed or minimally processed foods (except for animal-based foods) leads to lower diet cost and better nutritional profile while the reduction of ultra-processed foods also reduces diet cost and improves nutritional quality - Eating unprocessed foods improves the nutritional composition of the diet - This study shows a correlation between food processing and diet cost/nutritional composition | - Sustainable dietary implications related to NOVA classification: Increased consumption of unprocessed or minimally processed foods (except for animal-based foods) leads to lower diet cost and better nutritional profile while the reduction of ultra-processed foods also reduces diet cost and improves nutritional quality - NOVA alignment with other SDI(s): Increased consumption of unprocessed or minimally processed foods (except for animal-based foods) leads to lower diet cost and better nutritional profile while the reduction of ultra-processed foods also reduces diet cost and improves nutritional quality |
| Marchese et al., 2022^45^ | - Research highlights dietary inequalities in Australia and the negative effects of Ultra-Processed Foods (UPF) on diet quality - UPF consumption negatively impacts diet quality and various aspects of a healthy diet, such as diverse nutritious foods, fruits, vegetables, grains, lean protein, and hydration. | - Policy implications: Research highlights dietary inequalities in Australia and the negative effects of Ultra-Processed Foods (UPF) on diet quality. - Sustainable dietary implications related to NOVA classification: UPF consumption negatively impacts diet quality and various aspects of a healthy diet, such as diverse nutritious foods, fruits, vegetables, grains, lean protein, and hydration. |
| Martinez-Perez & Arroyo-Izaga, 2021^46^ | - It should be noted that the processing level classiﬁcation used in the present study showed a low level of agreement with the NPMs. This result is probably due to the fact that although ultra-processed foods usually are characterized by a high content of sugar, salt, and/or fats, these contents do not always exceed the limits of the NPMs. | - NOVA alignment with other SDI(s): It should be noted that the processing level classiﬁcation used in the present study showed a low level of agreement with the NPMs. This result is probably due to the fact that although ultra-processed foods usually are characterized by a high content of sugar, salt, and/or fats, these contents do not always exceed the limits of the NPMs. |
| Martinez-Perez et al., 2021^47^ | - UPF consumption quintiles by different DP classification systems showed marked differences in subject agreement - High UPF consumption is associated with poor-quality diet and markers of cardiometabolic risk. - Different food processing-based classification systems show variations in associations between UPF consumption and cardiometabolic markers. | - NOVA alignment with other SDI(s) : (1) High UPF consumption is associated with poor-quality diet and markers of cardiometabolic risk (2) Different food processing-based classification systems show variations in associations between UPF consumption and cardiometabolic markers |
| Martinez‑Perez et al., 2022^48^ | - Notably, the (sQ-HPF ) questionnaire was designed to encompass criteria from four leading food processing-based classifcation systems – NOVA, IARC, IFIC, and UNC –, so the categorization as HPF consumer occurs according to at least two of these classifcation systems (tertile agreement). This is a key asset of the sQ-HPF, which makes it more comprehensive than other methods that have been proposed and are limited to one classifcation system. In this regard, the limitations of the NOVA system, the most used classifcation system, have been widely discussed and it has been shown that the choice of the classifcation system can have a signifcant impact on research - outcomesThe sQ-HPF is an integrative, short, and easy-to-use questionnaire that can be used to screen HPF consumption in large populations for epidemiological or public health purposes. | - Recommendation for the application of NOVA: The limitations of the NOVA system, the most used classification system, have been widely discussed and it has been shown that the choice of the classification system can have a significant impact on research outcomes. |
| Mendes et al., 2021^49^ | - Food marketing carried out in this media can encourage unhealthy diets, due to the higher incidence of advertising processed and ultra-processed foods with critical nutrients in excess, although no greater financial stimulus was noted for these products. - The present study indicated that the cost of processed foods was significantly higher than the other categories and, that there are no differences between the price of fresh or minimally processed foods and the ultra-processed foods advertised. | - Policy implications: Food marketing carried out in this media can encourage unhealthy diets, due to the higher incidence of advertising processed and ultra-processed foods with critical nutrients in excess, although no greater financial stimulus was noted for these products. - NOVA alignment with other SDI(s): The present study indicated that the cost of processed foods was significantly higher than the other categories and, that there are no differences between the price of fresh or minimally processed foods and the ultra-processed foods advertised. |
| Mendoza-Velázquez et al., 2022^50^ | - Affordable nutrient-rich foods are found in different NOVA categories, thus, nutritionally adequate diets can include foods from all NOVA categories - MPF have higher nutrient density but cost more per calorie, while UPF cost less per calorie but are lower in nutritional value - Low- and middle-income countries often lack priority nutrients | - NOVA alignment with other SDI(s): (1) Affordable nutrient-rich foods are found in different NOVA categories, thus, nutritionally adequate diets can include foods from all NOVA categories (2) MPF have higher nutrient density but cost more per calorie, while UPF cost less per calorie but are lower in nutritional value - Policy implications: Low- and middle-income countries often lack priority nutrients |
| Mignogna et al., 2022^51^ | - Incremental association between food processing, as reﬂected by the NOVA classiﬁcation, and low-grade inﬂammation, | - NOVA alignment with other SDI(s): Incremental association between food processing, as reﬂected by the NOVA classiﬁcation, and low-grade inﬂammation |
| Morales et al., 2020^52^ | - No significant differences in acrylamide and HMF content between processed and ultra-processed breakfast cereals (NOVA-3 and NOVA-4), however, significant differences between un-/minimally processed (NOVA-1) and ultra-processed breakfast cereals (NOVA-4). - Ingredient composition complexity is the only factor differentiating processed and ultra-processed breakfast cereals in terms of heat-induced chemical markers of processing. - Higher acrylamide content in ultra-processed breakfast cereals, with samples exceeding benchmark level all being ultra-processed. - Results suggest a need for reformulation of ultra-processed breakfast cereals. | - NOVA alignment with other SDI(s): No significant differences in acrylamide and HMF content between processed and ultra-processed breakfast cereals (NOVA-3 and NOVA-4), however, significant differences between un-/minimally processed (NOVA-1) and ultra-processed breakfast cereals (NOVA-4). - Policy implications: Results suggest a need for reformulation of ultra-processed breakfast cereals. |
| Otten et al., 2017^53^ | - We ﬁnd no evidence of changes in food prices overall or by food group, level of food processing, or nutrient density score, attributable to two years of exposure to the Seattle minimum wage ordinance and an increase in hourly wage to $13.50–$15.00 per hour. | - Policy implications: We ﬁnd no evidence of changes in food prices overall or by food group, level of food processing, or nutrient density score, attributable to two years of exposure to the Seattle minimum wage ordinance and an increase in hourly wage to $13.50–$15.00 per hour. |
| Phulkerd et al., 2023^54^ | - - NOVA accounts for the interaction of biochemically active components and the food matrix. This challenges the dominant nutrient paradigm in nutrition since it contends that a food's 'healthfulness' is more than the sum of its nutrients - - A hybrid food classification approach taking both nutrients and food processing into account is needed to comprehensively assess the nutritional quality of food and beverage products in Thailand. | - Supporting argument on NOVA: NOVA accounts for the interaction of biochemically active components and the food matrix. This challenges the dominant nutrient paradigm in nutrition since it contends that a food's 'healthfulness' is more than the sum of its nutrients - Recommendation to use NOVA with other SDI(s):a hybrid food classification approach taking both nutrients and food processing into account is needed to comprehensively assess the nutritional quality |
| Phulkerd et al., 2023^55^ | - A system for regularly monitoring and evaluating the healthiness (both nutritional and processing aspects) of food products, especially UPFs, is required. The combined use of the NOVA classification scheme and the WHO SEA Nutrient Profile Model allowed for a more holistic assessment - The findings suggest a need for regulatory and non-regulatory measures such as UPF taxation and marketing restrictions, and market incentives for producing non-UPFs. A system for regularly monitoring and evaluating healthiness (both nutritional and processing aspects) of food products, especially UPFs, is required. | - Policy implications: (1) A system for regularly monitoring and evaluating the healthiness (both nutritional and processing aspects) of food products, especially UPFs, is required. The combined use of the NOVA classification scheme and the WHO SEA Nutrient Profile Model allowed for a more holistic assessment (2) The findings suggest a need for regulatory and non-regulatory measures such as UPF taxation and marketing restrictions, and market incentives for producing non-UPFs. A system for regularly monitoring and evaluating healthiness (both nutritional and processing aspects) of food products, especially UPFs, is required. |
| Pulker et al., 2018^56^ | - The HSR scores for nutrient-poor discretionary foods and UPF show inconsistencies; The mean HSR scores for nutrient-poor discretionary foods, mixed products high in fat sugar or salt, and UPF, and the predominance of UPF with HSR scores indicating they were nutritious choices, further illuminate the inconsistencies between HSR and other measures of nutritional quality. - The HSR algorithm fails to score recommended dairy foods, discretionary foods, and UPF appropriately - HSR on SOBF promotes nutrient-poor food choices - Many nutrient-poor foods achieved HSR scores inaccurately indicating they were healthy choices | - NOVA alignment with other SDI(s): The HSR scores for nutrient-poor discretionary foods and UPF show inconsistencies; The mean HSR scores for nutrient-poor discretionary foods, mixed products high in fat sugar or salt, and UPF, and the predominance of UPF with HSR scores indicating they were nutritious choices, further illuminate the inconsistencies between HSR and other measures of nutritional quality. |
| Rizzolo-Brime et al., 2023^57^ | - This study shows that a great number of plant-based meat alternative products available in Spanish supermarkets have a variable nutritional composition depending on the product category. There exists a false belief about the healthiness of these products because of their plant origin. - Although PBMAs may show medium to high contents of vegetable protein and can be considered sources of dietary fiber, the majority of these products also meet the criteria of ultra-processed food, so their consumption should be sporadic within a plant-based diet with fresh vegetables, fruit, and legumes. - After a closer examination, both nutritional and quality information about PBMAs is needed to develop guidelines to counsel consumers about including these products in a healthy plant-based diet. | - Sustainable dietary implications related to NOVA classification: (1) This study shows that a great number of plant-based meat alternative products available in Spanish supermarkets have a variable nutritional composition depending on the product category. There exists a false belief about the healthiness of these products because of their plant origin. (2) Although PBMAs may show medium to high contents of vegetable protein and can be considered sources of dietary fiber, the majority of these products also meet the criteria of ultra-processed food, so their consumption should be sporadic within a plant-based diet with fresh vegetables, fruit, and legumes - Policy implications: After a closer examination, both nutritional and quality information about PBMAs is needed to develop guidelines to counsel consumers about including these products in a healthy plant-based diet |
| Robert et al., 2022^58^ | - More resilient individuals tended to have a better overall diet quality (mPNNS-GS), reflected by a lower consumption of UPFs, dairy desserts, sugary fatty products, and sugar and confectionery, and a higher consumption of seafood, whole-grain foods, and unsalted oleaginous fruits. | - NOVA alignment with other SDI(s): More resilient individuals tended to have a better overall diet quality (mPNNS-GS), reflected by a lower consumption of UPFs, dairy desserts, sugary fatty products, and sugar and confectionery, and a higher consumption of seafood, whole-grain foods, and unsalted oleaginous fruits. |
| Rodrigues et al., 2016^59^ | - The NOVA system is stricter than the UK/Ofcom model in classifying foods targeted at children as 'less healthy' - Foods marketed at children that bore nutrient claims were signiﬁcantly ‘less healthy’ compared with foods that did not bore nutrient claims according to the NOVA model. - The high number of foods with low nutritional quality being marketed to children via product packaging and nutrient claims should be of concern to policymakers wanting to improve children's diets and tackle childhood obesity. | - NOVA alignment with other SDI(s): the NOVA system is stricter than the UK/Ofcom model in classifying foods targeted at children as 'less healthy' - Policy implications: The high number of foods with low nutritional quality being marketed to children via product packaging and nutrient claims should be of concern to policymakers wanting to improve children's diets and tackle childhood obesity. |
| Romero Ferreiro et al., 2021^60^ | - Nutri-Score should be accompanied by complementary labeling indicating the level of processing, such as the NOVA classification - All Nutri-Score categories include ultra-processed foods, with a high proportion in categories B, C, D, and E - The weight of processing seems to be even greater than the nutrient content in influencing health - Eating complex food matrices has a greater physiological effect than enteral and parenteral nutrient solutions - Nutri-Score alone may not fully account for the impact of food processing on health | - Policy implications: Nutri-Score should be accompanied by complementary labeling indicating the level of processing, such as the NOVA classification |
| Rossato et al., 2023^61^ | - The intake of MP was directly and UPF inversely associated with the AHEI-2010, aMED, and DASH-diet scores in these two prospective cohorts of older adults. Such associations were not completely consistent over time, which could be partially attributed to the consumption of processed foods and processed culinary ingredients food-processing categories or changes in the FFQ. | - NOVA alignment with other SDI(s): The intake of MP was directly and UPF inversely associated with the AHEI-2010, aMED, and DASH-diet scores in these two prospective cohorts of older adults. |
| Ruggiero et al., 2021^62^ | - Increased adherence to the Mediterranean diet would possibly result in lower UPF consumption. - As already documented in other populations, higher intake of UPF was associated with lower diet quality, being richer in Na, fat and poor in fiber, and inversely with the Mediterranean diet, both in adults and children/adolescents - Since the traditional Mediterranean diet features unprocessed or minimally processed food and emphasizes home cooking, its inverse association with UPF possibly accounts for the relatively lower energy from UPF in our participants as compared with much higher estimations reported in other countries, especially non-Mediterranean. | - Sustainable dietary implications related to NOVA classification: (1) Increased adherence to the Mediterranean diet would possibly result in lower UPF consumption. (2) Since the traditional Mediterranean diet features unprocessed or minimally processed food and emphasizes home cooking, its inverse association with UPF possibly accounts for the relatively lower energy from UPF in our participants as compared with much higher estimations reported in other countries, especially non-Mediterranean - NOVA alignment with other SDI(s): As already documented in other populations, higher intake of UPF was associated with lower diet quality, being richer in Na, fat and poor in fiber, and inversely with the Mediterranean diet, both in adults and children/adolescents |
| Salomé et al., 2021^63^ | - We also show that protein consumption patterns differed according to the degree of food processing and that it explains part of the associations with diet quality. Higher consumers of MPF had a higher protein intake, with a lower plant:animal protein ratio, but they had a healthier plant-based protein intake and a fully favorable dietary pattern. Higher consumers of PF had higher plant protein intakes but lower plant protein diversity and a greater risk of cardiometabolic death. - We can thus conclude that healthy plant protein patterns are crucial to a future shift in protein intake in the context of a food system that is increasingly dominated by ultra-processed foods. - In the French population, in contrast with UPFp, higher MPFp was associated with higher animal protein intake, better plant protein diversity, higher diet quality and markedly lower cardiometabolic risk. | - NOVA alignment with other SDI(s): We also show that protein consumption patterns differed according to the degree of food processing and that it explains part of the associations with diet quality. Higher consumers of MPF had a higher protein intake, with a lower plant:animal protein ratio, but they had a healthier plant-based protein intake and a fully favorable dietary pattern. Higher consumers of PF had higher plant protein intakes but lower plant protein diversity and a greater risk of cardiometabolic death. - Sustainable dietary implications related to NOVA classification: We can thus conclude that healthy plant protein patterns are crucial to a future shift in protein intake in the context of a food system that is increasingly dominated by ultra-processed foods. |
| Shim et al., 2020^64^ | - Ultra-processed food consumption by Korean adults constituted one-fourth of the daily energy intake, and a higher energy contribution of ultra-processed foods was associated with poorer dietary intakes and a lower dietary quality | - NOVA alignment with other SDI(s): higher energy contribution of ultra-processed foods was associated with poorer dietary intakes and a lower dietary quality |
| Siqueira et al., 2021^65^ | - Some of the consumed foods may be processed or ‘ultra-processed’, but low-cost ingredients can also be purchased and prepared, and consumed at home. The present data suggest that the cheapest foods in Brazil were energy-dense and minimally processed. - Most of the FBDG's advice had to do with selecting minimally processed v. ultra-processed foods and limiting the consumption of added fat, sugar, and salt. The underlying assumption was that minimally processed foods were still relatively inexpensive as compared with other options. In the present study, energy density was defined as kJ/100 g, whereas nutrient density was measured using a Nutrient Rich Food 8.2 nutrient density score, effectively a nutrient-to-calorie ratio. Affordability was calculated as calories or nutrients per reference amount. Data on the affordability of energy-dense v. nutrient-rich foods in low- and middle-income countries are relatively limited. | - Recommended research direction: Data on the affordability of energy-dense v. nutrient-rich foods in low- and middle-income countries are relatively limited. - Sustainable dietary implications related to NOVA classification: Some of the consumed foods may be processed or ‘ultra-processed’, but low-cost ingredients can also be purchased and prepared, and consumed at home. The present data suggest that the cheapest foods in Brazil were energy-dense and minimally processed. |
| Sneed et al., 2023^66^ | - It is recommended that future researchers use the double-coding methodology and expert adjudication for Nova categorization to resolve discordant food items' classification - Nova categorization aligns with macronutrient and HEI criteria, showing its validity - UPF intake assessment should not be considered interchangeable with macronutrient and HEI criteria assessment | - Recommendation for the application of NOVA: It is recommended that future researchers use the double-coding methodology and expert adjudication for Nova categorization - NOVA alignment with other SDI(s): Nova categorization aligns with macronutrient and HEI criteria - Recommendation to use NOVA with other SDI(s): UPF intake assessment should not be considered interchangeable with macronutrient and HEI criteria assessment |
| Spiteri et al., 2018^67^ | - According to nutrient-, food-, and processing-based criteria, the majority of these new products were classified in each scheme's least healthy category (i.e. red, discretionary, and ultra-processed). - These findings suggest that manufacturers have little interest in launching new fruit and vegetable products, particularly minimally processed varieties. | - NOVA alignment with other SDI(s): According to nutrient-, food-, and processing-based criteria, the majority of these new products were classified in each scheme's least healthy category (i.e. red, discretionary, and ultra-processed). - Policy implications: These findings suggest that manufacturers have little interest in launching new fruit and vegetable products, particularly minimally processed varieties. |
| Trübwasser et al., 2022^68^ | - For both groups of students, our study found a high density of food outlets within the 0.5 km radius around the schools, as well as widespread promotion and display of ultra-processed foods and beverages in and around the schools. | - Policy implications: For both groups of students, our study found a high density of food outlets within the 0.5 km radius around the schools, as well as widespread promotion and display of ultra-processed foods and beverages in and around the schools |
| Valenzuela et al., 2022^69^ | - there are quantitative and qualitative differences between the classification and recommendation guidelines of the Nutri-Score, NOVA, and FoP warning labels, finding concrete discrepancies between them. - Nutri-Score and FoP warning labels show similarity in classification guidelines - NOVA system differs in classification guidelines compared to the other two that show greater homogeneity in classification - Products with positive characteristics may not fall into NOVA 1, 2, or 3 categories | - NOVA alignment with other SDI(s): there are quantitative and qualitative differences between the classification and recommendation guidelines of the Nutri-Score, NOVA, and FoP warning labels, finding concrete discrepancies between them. |
| van Dam & Vandevijvere, 2022^70^ | - Only a limited proportion of the promotions in supermarket flyers was for fresh fruits and vegetables while more than half of the promotions were for ultra-processed foods. - Performance metrics relating to food formulation and marketing were not associated with the overall BIA-Obesity score on commitments. | - Policy implications: (1) Only a limited proportion of the promotions in supermarket flyers was for fresh fruits and vegetables while more than half of the promotions were for ultra-processed foods. (2) Performance metrics relating to food formulation and marketing were not associated with the overall BIA-Obesity score on commitments. |
| van Dam, Reimes & Vandevijvere, 2022^71^ | - The performance indicators indicated unhealthy food environments with the majority of portfolios consisting of ultra-processed foods and products not permitted to be marketed to children, only limited promotion of fresh fruits and vegetables in supermarket fyers, and several quick-service restaurants having most of their outlets within 500 m road distance of schools. | - Policy implications: The performance indicators indicated unhealthy food environments with the majority of portfolios consisting of ultra-processed foods and products not permitted to be marketed to children, only limited promotion of fresh fruits and vegetables in supermarket fyers, and several quick-service restaurants having most of their outlets within 500 m road distance of schools. |
| Vandevijvere et al., 2020^72^ | - Diets with a larger caloric share of UPF were significantly cheaper than those with a lower contribution of these products, while the opposite was found for MPF. Policies that improve the relative affordability and accessibility of MPF are recommended | - NOVA alignment with other SDI(s): Diets with a larger caloric share of UPF were significantly cheaper than those with a lower contribution of these products, while the opposite was found for MPF. - Policy implications: Policies that improve the relative affordability and accessibility of MPF are recommended. |
| Vellinga et al., 2022^73^ | - Ultra-processed foods and drinks (UPF and UPD) are less healthy due to high energy, SFA, sugar, and sodium content - UPF have higher greenhouse gas emissions but less blue water use and lower food costs compared to MPF, while UDP have similar GHGE emissions, higher blue water use, and higher cost compared to MPD - Replacing UPF with unprocessed or minimally processed foods may increase daily blue water use and food costs - Food costs according to NOVA were not in line with outcomes for nutritional quality, as healthier foods (MPF) were more expensive than UPF, while UPD were twice as expensive as MPD. - NOVA classification is not directly applicable for identifying win-win-wins of nutritional quality, environmental impact, and costs of diets | - NOVA alignment with other SDI(s): (1) ultra-processed foods and drinks (UPF and UPD) are less healthy due to high energy, SFA, sugar, and sodium content (2) UPF have higher greenhouse gas emissions but less blue water use and lower food costs compared to MPF, while UDP have similar GHGE emissions, higher blue water use, and higher cost compared to MPD (3) Food costs according to NOVA were not in line with outcomes for nutritional quality, as healthier foods (MPF) were more expensive than UPF, while UPD were twice as expensive as MPD - Critics on NOVA: NOVA classification is not directly applicable for identifying win-win-wins of nutritional quality, environmental impact, and costs of diets |
| Vellinga et al., 2023^74^ | - Higher consumption of Ultra-Processed Foods (UPFs) is linked to lower environmental impact, while higher consumption of Ultra-Processed Drinks (UPDs) is linked to adverse health effects and higher environmental impact - Reducing UPD consumption can have positive effects on human and planetary health, however, replacing UPFs with less processed alternatives may have varying impacts on health and the environment - Further research is needed to quantify the impact of UPF and UPD consumption on the environment | - NOVA alignment with other SDI(s): Higher consumption of Ultra-Processed Foods (UPFs) is linked to lower environmental impact, while higher consumption of Ultra-Processed Drinks (UPDs) is linked to adverse health effects and higher environmental impact - Sustainable dietary implications related to NOVA classification: Reducing UPD consumption can have positive effects on human and planetary health, however, replacing UPFs with less processed alternatives may have varying impacts on health and the environment - Recommended research direction: Further research is needed to quantify the impact of UPF and UPD consumption on the environment |
| Vergeer et al., 2019^75^ | - These findings demonstrate that calorie- and nutrient-dense foods exist across different levels of processing, suggesting that food choices and dietary recommendations should be based primarily on energy or nutrient density rather than processing classification. - Among the total sample, unprocessed or minimally processed foods contained comparable or more favorable average amounts of sodium, saturated fat, total and free sugars, fiber, and protein per 100 g (or 100 mL) than products in all other processing categories. - Despite their similarities, there are also important differences between the NOVA and Poti et al. food processing classification systems that may help to explain some of the observed trends in nutritional composition between processing levels. The main differences arise in how the two systems treat added sugars, salt, or fat, and how they distinguish between whole- and refined-grains. For example, according to NOVA, flavored milk, yogurts, unsweetened breakfast cereals, and packaged wholegrain bread are considered ultra-processed, whereas, under the Poti et al. system, these products are deemed moderately processed This was reflected in our observation that, among cereals and grain products, median fiber content was higher for ultra-processed foods than other NOVA categories. NOVA has been criticized for its lack of consideration regarding the processing methods or technology used, focusing instead on the addition of sugar, salt, or additives | - Recommendation to use NOVA with other SDI(s): These findings demonstrate that calorie- and nutrient-dense foods exist across different levels of processing, suggesting that food choices and dietary recommendations should be based primarily on energy or nutrient density rather than processing classification. - NOVA alignment with other SDI(s): Among the total sample, unprocessed or minimally processed foods contained comparable or more favorable average amounts of sodium, saturated fat, total and free sugars, fiber, and protein per 100 g (or 100 mL) than products in all other processing categories.(2) Despite their similarities, there are also important differences between the NOVA and Poti et al. food processing classification systems that may help to explain some of the observed trends in nutritional composition between processing levels. The main differences arise in how the two systems treat added sugars, salt, or fat, and how they distinguish between whole- and refined-grains. For example, according to NOVA, flavored milk, yogurts, unsweetened breakfast cereals, and packaged wholegrain bread are considered ultra-processed, whereas, under the Poti et al. system, these products are deemed moderately processed This was reflected in our observation that, among cereals and grain products, median fiber content was higher for ultra-processed foods than other NOVA categories - Critics on NOVA: NOVA has been criticized for its lack of consideration regarding the processing methods or technology used, focusing instead on the addition of sugar, salt, or additives |
| Vicente et al., 2023^76^ | - The inclusion of UPR in the multiple models did not significantly change the R2 values, therefore, not confirming our hypothesis of using UPR as a complementary index to be associated with our plasma inflammatory indicator. | - NOVA alignment with other SDI(s): The inclusion of UPR in the multiple models did not significantly change the R2 values, therefore, not confirming our hypothesis of using UPR as a complementary index to be associated with our plasma inflammatory indicator. |
| Vogliano et al., 2021^77^ | - Urban populations were found to have a reduced ability to self-cultivate agri-food products or collect wild foods, and therefore consumed more ultra-processed foods (classified as NOVA 4) and takeout foods, and overall had less diverse diets compared to rural populations - Food fortification may solve single micronutrient deficiencies but could ultimately reduce the sustainability of indigenous food system and give rise to diet-related noncommunicable diseases (NCDs), particularly since most fortified foods are imported and tend to be processed. | - Policy implications: (1) Urban populations were found to have a reduced ability to self-cultivate agri-food products or collect wild foods, and therefore consumed more ultra-processed foods (classified as NOVA 4) and takeout foods, and overall had less diverse diets compared to rural populations (2) Food fortification may solve single micronutrient deficiencies but could ultimately reduce the sustainability of indigenous food system and give rise to diet-related noncommunicable diseases (NCDs), particularly since most fortified foods are imported and tend to be processed. |

References:

S1 Abreu S, Liz Martins M. Cross-Classification Analysis of Food Products Based on Nutritional Quality and Degree of Processing. Nutrients. 2023;15(14):3117. doi:10.3390/nu15143117.

S2 Aceves-Martins M, Bates RL, Craig LC, et al. Nutritional quality, environmental impact and cost of ultra-processed foods: a UK food-based analysis. Int. J. Environ. Res. Public Health. 2022;19(6):3191. doi:10.3390/ijerph19063191

S3 Angelino D, Dinu M, Gandossi B, et al. Processing and nutritional quality of breakfast cereals sold in Italy: results from the Food Labelling of Italian Products (FLIP) Study. Nutrients. 2023;15(8):2013. doi:10.3390/nu15082013

S4 Baldridge AS, Huffman MD, Taylor F, et al. The healthfulness of the US packaged food and beverage supply: a cross-sectional study. Nutrients. 2019;11(8):1704. doi:10.3390/nu11081704

S5 Barrett EM, Gaines A, Coyle DH, et al. Comparing product healthiness according to the Health Star Rating and the NOVA classification system and implications for food labelling systems: An analysis of 25 486 products in Australia. Nutr Bull. 2023;48(4):523-534. doi:10.1111/nbu.12640. Epub 2023 Oct 28

S6 Batal M, Johnson-Down L, Moubarac JC, et al. Quantifying associations of the dietary share of ultra-processed foods with overall diet quality in First Nations peoples in the Canadian provinces of British Columbia, Alberta, Manitoba and Ontario. Public Health Nutr. 2018;21(1):103-113. doi:10.1017/S1368980017001677

S7 Batista CHK, Leite FHM, Borges CA. Association between advertising patterns and ultra-processed food in small markets. Ciência & Saúde Coletiva, 2022;27:2667-2678. doi:10.1590/1413-81232022277.19122021.

S8 Baye K, Yaregal Z. The Global Diet Quality Score predicts diet quality of women of reproductive age in Addis Ababa, Ethiopia. BJN. 2023;130:1573-1579. doi:10.1017/S0007114523000508.

S9 Berardy A, Fresán U, Matos RA, et al. Environmental impacts of foods in the Adventist health study-2 dietary questionnaire. Sustainability. doi:2020;12:10267. doi:10.3390/su122410267

S10 Blanchet R, Willows N, Johnson S, et al. Traditional food, health, and diet quality in Syilx Okanagan adults in British Columbia, Canada. Nutrients. 2020;12:927. doi:10.3390/nu12040927

S11 Bleiweiss-Sande R, Chui K, Evans EW, et al. Robustness of Food Processing Classification Systems. Nutrients. 2019;11:1344. doi:10.3390/nu11061344.

S12 Bonaccio M, Di Castelnuovo A, Ruggiero E, et al. Joint association of food nutritional profile by Nutri-Score front-of-pack label and ultra-processed food intake with mortality: Moli-sani prospective cohort study. BMJ. 2022,378. doi:10.1136/bmj-2022-070688

S13 Braesco V, Souchon I, Sauvant P, et al. Ultra-processed foods: how functional is the NOVA system?. Eur. J. Clin. Nutr. 2022;76(9):1245-1253. doi:10.1038/s41430-022-01099-1

S14 Cediel G, Reyes M, Corvalán C, et al. Ultra-processed foods drive to unhealthy diets: evidence from Chile. Public Health Nutr. 2021;24(7):1698-1707. doi:10.1017/S1368980019004737

S15 Chen YC, Huang YC, Lo YTC, et al. Secular trend towards ultra-processed food consumption and expenditure compromises dietary quality among Taiwanese adolescents. Food Nutr Res. 2018,62. doi:10.29219/fnr.v62.1565

S16 Cooper SL, Pelly FE, Lowe JB. Assessment of the construct validity of the Australian Health Star Rating: a nutrient profiling diagnostic accuracy study. Eur. J. Clin. Nutr. 2017;71(11):1353-1359. doi:10.1038/ejcn.2017.23

S17 da Rocha BRS, Rico-Campà A, Romanos-Nanclares A, et al. Adherence to Mediterranean diet is inversely associated with the consumption of ultra-processed foods among Spanish children: The SENDO project. Public Health Nutr. 2021;24:3294-3303. doi:10.1017/S1368980020001524

S18 da Silva JT, Garzillo JMF, Rauber F, et al. Greenhouse gas emissions, water footprint, and ecological footprint of food purchases according to their degree of processing in Brazilian metropolitan areas: a time-series study from 1987 to 2018. Lancet Planetary Health. 2021,5:775-785.

S19 Davidou S, Christodoulou A, Fardet A, Frank K. The holistico-reductionist Siga classification according to the degree of food processing: an evaluation of ultra-processed foods in French supermarkets. Food Funct. 2020;11(3):2026-2039. doi:10.1039/C9FO02271F

S20 de Las Heras-Delgado S, Shyam S, Cunillera È, et al Are plant-based alternatives healthier? A two-dimensional evaluation from nutritional and processing standpoints. Food Res Int. 2023;169:112857. doi:10.1016/j.foodres.2023.112857

S21 de Moraes MM, Oliveira B, Afonso C, et al. An ultra-processed food dietary pattern is associated with lower diet quality in Portuguese adults and the elderly: The UPPER project. Nutrients. 2021;13:4119. doi:10.3390/nu13114119.

S22 Delgado-Rodríguez R, Moreno-Padilla M, Moreno-Domínguez S, Cepeda-Benito A. Food addiction correlates with emotional and craving reactivity to industrially prepared (ultra-processed) and home-cooked (processed) foods but not unprocessed or minimally processed foods. Food Qual Prefer. 2023;110:104961. doi:10.1016/j.foodqual.2023.104961

S23 Derbyshire, E. Are all ‘ultra-processed’foods nutritional demons? A commentary and nutritional profiling analysis. Trends Food Sci Technol. 2019;94:98-104. doi:10.1016/j.tifs.2019.08.023

S24 Detopoulou P, Dedes V, Pylarinou I, et al. Dietary acid load is associated with waist circumference in university students with low adherence to the Mediterranean diet: The potential role of ultra-processed foods. Clin Nutr ESPEN. 2023;56:43-51. doi:10.1016/j.clnesp.2023.05.005

S25 Dickie S, Woods J, Machado P, Lawrence M. Nutrition classification schemes for informing nutrition policy in Australia: nutrient-based, food-based, or dietary-based?. Curr Dev Nutr. 2022;6(8):112. doi:10.1093/cdn/nzac112

S26 Dinu M, Tristan Asensi M, Pagliai G, et al. Consumption of ultra-processed foods is inversely associated with adherence to the Mediterranean diet: a cross-sectional study. Nutrients. 2022;14:2073. doi:10.3390/nu14102073

S27 Estell ML, Barrett EM, Kissock KR, et al. Fortification of grain foods and NOVA: the potential for altered nutrient intakes while avoiding ultra-processed foods. Eur J Nutr. 2022;1-11. doi:10.1007/s00394-021-02701-1

S28 Fardet A, Rock E. How to protect both health and food system sustainability? A holistic ‘global health’-based approach via the 3V rule proposal. Public Health Nutr. 2020;23:3028-3044. doi:10.1017/S136898002000227X

S29 Fardet A, Méjean C, Labouré H, et al. The degree of processing of foods which are most widely consumed by the French elderly population is associated with satiety and glycemic potentials and nutrient profiles. Food Funct. 2017;8:651-658. doi:10.1039/c6fo01495j

S30 Gallegos-Riofrío CA, Waters WF, Carrasco A, et al. Caliata: an Indigenous Community in Ecuador offers lessons on food sovereignty and sustainable diets. Curr Dev Nutr. 2021;5:61-73. doi:10.1093/cdn/nzab009

S31 García S, Pastor R, Monserrat-Mesquida M, et al. Ultra-processed foods consumption as a promoting factor of greenhouse gas emissions, water, energy, and land use: A longitudinal assessment. Sci Total Environ. 2023;891:164417. doi:10.1016/j.scitotenv.2023.164417

S32 Garzillo JMF, Poli VFS, Leite FHM, et al. Ultra-processed food intake and diet carbon and water footprints: a national study in Brazil. Revista de saude publica, 2022;56:6. doi:10.11606/s1518-8787.2022056004551

S33 Grech A, Rangan A, Allman-Farinelli M, et al. A Comparison of the Australian Dietary Guidelines to the NOVA Classification System in Classifying Foods to Predict Energy Intakes and Body Mass Index. Nutrients. 2022;14:3942. doi:10.3390/nu14193942

S34 Gupta S, Hawk T, Aggarwal A, Drewnowski A. Characterizing ultra-processed foods by energy density, nutrient density, and cost. Front Nutr. 2019;6:454858. doi:10.3389/fnut.2019.00070

S35 Gupta S, Rose CM, Buszkiewicz J, et al. Characterising percentage energy from ultra-processed foods by participant demographics, diet quality and diet cost: Findings from the Seattle Obesity Study (SOS) III. BJN. 2021;126:773-781. doi:10.1017/S0007114520004705

S36 Hallinan S, Rose C, Buszkiewicz J, Drewnowski A. Some ultra-processed foods are needed for nutrient adequate diets: linear programming analyses of the Seattle obesity study. Nutrients. 2021;13:3838. doi:10.3390/nu13113838

S37 Hässig A, Hartmann C, Sanchez-Siles L, Siegrist M. Perceived degree of food processing as a cue for perceived healthiness: the NOVA system mirrors consumers’ perceptions. Food Qual Prefer. 2023;110:104944. doi:10.1016/j.foodqual.2023.104944

S38 Julia C, Baudry J, Fialon M, et al. Respective contribution of ultra-processing and nutritional quality of foods to the overall diet quality: results from the NutriNet-Santé study. Eur J Nutr. 2023;62:157-164. doi:10.1007/s00394-022-02970-4

S39 Juul F, dos Santos Simões B, Litvak J, et al. Processing level and diet quality of the US grocery cart: is there an association?. Public Health Nutr. 2019;22:2357-2366. doi:10.1017/S1368980019001344

S40 Juul F, Lin Y, Deierlein AL, et al. Trends in food consumption by degree of process. BJN. 2021;126:1861-1871. doi:10.1017/S000711452100060X

S41 Kesse-Guyot E, Allès B, Brunin J, et al. Environmental impacts along the value chain from the consumption of ultra-processed foods. Nature Sustainability. 2023;6(2):192-202. doi:10.1038/s41893-022-01013-4

S42 Lavigne-Robichaud M, Moubarac JC, Lantagne-Lopez S, et al. Diet quality indices in relation to metabolic syndrome in an Indigenous Cree (Eeyouch) population in northern Québec, Canada. Public Health Nutr. 2018;21(1):172-180. doi:10.1017/S136898001700115X

S43 Liu J, Steele EM, Li Y, et al. Consumption of ultraprocessed foods and diet quality among US children and adults. Am J Prev Med. 2022;62(2):252-264. doi:10.1016/j.amepre.2021.08.014

S44 Maia EG, Passos CMD, Granado FS, et al. Replacing ultra-processed foods with fresh foods to meet the dietary recomendations: a matter of cost?. Cadernos de Saúde Pública. 2022;37:e00107220. doi:10.1590/0102-311X00107220

S45 Marchese L, Livingstone KM, Woods JL, et al. Ultra-processed food consumption, socio-demographics and diet quality in Australian adults. Public Health Nutr. 2022;25(1):94-104. doi:10.1017/S1368980021003967

S46 Martinez-Perez N, Arroyo-Izaga M. Availability, nutritional profile and processing level of food products sold in vending machines in a Spanish public university. Int. J. Environ. Res. Public Health. 2021;18(13):6842. doi:10.3390/ijerph18136842

S47Martinez-Perez C, San-Cristobal R, Guallar-Castillon P, et al. Use of different food classification systems to assess the association between ultra-processed food consumption and cardiometabolic health in an elderly population with metabolic syndrome (PREDIMED-Plus Cohort). Nutrients. 2021;13(7):2471. doi:10.3390/nu13072471.

S48 Martinez-Perez C, Daimiel L, Climent-Mainar C, et al. Integrative development of a short screening questionnaire of highly processed food consumption (sQ-HPF). Int J Behav Nutr Phys Act. 2022;19(1):6. doi:10.1186/s12966-021-01240-6

S49 Mendes C, Miranda L, Claro R, Horta P. Food marketing in supermarket circulars in Brazil: An obstacle to healthy eating. Preventive Med Reports. 2021;21:101304. doi:10.1016/j.pmedr.2020.101304

S50 Mendoza-Velázquez A, Lara-Arévalo J, Siqueira KB, et al. Affordable nutrient density in brazil: nutrient profiling in relation to food cost and NOVA category assignments. Nutrients. 2022;14(20):4256. doi:10.3390/nu14204256

S51 Mignogna C, Costanzo S, Di Castelnuovo A, et al. The inflammatory potential of the diet as a link between food processing and low-grade inflammation: An analysis on 21,315 participants to the Moli-sani study. Clin Nutr. 2022;41:2226-2234. doi:10.1016/j.clnu.2022.08.020

S52 Morales FJ, Mesías M, Delgado-Andrade C. Association between heat-induced chemical markers and ultra-processed foods: A case study on breakfast cereals. Nutrients. 2020;12:1418. doi:10.3390/nu12051418

S53 Otten JJ, Buszkiewicz J, Tang W, et al. The impact of a city-level minimum-wage policy on supermarket food prices in Seattle-King County. Int. J. Environ. Res. Public Health. 2017;14:1039. doi:10.3390/ijerph14091039

S54 Phulkerd S, Dickie S, Thongcharoenchupong N, et al. Choosing an effective food classification system for promoting healthy diets in Thailand: a comparative evaluation of three nutrient profiling-based food classification systems (government, WHO, and Healthier Choice Logo) and a food-processing-based food classification system (NOVA). Front Nutr. 2023;10:1149813. doi:10.3389/fnut.2023.1149813

S55 Phulkerd S, Thongcharoenchupong N, Dickie S, et al. Profiling ultra-processed foods in Thailand: sales trend, consumer expenditure and nutritional quality. Global Health. 2023;19:64. doi:10.1186/s12992-023-00966-1

S56 Pulker CE, Trapp GS, Scott JA, Pollard CM. Alignment of supermarket own brand foods’ front-of-pack nutrition labelling with measures of nutritional quality: An Australian perspective. Nutrients. 2018;10:1465. doi:10.3390/nu10101465

S57 Rizzolo-Brime L, Orta-Ramirez A, Puyol Martin Y, Jakszyn P. Nutritional assessment of plant-based meat alternatives: a comparison of nutritional information of plant-based meat alternatives in Spanish supermarkets. Nutrients. 2023;15:1325. doi:10.3390/nu15061325

S58 Robert M, Shankland R, Bellicha A, et al. Associations between resilience and food intake are mediated by emotional eating in the NutriNet-Santé Study. Journal of Nutr. 2022;152:1907-1915. doi:10.1093/jn/nxac124

S59 Rodrigues VM, Rayner M, Fernandes AC, et al. Nutritional quality of packaged foods targeted at children in Brazil: which ones should be eligible to bear nutrient claims?. Int J Obes. 2017;41:71-75. doi:10.1038/ijo.2016.167

S60 Romero Ferreiro C, Lora Pablos D, Gómez de la Cámara A. Two dimensions of nutritional value: Nutri-Score and NOVA. Nutrients. 2021;13: 2783. doi:10.3390/nu13082783

S61 Rossato SL, Khandpur N, Lo CH, et al. Intakes of unprocessed and minimally processed and Ultraprocessed food are associated with diet quality in female and male health professionals in the United States: A prospective analysis. J Acad Nutr Diet. 2023;123:1140-1151. doi:10.1016/j.jand.2023.03.011

S62 Ruggiero E, Esposito S, Costanzo S, et al. Ultra-processed food consumption and its correlates among Italian children, adolescents and adults from the Italian Nutrition & Health Survey (INHES) cohort study. Public Health Nutr. 2021;24:6258-6271. doi:10.1017/S1368980021002767

S63 Salomé M, Arrazat L, Wang J, et al. Contrary to ultra-processed foods, the consumption of unprocessed or minimally processed foods is associated with favorable patterns of protein intake, diet quality and lower cardiometabolic risk in French adults (INCA3). Eur J Nutr. 2021;60:4055-4067. doi:10.1007/s00394-021-02576-2

S64 Shim JS, Shim SY, Cha HJ, et al. Association between ultra-processed food consumption and dietary intake and diet quality in Korean adults. JAcad Nutr Diet. 2022;122:583-594. doi:10.1016/j.jand.2021.07.012

S65 Siqueira KB, Borges CA, Binoti ML, et al. Nutrient density and affordability of foods in Brazil by food group and degree of processing. Public Health Nutr. 2021;24(14):4564-4571. doi:10.1017/S1368980020004358

S66 Sneed NM, Ukwuani S, Sommer EC, et al. Reliability and validity of assigning ultraprocessed food categories to 24-h dietary recall data. Am J Clin Nutr. 2023;117:182-190. doi:10.1016/j.ajcnut.2022.10.016.

S67 Spiteri SA, Olstad DL, Woods JL. Nutritional quality of new food products released into the Australian retail food market in 2015–is the food industry part of the solution? BMC Public Health, 2018;18:1-10. doi:10.1186/s12889-018-5127-0

S68 Trübswasser U, Talsma EF, Ekubay S, et al. Factors influencing adolescents' dietary behaviors in the school and home environment in Addis Ababa, Ethiopia. Front Public Health. 2022;10:861463. doi:10.3389/fpubh.2022.861463

S69 Valenzuela A, Zambrano L, Velásquez R, et al. Discrepancy between food classification systems: Evaluation of Nutri-Score, NOVA classification and chilean front-of-package food warning labels. Int. J. Environ. Res. Public Health. 2022,19:14631. doi:10.3390/ijerph192214631

S70 Van Dam I, Vandevijvere S. Benchmarking the nutrition-related commitments and practices of major French food companies. BMC Public Health, 2022;22:1435. doi:10.1186/s12889-022-13780-y

S71 Van Dam I, Reimes N, Vandevijvere S. Benchmarking the nutrition-related commitments and practices of major Belgian food companies. Int J Behav Nutr Phys Act. 2022;19:43. doi:10.1186/s12966-022-01269-1

S72 Vandevijvere S, Pedroni C, De Ridder K, Castetbon, K. The cost of diets according to their caloric share of ultraprocessed and minimally processed foods in Belgium. Nutrients. 2020;12:2787. doi:10.3390/nu12092787

S73 Vellinga RE, van Bakel M, Biesbroek S, et al. Evaluation of foods, drinks and diets in the Netherlands according to the degree of processing for nutritional quality, environmental impact and food costs. BMC Public Health. 2022;22: 877. doi:10.1186/s12889-022-13282-x

S74 Vellinga RE, van den Boomgaard I, Boer JM, Different Levels of Ultraprocessed Food and Beverage Consumption and Associations with Environmental Sustainability and All-cause Mortality in EPIC-NL. Am J Clin Nutr. 2023;118(1):103-113. doi:10.1016/j.ajcnut.2023.05.02Siqueira KB, Borges CA, Binoti ML, et al. Nutrient density and affordability of foods in Brazil by food group and degree of processing. Public Health Nutr. 2021;24(14):4564-4571. doi:10.1017/S1368980020004358

S75 Vergeer L, Veira P, Bernstein JT, et al. The calorie and nutrient density of more-versus less-processed packaged food and beverage products in the Canadian food supply. Nutrients. 2019;11:2782. doi:10.3390/nu11112782

S76 Vicente B M, Almeida Bastos A, de Melo CM, et al. Correlation Between Different Dietary Indexes, and Their Association with An Anti-inflammatory Biomarker in Older Adults: An Exploratory Study. Eur J Geriatr Geront. 2023;5(3). doi:10.4274/ejgg.galenos.2023.2022-10-5

S77 Vogliano C, Raneri JE, Maelaua J, et al. Assessing diet quality of indigenous food systems in three geographically distinct solomon islands sites (Melanesia, Pacific Islands). Nutrients. 2020;13:30. doi:10.3390/nu13010030
